# AsCas12a ultra nuclease facilitates the rapid generation of therapeutic cell medicines

**DOI:** 10.1038/s41467-021-24017-8

**Published:** 2021-06-23

**Authors:** Liyang Zhang, John A. Zuris, Ramya Viswanathan, Jasmine N. Edelstein, Rolf Turk, Bernice Thommandru, H. Tomas Rube, Steve E. Glenn, Michael A. Collingwood, Nicole M. Bode, Sarah F. Beaudoin, Swarali Lele, Sean N. Scott, Kevin M. Wasko, Steven Sexton, Christopher M. Borges, Mollie S. Schubert, Gavin L. Kurgan, Matthew S. McNeill, Cecilia A. Fernandez, Vic E. Myer, Richard A. Morgan, Mark A. Behlke, Christopher A. Vakulskas

**Affiliations:** 1 Integrated DNA Technologies, Inc, Coralville, IA USA; 2 Editas Medicine Inc, 11 Hurley St, Cambridge, MA USA; 3 University of California - Merced, 5200 Lake Rd, Merced, CA USA

**Keywords:** Genetic engineering, Targeted gene repair, CRISPR-Cas9 genome editing, Bacterial genetics, CRISPR-Cas9 genome editing

## Abstract

Though AsCas12a fills a crucial gap in the current genome editing toolbox, it exhibits relatively poor editing efficiency, restricting its overall utility. Here we isolate an engineered variant, “AsCas12a Ultra”, that increased editing efficiency to nearly 100% at all sites examined in HSPCs, iPSCs, T cells, and NK cells. We show that AsCas12a Ultra maintains high on-target specificity thereby mitigating the risk for off-target editing and making it ideal for complex therapeutic genome editing applications. We achieved simultaneous targeting of three clinically relevant genes in T cells at >90% efficiency and demonstrated transgene knock-in efficiencies of up to 60%. We demonstrate site-specific knock-in of a CAR in NK cells, which afforded enhanced anti-tumor NK cell recognition, potentially enabling the next generation of allogeneic cell-based therapies in oncology. AsCas12a Ultra is an advanced CRISPR nuclease with significant advantages in basic research and in the production of gene edited cell medicines.

## Abstract (web summary)

The utility of AsCas12a can be limited to poor editing efficiency. Here the authors identify a variant, “AsCas12a Ultra”, that has high on-target specificity demonstrated through editing of clinically relevant T cell genes.

## Introduction

CRISPR-Cas-based genome editing has garnered tremendous interest for enabling efficient, site-specific gene editing with greater ease than prior technologies, such as meganucleases, zinc-finger nucleases, and transcription-activator-like effector nucleases (reviewed in refs. ^1,2^). The most widely used Cas nuclease is derived from *Streptococcus pyogenes* (SpCas9), which was first demonstrated as an RNA-programmable nuclease with robust activity in eukaryotic cells^3–5^. SpCas9 targets NGG protospacer-adjacent motifs (PAM) that significantly restrict its targeting space to genomic regions that are generally GC-rich^3–5^. Alternatively, the Cas12a nuclease derived from *Acidaminococcus sp*. (AsCas12a, formerly AsCpf1) targets TTTV PAM sequences and significantly expands the genome editing targeting space to include AT-rich genomic sequences^6^. In addition to broadening the target space for genome editing, several groups have also shown that AsCas12a is intrinsically more specific than SpCas9^7,8^, likely due to its lower tolerance for guide-target mismatches^9,10^. In contrast, SpCas9 demonstrates poor target site specificity in living cells, which has, in part, delayed its rapid adoption as a clinical therapeutic. As a result, clinical application of SpCas9 typically requires empirical determination of off-target effects and evaluation of guide RNA safety and/or the use of mutant “high-fidelity” Cas9 proteins delivered as ribonucleoprotein complexes^11–19^. The notion of clinical genome editing with Cas12a has garnered interest because of its intrinsic high fidelity^7,8,20^ and short 40–43mer crRNAs^6^, which can be readily chemically manufactured unlike the ~100mer sgRNA of SpCas9 from research to GMP grade. Despite these advantages, the wide adoption of AsCas12a as a genome editing tool has been much lower than that of SpCas9 likely due to its relatively low editing efficiency in living cells^21,22^. Improving on-target editing efficiency of AsCas12a would expand the therapeutic genome editing space, reduce the complexity of guide RNA manufacturing, and alleviate off-target editing concerns.

Cell therapy for cancer is one such example of a field that could benefit greatly from a robust Cas12a solution. While multiple chimeric antigen receptor (CAR) engineered T cell therapies have recently gained FDA approval, they rely on patient-derived cells which greatly impedes manufacturing^23^. Allogeneic cell therapy provides a potential solution, but requires multiplexed genome editing of donor cells to avoid rejection by the patient’s immune system^24^. Though SpCas9 enables multiplex genome editing with high efficiency^25,26^, the introduction of multiple guides for this promiscuous nuclease can greatly increase the risk of unwanted off-target editing and would require an expensive and laborious empirical determination of off-target editing before entry into the clinic. The highly specific Cas12a nuclease addresses this shortcoming but is limited by low editing efficiency^6^. This limits its ability to potentially address a range of devastating diseases that require very efficient editing, such as disruption of the fetal hemoglobin repressor binding site^27^ for sickle cell anemia or to efficiently make multiple edits in immune effector cells for the generation of off-the-shelf cancer immunotherapies^26^.

Here, we show the use of directed evolution in bacteria to identify a highly active AsCas12a mutant, hereafter referred to as “AsCas12a Ultra”. Two-point mutations, M537R and F870L, markedly increased efficacy without compromising the high intrinsic specificity of AsCas12a. Both knockout and knock-in efficiency are elevated by AsCas12a Ultra in both cancer cell lines and in human primary cells (HSPCs, iPSCs, T cells, NK cells), where we observed robust editing efficiencies approaching 100% at several clinically relevant targets. In T cells, we achieved up to 60% knock-in of a single transgene and up to 40% double knock-in. In NK cells, we demonstrate enhanced tumor killing through highly efficient AsCas12a Ultra editing either by knocking out an immune-suppressive cytokine receptor or by knock-in of a CAR. Taken together, AsCas12a Ultra is a robust gene-editing tool, retaining the superior specificity of wild-type AsCas12a while boosting editing across many cell types, which are distinct advantages over the original SpCas9 platform.

## Results

### Directed evolution of AsCas12a with enhanced activity

**Fig. 1:**
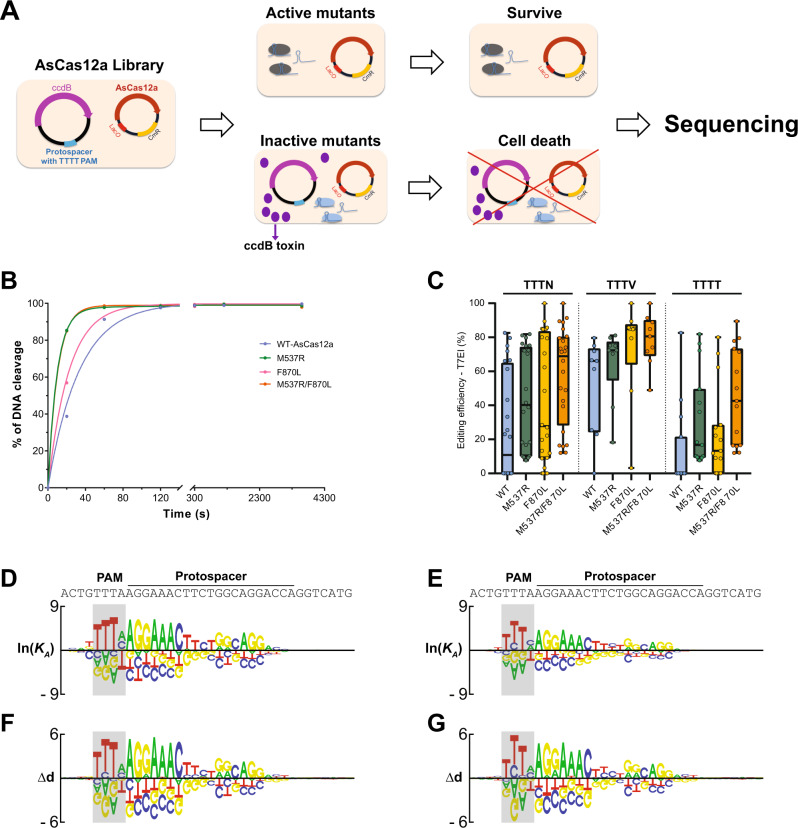
Directed evolution of AsCas12a with enhanced activity. **A** Schematic representation of a bacterial-based variant selection of AsCas12a. *E. coli* cells harboring an inducible toxin-expression reporter were transformed with plasmids encoding AsCas12a with random mutations, which is programmed to target a defined protospacer with TTTT PAM on the reporter. Active AsCas12a variants that can cleave the reporter plasmid enabled cell survival upon arabinose induction. The frequency of AsCas12a variants over multiple rounds of selection was determined by sequencing. **B** The in vitro DNA cleavage activities of WT-AsCas12a, M537R, F870L, and M537R/F870L (AsCas12a Ultra) at a protospacer with TTTT PAM. Pre-assembled AsCas12a RNP (125 nM) was incubated with dsDNA substrate (10 nM). The substrate cleavage percentage at various time points was measured by capillary electrophoresis. **C** Genome editing efficiency of WT, M537R, F870L, and M537R/F870L (AsCas12a Ultra) in HEK293 cells as RNP (5 μM) over 24 sites targeting *CTNNB1* gene (*n* = 24). **D**, **E** The in vitro DNA binding specificity of WT and AsCas12a Ultra measured by *Spec-seq*. **F**, **G** The in vitro DNA cleavage specificity of WT and AsCas12a Ultra measured by *SEAM-seq*. Raw source data are provided in the Source Data File.

To enhance the activity and expand the targeting space of AsCas12a, we used directed evolution in bacteria to isolate mutant proteins that more efficiently cleave target sites that contain TTTT PAM sequences, where WT AsCas12a exhibits low activity (Fig. 1A, Supplementary Table 1)^11^. Briefly, the survival rate of *E. coli* cells was linked to AsCas12a-dependent cleavage of a TTTT PAM-containing target site through the placement of the target sequence on a plasmid that expresses an arabinose-inducible, bacteriostatic toxin (Fig. 1A). We optimized the selection pressure so that a sharp contrast of survival rate was achieved when the PAM is changed from TTTC to TTTT (Supplementary Fig. 1d–e). The basic premise is that mutant AsCas12a variants with enhanced cleavage activity at TTTT PAM sites facilitate better survival and therefore plasmids that express these variants are significantly enriched and easily isolated from surviving *E. coli* cells.

We first performed five rounds of sequential selection using an AsCas12a library containing random mutations in amino acids positions I503–I905. This library was generated using low-fidelity PCR and contains (on average) 5 mutations per kilobase of DNA. Each round of library was deep sequenced with NGS to determine the identity and frequency of each mutation (Supplementary Fig. 1a). Specific point mutations in the library were gradually enriched throughout the selection process, which resulted in two dominant mutants, M537R and F870L (Supplementary Fig. 1a). To further confirm that these enriched mutations are the direct cause of increased cleavage activity, we independently isolated the M537R and F870L mutations by direct cloning and tested them for function in bacteria. Consistent with the results of our enrichment screen, both mutations increased the survival rate of *E. coli* cells at TTTC and TTTT PAM sites, indicating an overall boost in AsCas12a activity (Supplementary Fig. 1d–i). We mapped these amino acid positions to the published crystal structure of AsCas12a^28^, which revealed that the M537 likely interacts directly with the PAM, and thus we speculate that the M537R mutation may have increased DNA binding affinity by promoting stronger interactions with the PAM (Supplementary Fig. 1b). In contrast, the F870L amino acid position is located in the binding interface between AsCas12a and the conserved stem loop of the crRNA. We therefore speculate that the F870L mutation may have increased the rate of formation and/or overall stability of the AsCas12a RNP complex (Supplementary Fig. 1c).

To interrogate the functional significance of each mutation, we purified each mutant protein and measured the intrinsic cleavage activity in vitro. Consistent with what was observed in *E. coli*, both mutants significantly increased the rate of DNA cleavage at TTTT PAM sites in a cell-free context (Fig. 1B, Supplementary Fig. 2a–d, Supplementary Table 2), while maintaining high activity at TTTC PAM sites (Supplementary Fig. 2e–h). We were unable to confirm an increase in the cleavage rate at TTTC PAM sites, presumably due to the limitations of our in vitro system, which lacks resolution for fast reaction kinetics. We next tested these variants in human cells by targeting 24 different sites within the *CTNNB1* locus (including 15 TTTT PAM sequences) as RNP complexes. While 70% of these sites (11/15) have no detectable editing by WT AsCas12a, the M537R and M537R/F870L mutants enabled detectable cleavage at all 15 TTTT PAM sites (Fig. 1C). These mutants also significantly improved editing efficiency at TTTN sites in human cells (Fig. 1C). The F870L mutant in isolation substantially increased editing at 11/15 TTTT sites but showed no benefit for the remaining four TTTT PAM sites. For other sites with canonical TTTV PAM, these variants maintained or improved the targeting efficiency in all cases (Fig. 1C). Among the three variants, the double-mutant (M537R/F870L) had the most consistent improvement in targeting efficiency across all tested sites, whereas the single mutations exhibited more site-dependent variations. We therefore selected the double mutant (M537R/F870L) as AsCas12a Ultra for further characterization.

We next characterized the intrinsic sequence specificity of AsCas12a Ultra in vitro. The DNA cleavage and binding specificities were determined for AsCas12a RNP (WT or Ultra) over a pool of target sites containing up to 4 nt sequential mismatches to the crRNA (Supplementary Fig. 3a, Supplementary Table 2) as determined by SEAM/Spec-seq^10^. The relative DNA binding affinity (ln*K*_*A*_) of nuclease-inactivated AsCas12a (dCas12a, D908A/E993A/D1235A^29^) over mismatched sequences was calculated as the ratio of sequence distribution between dCas12a-bound and unbound DNA fractions. Similarly, the relative cleavage activity (∆d) mediated by AsCas12a-RNP was measured by comparing the distribution between the uncut and input fractions of the library. The measured affinity and activity obtained from two biological replicates (Supplementary Figs. 3b–e) was subjected to non-linear regression to infer the quantitative penalty of mismatches at each position of the target site and presented as motif logos (Fig. 1D–G). We found similar specificity profiles between WT and AsCas12a Ultra for both DNA binding and cleavage. Both enzymes preferentially recognize the TTTV PAM motif with relatively low specificity in positions 1 and 4 of the PAM site. Purine bases are generally disfavored across the PAM, other than the last position where thymidine caused the greatest penalty. Beyond the PAM, the sequence specificity of AsCas12a Ultra over the protospacer region was measured by Spec-seq/SEAM-seq and determined to be nearly identical to WT (Fig. 1D–G). Both enzymes recognize 19 of 21 positions of the protospacer, where the strongest specificity is observed in the seed region (positions 1–7). The characteristic reduction of specificity for AsCas12a in the center of protospacer (positions 9–11) holds true for both enzymes. Consistent with the enhanced intrinsic activity, the penalty of mismatches in the protospacer for binding and cleavage is slightly reduced by AsCas12a Ultra (Supplementary Fig. 3f–g), suggesting that these mutations enhanced non-specific protein-DNA binding affinity without drastically altering the sequence specificity. In summary, our in vitro characterization of both on- and off-target activities revealed that the sequence specificities of AsCas12a Ultra were nearly identical across the entire protospacer and PAM region compared to WT, but with greatly enhanced activity.

### AsCas12a ultra is a highly efficient genome editing enzyme with broad utility

**Fig. 2:**
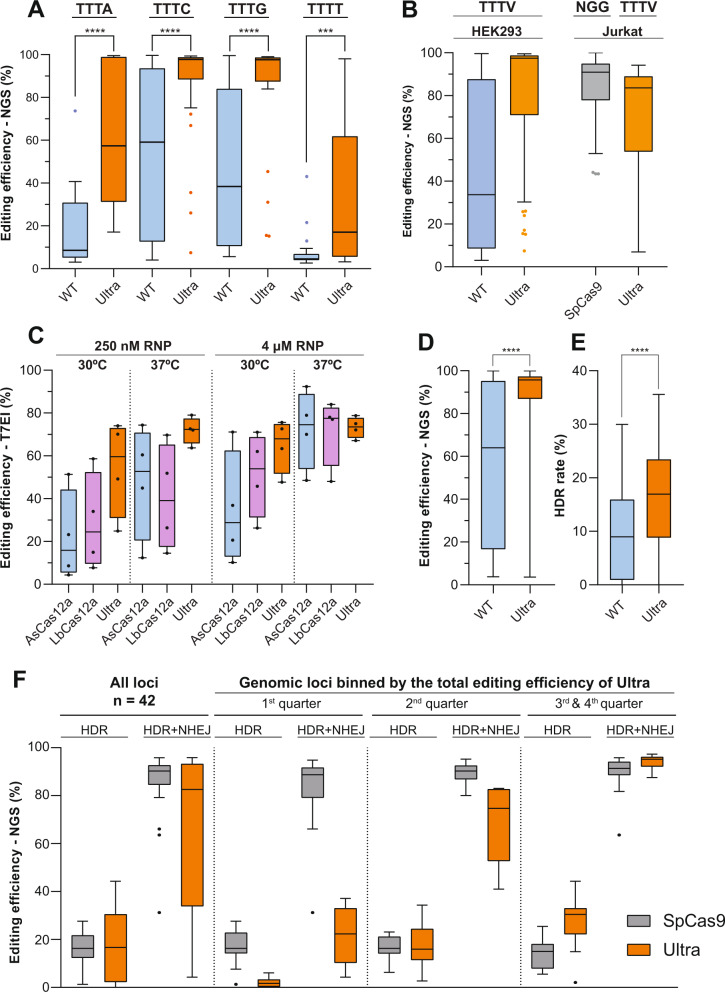
AsCas12a Ultra enabled robust genome editing in human cell lines. **A** Comparison of on-target editing efficiency between WT and AsCas12a Ultra over 96 sites with TTTN PAMs in human HEK293 cells (*n* = 96). **B** Editing efficiency of WT and AsCas12a Ultra with TTTV PAMs (71 of 96) in HEK293 cells (*n* = 71), and performance of WT SpCas9 targeting the same genomic loci with NGG PAMs (*n* = 58). **C** Editing efficiency of WT AsCas12a and LbCas12a, and AsCas12a Ultra over 4 shared target sites with TTTV PAM in HEK293 cells at 30 °C or 37 °C (*n* = 4). **D**, **E** The total editing efficiency (NHEJ + HDR) and the HDR rate mediated by WT or Ultra in Jurkat cells, when ssODN was used as donor template (*n* = 102). **F** Comparison of HDR and total editing efficiency (NHEJ + HDR) between AsCas12a Ultra and SpCas9 when targeting same genome loci using ssODN as donor template (*n* = 42) in Jurkat cells. Paired, two-tailed *t*-test was performed to evaluate statistical significance (****P-value ≤ 0.0001; ***P-value ≤ 0.001; **P-value ≤ 0.01). Raw source data are provided in the Source Data File.

We next systematically evaluated the performance of AsCas12a Ultra in human cells. First, the editing efficiencies mediated by RNP complexes formed with AsCas12a WT or Ultra over 96 target sites with TTTN PAM sequence were evaluated in HEK293 cells by NGS amplicon sequencing (Fig. 2A, Supplementary Table 3). Consistent with in vitro PAM specificity studies (Fig. 1D–G), both enzymes prefer TTTV over TTTT PAM in human cells (Fig. 2A). Most significantly, AsCas12a Ultra universally enhanced the editing efficiency at all tested sites (Fig. 2A). The median editing efficiency of AsCas12a Ultra at canonical TTTV PAM sites (71 of 96) reached 97%, which is ~3-fold higher than WT-AsCas12a (33%) (Fig. 2B). This performance is not dependent on the cellular context, as editing efficiency was also enhanced in Jurkat cells (AsCas12a Ultra median efficiency: 84%), which is comparable to SpCas9 (91%) when targeting matched loci (Fig. 2B). It is critical to note that we intentionally selected a set of highly efficient sgRNAs for SpCas9, whereas the nearest available AsCas12a crRNA without any guide selection was used in this comparison, thus offering an accurate evaluation on the potency of AsCas12a Ultra across divergent genomic space. The AsCas12a Ultra mutations also improved the poor performance of the wild-type enzyme at low temperatures (≤30 °C), where the editing efficiency of AsCas12a Ultra in HEK293 cells outperformed both wild-type As- and LbCas12a at 30 °C and 37 °C (Fig. 2C, Supplementary Table 4). Lastly, we tested whether these mutations can enhance the cleavage efficiency of other published AsCas12a variants with alternative PAMs^30^. We combined the M537R/F870L mutations with the AsCas12a RR (S542R/K607R) and RVR (S542R/K548V/N552R) variants and determined editing efficiency at TATV and TYCV sites within the *HPRT* locus at a suboptimal RNP concentration (0.5 μM) (Supplementary Fig. 4a, Supplementary Table 5). As anticipated, the AsCas12a Ultra mutations elevated the activity for both RR and RVR at their respective PAM sites. The benefit is particularly significant for the low-activity RVR variant, where the median editing efficiency was drastically improved from 14 to 48%, approaching what is achievable with AsCas12a Ultra at canonical TTTV PAMs (70%) and AsCas12a Ultra RR at TYCV PAMs (66%) (Supplementary Fig. 4a). We further compared the gene knockout efficiency mediated by RVR or AsCas12a Ultra RVR in primary T cells by targeting the *TRAC* gene of the T-cell receptor (TCR). Over a wide range of RNP dosages, we demonstrated up to a 10-fold boost of performance by AsCas12a Ultra (Supplementary Fig. 4b). Collectively, our studies conclusively demonstrate that the AsCas12a Ultra mutations provide reliable and consistent performance improvements in a variety of human cells.

Recent studies revealed significant non-specific shredding activity of AsCas12a on single-stranded DNA that occurs after binding and cleavage of the intended double-stranded protospacer^31^. This has raised a potential concern that AsCas12a may be generally incompatible with homology-dependent repair (HDR), where single-stranded DNA oligonucleotide (ssODNs) are preferentially used as donor templates. We therefore tested the performance of AsCas12a Ultra on ssODN-mediated precise editing in human Jurkat cells, which are notorious for poor HDR rates^32^. Interestingly, titration of AsCas12a RNP (0.63–5 μM) with a fixed concentration of ssODN (3 μM) revealed that while a high concentration of AsCas12a facilitates efficient gene knockout (Fig. 2A, B), excess enzyme can negatively affect HDR “knock-in” efficiency for both WT and AsCas12a Ultra (Supplementary Fig. 5a, b). This indicates that the optimal HDR rate for AsCas12a Ultra is achieved using less than saturating levels of enzyme, with peak knockout efficiency in the range of 0.5~2 μM RNP. We next determined the optimal window of DNA insertion within the target site of AsCas12a. At a fixed RNP dosage (5 μM), the highest rate to insert an EcoRI restriction sequence using ssODN was achieved from positions 8–16 of the protospacer (Supplementary Fig. 5c). A sharp drop of efficiency was observed when inserting the EcoRI sequence at PAM distal region (from position 18 and beyond), presumably reflecting the loss of specificity of AsCas12a for mismatches over PAM distal region (Fig. 1D–G), in which the nuclease is anticipated to recut the repaired genome after HDR events, resulting in the accumulation of NHEJ alleles.

Using optimized parameters, we performed a large-scale evaluation of HDR efficiency of AsCas12a Ultra in Jurkat cells. First, we compared the HDR rate of WT and AsCas12a Ultra over 100 target sites from 23 genes (Supplementary Table 6). The significant boost of editing efficiency mediated by AsCas12a Ultra (Fig. 2D) doubled the median HDR rate from 8 to 16% (Fig. 2E). We next compared Ultra and wild-type SpCas9 on HDR over 42 genomic loci (Supplementary Table 7) where the on-target editing efficiency achieved by each nuclease had been previously determined (Fig. 2b). Low-activity crRNAs for AsCas12a Ultra were intentionally included in this comparison, to provide a realistic estimation of HDR efficiency for AsCas12a Ultra over genomic targets with diverse activities. In general, the median HDR efficiencies of the two nucleases are nearly identical (~16%) (Fig. 2F). We next binned the genomic loci based on the total editing efficiency of AsCas12a Ultra. Strikingly, other than the bottom 25% of sites (first quarter) where Ultra has the poor performance of editing (median efficiency ~20%), the HDR rate of AsCas12a Ultra at the remaining sites was equivalent or substantially higher than SpCas9 (Fig. 2F). This is particularly evident when the total editing efficiency of two enzymes are equivalently high (>90%, third and fourth quarter), where AsCas12a Ultra outperformed SpCas9 on precise knock-in (median HDR rate: 29.5% vs 15.2%). Overall, despite having a non-specific ssDNA shredding activity, our data clearly demonstrated that AsCas12a Ultra is equivalent to or greater than as SpCas9 at mediating precise HDR, when ssODN was used as a donor template.

### AsCas12a ultra enables highly efficient and specific genome engineering in clinically relevant primary cells

**Fig. 3:**
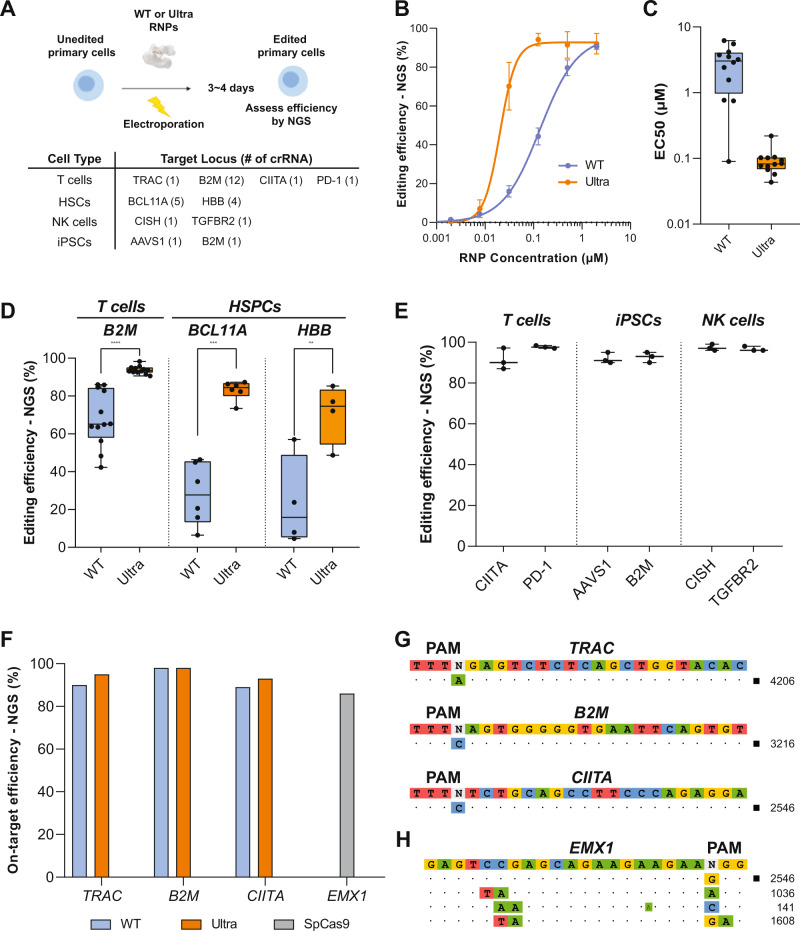
AsCas12a Ultra enables efficient, site-specific genome engineering in primary cells. **A** Overview of AsCas12a gene editing in primary human cells. Primary cells were electroporated with RNP, then expanded for 3–4 days before analysis of indel formation by NGS or knockout efficiency by flow cytometry. **B** The editing efficiency of WT or AsCas12a Ultra at *TRAC* (T-cell receptor alpha constant) locus in T cells across a range of RNP concentrations (*n* = 1). A concentration-response curve was generated to estimate the Half maximal effective concentration (EC50) of the editing. Data are presented as mean values ± SD. **C** EC50s of WT or Ultra over 12 crRNAs targeting the *B2M* locus in primary T cells (*n* = 12). See Supplementary Fig. 6 for individual guide concentration-response curves. **D** Editing efficiency of WT or Ultra (1 µM) across a range of different guides at *B2M* (Beta-2-microglobulin) locus in T cells (*n* = 12), and *BCL11A* (B-cell lymphoma/leukemia 11A, *n* = 5) and *HBB* (Beta-Globin) locus in HSPCs (*n* = 4). Each data point represents a different guide at a unique location within the locus. (*P*-value < 0.0001, =0.0002, and =0.0094 respectively). **E** Editing efficiency of WT or AsCas12a Ultra (1 µM RNP) across a variety of clinically relevant loci in T cells, NK cells, HSPCs, and iPSCs. Targets not previously defined are *CIITA* (Class II Major Histocompatibility Complex Transactivator), *PD-1* (Programmed cell death 1), *AAVS1* (Adeno-associated virus integration site 1), *CISH* (Cytokine inducible SH2 containing protein), and *TGFBR2* (Transforming growth factor, beta receptor II). Results of the optimal guide RNA (*n* = 1) targeting each locus were plotted. Data are presented as mean values ± SD. **F** On-target editing efficiency of WT and AsCas12a Ultra (1 µM RNP) over three-high-activity crRNAs that were used for the off-target study. **G** Off-target discovery of AsCas12a Ultra by GUIDE-seq. **H** Off-target sites of SpCas9 recovered by GUIDE-seq as a positive control. Paired, two-tailed *t*-test was performed to evaluate statistical significance (****P-value ≤ 0.0001; ***P-value ≤ 0.001; **P-value ≤ 0.01). Raw source data are provided in the Source Data File.

To evaluate the translational potential of AsCas12a as an editing platform for ex vivo engineered cell therapies, we next tested its performance in human primary cells (Fig. 3A). We first compared the efficiencies of WT and AsCas12a Ultra over a range of RNP concentrations in primary T cells using a top-performing guide targeting the *TRAC* locus^33^ (Supplementary Table 8). Our results revealed that AsCas12a Ultra is significantly more potent than wild-type AsCas12a (Fig. 3B), achieving comparable editing efficiencies with ~6.7-fold lower RNP concentration than wild-type (EC50: 135 nM vs. 20 nM). We next measured the EC50 of editing efficiency for both WT and AsCas12a Ultra over 12 different sites targeting the *B2M* locus (Supplementary Fig. 6). Impressively, AsCas12a Ultra enhanced the editing efficiency by ~37-fold on average (median EC50: 3 μM vs. 80 nM, Fig. 3c), with a greater benefit over those sites poorly targeted by WT enzyme (Supplementary Fig. 6). Based on these data, we reasoned that 1 μM AsCas12a RNP (~10X of EC50) would result in ~90% editing efficiency over most of the sites in T cells (Fig. 3D). Similar improvement enabled by AsCas12a Ultra (1 μM RNP) was found in HSPCs when targeting *BCL11A* enhancer and *HBB* loci (Fig. 3D). We further observed consistently high editing (>85%) using AsCas12a Ultra across a variety of clinically relevant loci in a multiple primary cell types, including NK cells, T cells, iPSCs, and HSPCs (Fig. 3D, E, Supplementary Table 8). Through optimization of AsCas12a Ultra RNP concentration and AAV6 titer we were able to achieve up to 32% knock-in efficiency of mCherry at *B2M* locus in HSPCs (Supplementary Fig. 7a–d). High editing in iPSCs and HSPCs is notable, as multiple engineered cell therapies can be derived from these cells. Taken together, the data indicate that AsCas12a Ultra is a robust nuclease for genome editing in primary cells.

To characterize the off-target activity of AsCas12a Ultra, we first performed GUIDE-seq experiments in HEK293 cells. Among 6 guides tested (Supplementary Table 9), we found a variable and low levels of signal for WT enzyme even at on-target sites. With similar sequencing depth, robust on-target signals were recovered for AsCas12a Ultra with limited off-target sites, suggesting this enzyme retained high on-target specificity at a genome-wide level (Supplementary Fig. 8). As off-target editing is a significant concern for the development of clinical cell therapies, we next examined the off-target profiles of AsCas12a Ultra in primary human T cells. Since guide activity is a limiting factor of the GUIDE-seq assay, we intentionally selected crRNAs with the highest on-target activity from our screens of the *B2M*, *TRAC*, and *CIITA* loci, in order to maximize the recovery of both on- and off-target signals for AsCas12a Ultra. Of note, these guides are intrinsically more potent than the others, where 90% editing efficiency was achieved even using WT enzyme (Fig. 3F). GUIDE-seq results indicated no detectable tag incorporation for any of the guides (Fig. 3G). As a positive control, we performed GUIDE-seq on WT-SpCas9 using a sgRNA targeting EMX1 (Fig. 3H). The same set of off-targets was recovered as previously published, therefore validating the performance of our GUIDE-seq procedure in primary T cells. Overall, AsCas12a Ultra has high specificity in primary cells, a particularly crucial property for cell therapy applications.

Previous effort of engineering AsCas12a resulted in enCas12a (E174R/S542R/K548R)—a variant reported with enhanced on-target activity and broadened PAM compatibility^30^. Here, we benchmarked AsCas12a Ultra against enCas12a by comparing both activity and specificity. We first determined the optimal PAM sequences for enCas12a. We performed Spec-/SEAM-seq on enCas12a and comprised individual point mutations (i.e. E174R, S542R, and K548R). Our results show that while all point mutations enhanced AsCas12a’s non-specific DNA binding affinity to varying degrees (Supplementary Fig. 9a–c), only the S542R mutation expanded the repertoire of targetable PAMs, achieving this by specifically increasing the affinity to bind cytosine at the third position of the PAM (Supplementary Fig. 9b). In contrast, K548R specifically reduced affinity to cytosine at the second position of the PAM region, thus making the PAM preference of this variant more stringent than the WT nuclease (Supplementary Fig. 9c). Combining all point mutations substantially increased the non-specific DNA binding affinity over WT nuclease, therefore dampening the specificity of enCas12a over all 256 PAM sequences systematically (Supplementary Fig. 9d).

To ensure an accurate interpretation of the PAM preference for enCas12a, we directly compared the results of Spec-/SEAM-seq against the PAM activities measured by lentiviral-based tiling assay ex vivo^34^. Over those 57 of 256 PAM sequences covered by the tiling assay, we found a great correlation between the two approaches (Spearman’s Rho ≥ 0.94), thus validating the accuracy of our in vitro results (Supplementary Fig. 9e–f). We next reviewed the top 10 preferred PAM sequences for each nuclease (Supplementary Fig. 9g). For enCas12a, the optimal PAM remained as the canonical TTTV, which is followed by TTCC, TTTT, TTCA, and VTTV. As expected, the expanded PAM activity of enCas12a over WT nuclease at TTCC and TTCA originated from S542R. Since K548R selects against a PAM sequence with a cytosine at the second position, adding this point mutation reduced the activity of AsCas12a at a TCTV PAM, which is naturally tolerated by the WT nuclease as the best sub-optimal PAM site (Supplementary Fig. 9c, g). Based on this observation, we anticipated that removing K548R from enCas12a will further expand the targetable PAM sites. Our comprehensive PAM analysis shows that enCas12a is an improved version of S542R with elevated non-specific binding affinity derived from E174R. Therefore, any AsCas12a variant containing S542R, such as RR (S542R/K607R) and RVR (S542R/K548V/N552R), will lead to an altered PAM preference over WT nuclease. Since AsCas12a Ultra mutations substantially enhanced the activities of both RR and RVR (Supplementary Fig. 4), we predicted that AsCas12a Ultra RR and RVR will be good alternatives for enCas12a with even broader targetable PAM sequences.

**Fig. 4:**
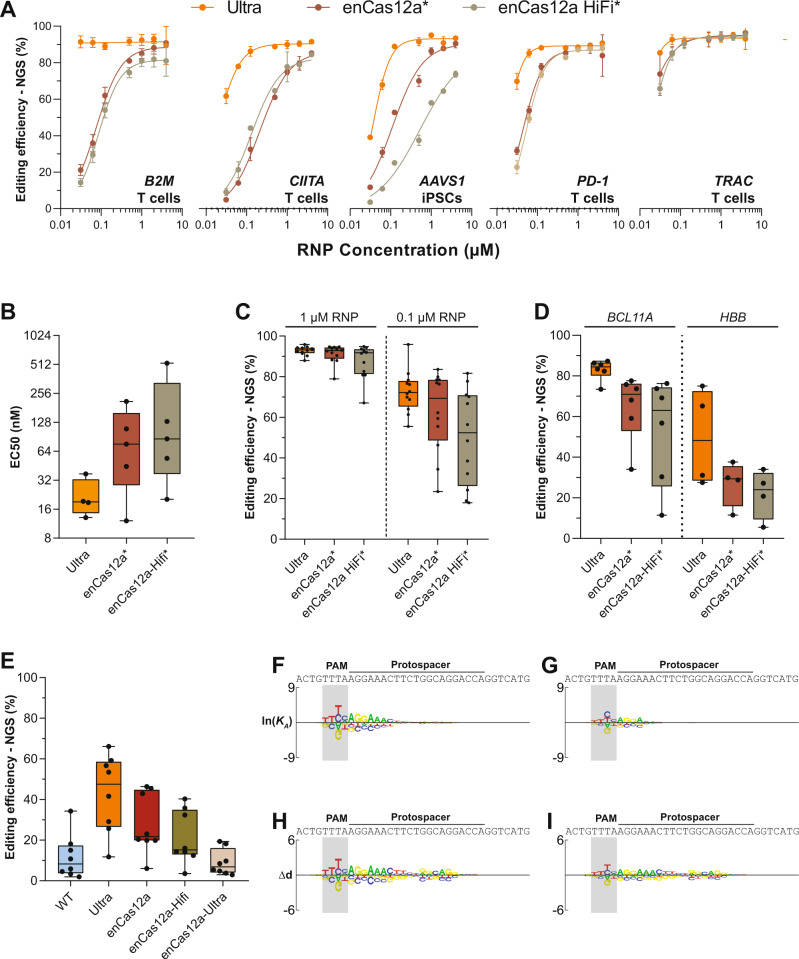
AsCas12a Ultra is more specific and potent than enCas12a. **A** The on-target editing efficiencies of Ultra, enCas12a, and enCas12a-Hifi across a wide range of RNP dosages at 5 target sites in primary cells (*n* = 5). Data are presented as mean values ± SD. *: enCas12a and Hifi with published NLS sequence. **B** The estimated EC50 of editing for each nuclease (*n* = 4). AsCas12a Ultra is ~4-fold more active than enCas12a (19 nM vs. 76 nM). **C** Editing efficiencies of AsCas12a Ultra, enCas12a and enCas12a-Hifi over 12 target sites of *B2M* locus in primary T cells (*n* = 12). Each data point represents a different guide at a unique location within the locus. **D** Editing efficiencies of AsCas12a Ultra, enCas12a and enCas12a-Hifi targeting *BCL11A* (*n* = 5) and *HBB* (*n* = 4) loci in HSPCs. Each data point represents a different guide at a unique location within the locus. **E** Editing efficiencies of WT, AsCas12a Ultra, enCas12a, enCas12a-Hifi and enCas12a-Ultra RNPs (50 nM) over 8 target sites in HEK293 cells (*n* = 8). Note: nucleases with identical NLS sequences were used in this experiment. **F**, **G** DNA binding specificities of enCas12a (**F**) and enCas12a-Ultra (**G**) as measured by Spec-seq/SEAM-seq. **H**, **I** DNA cleavage specificities of enCas12a (**H**), and enCas12a-Ultra (**i**) as measured by Spec-seq/SEAM-seq. While the general pattern of specificity remained the same, the intrinsic DNA sequence specificities of enCas12a and enCas12a-Ultra are systematically lower than AsCas12a Ultra (Fig. 1E, G) across the entire protospacer. Raw source data are provided in the Source Data File.

With the AsCas12a Ultra PAM accurately defined, we next compared the on-target activities of enCas12a and AsCas12a Ultra over the same set of target sites with TTTV PAMs in cells. We first purified the previously described enCas12a and enCas12a-Hifi (+N282A) proteins^30^, and measured the editing efficiencies over five targets in human primary cells over a wide range of RNP concentrations (Fig. 4A). In every case, AsCas12a Ultra enabled more efficient editing than both enCas12a and enCas12a-Hifi. Analysis of concentration-response curves revealed that AsCas12a Ultra is ~4-fold more active than enCas12a (median EC50: 19 nM vs. 76 nM, Fig. 4B). We next expanded the comparison of activity over a total of 19 target sites in primary cells. At equal RNP concentrations, AsCas12a Ultra consistently outperformed both enCas12a and the Hifi variant (Fig. 4C, D). Since the selection of nuclear localization signal (NLS) can influence the on-target potency of CRISPR nucleases^35^, we further compared the activity of these AsCas12a variants all with the same NLS in HEK293 cells over 8 targets (Fig. 4E). Consistent with the original report, enCas12a doubled the editing of WT nuclease (median efficiency: 15% vs. 30%). However, it is still only half as active as AsCas12a Ultra (60%). Our findings show that AsCas12a Ultra is more potent than enCas12a, serving as the most active AsCas12a variant described so far.

Lastly, we evaluated the targeting specificity of enCas12a via Spec-seq/SEAM-seq. As the trade-off for higher non-specific DNA binding affinity, enCas12a displayed reduced binding (Fig. 4F) and cleavage specificities (Fig. 4H) than both WT (Fig. 1D–F; Supplementary Fig. 10a, c) and AsCas12a Ultra nucleases (Fig. 1E, G; Supplementary Fig. 10b, d) over the entire protospacer. Since the DNA binding affinity of enCas12a is greater than AsCas12a Ultra (Supplementary Figs. 3f, 10b), it might be expected that the on-target activity of enCas12a should be higher as well. Given the apparent conflict with our editing result in human cells, we hypothesized that over-stacking mutations with enhanced affinity for AsCas12a will penalize on-target efficiency, by trapping the nuclease at off-target sites within the vast space of the human genome, thus diluting out the available RNP molecules required to achieve efficient editing at the on-target site. To test this hypothesis, we combined the AsCas12a Ultra mutations with those of enCas12a to generate a new nuclease referred to as enCas12a-Ultra (E174R/S542R/K548R + M537R/F870L), which further elevated the non-specific DNA binding affinity with even greater sequence promiscuity than enCas12a (Fig. 4G, I; Supplementary Fig. 10e–f). Consistent with our hypothesis, while enCas12a-Ultra displayed the best DNA cleavage in vitro, it is less efficient than either AsCas12a Ultra or enCas12a in human cells (Fig. 4E). Our observation here is in complete agreement with the recent on-/off-target analysis of SpCas9 in *E. coli*, where the number of off-target sites for a given sgRNA has been shown to reduce the on-target activity proportionally^36^. Given the drastic size differences among various genomes, we anticipated that this “off-target dilution effect” is even more pronounced in the human genome with RNP delivery, which may provide one layer of explanation regarding why many CRISPR nucleases worked efficiently in bacteria failed to generate sufficient on-target edits in human cells. In conclusion, our result demonstrated that a balance between activity and specificity for a given CRISPR nuclease is required to achieve efficient and precise genome editing in human cells. Among many developed CRISPR enzymes, AsCas12a Ultra is a fine-tuned nuclease specifically tailored for ex vivo therapeutic applications, where both activity and specificity must both be optimized to the maximum extent.

### AsCas12a Ultra enables efficient one-step generation of engineered T cells

**Fig. 5:**
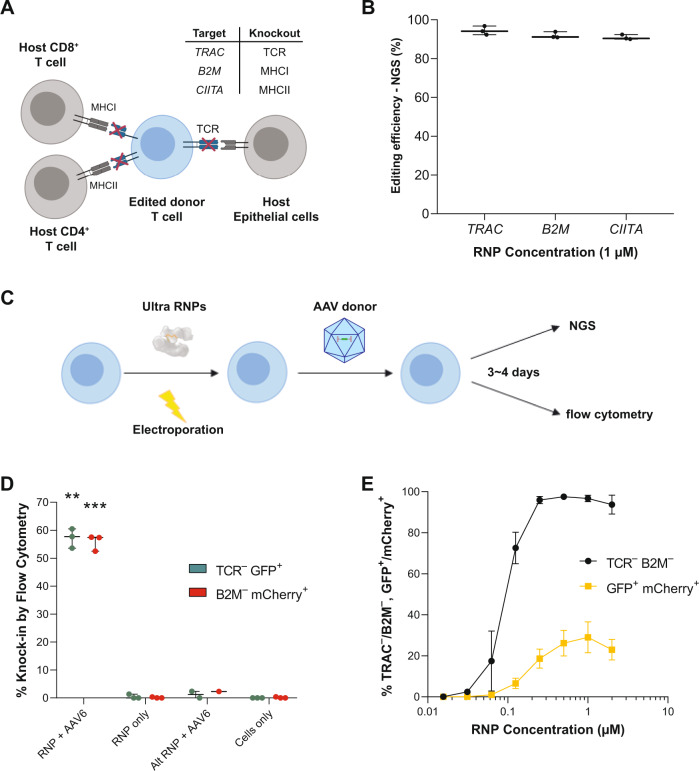
AsCas12a Ultra enables efficient one-step generation of engineered T cells. **A** Potential editing strategy for the generation of allogenic T cells. Multiplexed knock-out of *TRAC*, *B2M*, and *CIITA* to reduce the risk of host-versus-graft rejection and graft-versus-host disease responses, respectively. **B** Simultaneous triple knockout of *TRAC*, *B2M*, and *CIITA* (Class II Major Histocompatibility Complex Transactivator) by AsCas12a Ultra (1 µM RNP). The optimal guide RNA (*n* = 1) targeting each locus was used for multiplex editing. Data are presented as mean values ± SD. **C** Workflow for generation of allogenic T cells with site-specific transgene knock-in by AsCas12a Ultra. The activated T cells were electroporated with Ultra RNPs and transduced with AAV6 carrying a donor template post-electroporation, then analyzed 3–4 days later for transgene expression by flow cytometry. **D** The knock-in efficiency of a fluorescent reporter at either *TRAC* or *B2M* locus using 1 µM RNP and 1 × 10^5^ vg/cell AAV6. The optimal gRNA targeting each locus (n = 1) was used for this experiment. Alternative RNPs (Alt RNP, i.e., *TRAC* RNP + *B2M* AAV or *B2M* RNP + *TRAC* AAV) were used as negative control to rule out any non-specific integration of AAV donor. Data are presented as mean values ± SD. Paired, two-tailed t-test was performed to evaluate statistical significance (**P-value = 0.0012; ***P-value = 0.0008). **E** Simultaneous double knock-in of fluorescent reporters at *TRAC* and *B2M* across a range of RNP concentrations. The optimal gRNA targeting each locus (*n* = 1) was used for this experiment. Raw source data are provided in the Source Data File.

The next generation of engineered allogeneic T cell therapies will involve multiplex editing to enhance the stealth, specificity, and persistence of these cells in vivo. For example, simultaneous knockout of major histocompatibility complexes I and II, which are both displayed on T cells^37^ and at elevated levels in the context of the tumor microenvironment^38^, theoretically enables engineered cells to evade host-versus-graft rejection, whereas knockout of the TCR theoretically prevents a graft-versus-host reaction (Fig. 5A). To evaluate the suitability of AsCas12a to facilitate the generation of triple knockout T cells, we assessed the multiplexed editing efficiency of AsCas12a Ultra across these three loci (*B2M*, *CIITA*, *TRAC*). Using previously identified guides, AsCas12a Ultra RNPs targeting all three loci (1 μM total RNP) were simultaneously delivered via electroporation to activated T cells. Interestingly, NGS analysis revealed an average of 93 ± 2% indel formation at all three loci (Fig. 5B).

We next systematically optimized HDR conditions in T cells using Adeno-associated virus serotype 6 (AAV6) to deliver donor templates coding for fluorescent reporter genes (Supplementary Table 10). AAV6 is widely reported as an efficient vector for donor template delivery to T cells because it is non-integrating and has a natural tropism for leukocytes. Several parameters were varied to maximize knock-in, including RNP concentration, location of knock-in, the multiplicity of infection (MOI), and homology arm length (Supplementary Fig. 11a–e). Following electroporation with RNPs, activated T cells were transduced with AAV6 vectors and assessed for knock-in efficiency 4 days post-electroporation (Fig. 5C). As observed with ssODNs in Jurkat cells, excess AsCas12a Ultra RNP facilitates efficient knockout but reduced knock-in efficiency (Supplementary Fig. 11C). Optimal knock-in efficiency is achieved below saturating levels of enzyme (Supplementary Fig. 11a, c). Knock-in efficiency correlated with knockout efficiency for both WT and AsCas12a Ultra, suggesting the activity of nuclease is a major determinant of knock-in efficiency (Supplementary Fig. 11c, f). High titers of AAV6 vector also reduced knock-in efficiency, potentially due to viral toxicity (Supplementary Fig. 11d). We found the optimal AAV6-based knock-in can be achieved using 1 µM AsCas12a Ultra RNP and an AAV6 titer of 1 × 10^5^ vg/cell, yielding either 57% *TRAC*^-^*EGFP*^+^ cells or 56% *B2M*^-^*mCherry*^+^ cells (Fig. 5D), and used 10-100-fold less RNP compared to the WT nuclease (Supplementary Fig. 11f) to achieve maximal knock-in of these transgenes. Notably, simultaneous delivery of multiple RNPs and AAV6 vectors at optimal conditions resulted in >90% double knockout of *TRAC*/*B2M* and up to 38% double knock-in of *EGFP/mCherry* (Fig. 5E, Supplementary Fig 11g). Given the high knockout and knock-in efficiencies, AsCas12a Ultra is a robust enzyme for making multiplex edits in T cells.

### AsCas12a ultra enables efficient one-step generation of allogeneic CAR-NK cells

**Fig. 6:**
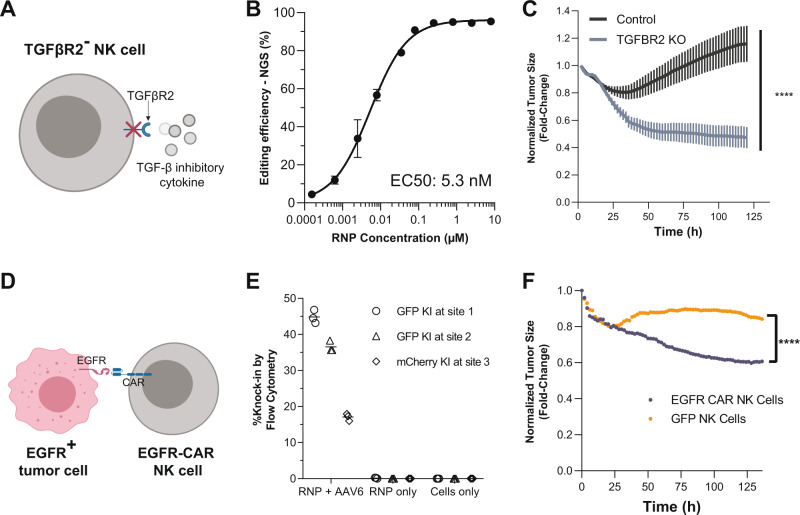
AsCas12a Ultra enables efficient one-step generation of allogenic CAR-NK cells. **A** Potential editing strategy for the generation of edited NK cells to overcome tumor microenvironment. Knocking out *TGFBR2* (Transforming growth factor-beta receptor type 2) prevents inactivation by tumor-derived TGF-β1. **B** Editing efficiency of *TGFBR2A* across a range of RNP concentrations in NK cells derived from three different donors. Data are presented as mean values ± SD. **C** Tumor spheroid killing mediated by *TGFBR2* knock-out NK cells. Engineered NK cells were co-cultured with SK-OV-3 ovarian tumor spheroids at effector-to-target cells ratio of 10:1 (E:T). The spheroid size was monitored over time by Incucyte real-time cell analysis as a proxy for NK cytotoxicity. Two-way ANOVA was performed to evaluate statistical significance (****P-value ≤ 0.0001). Data are representative of five independent experiments using six unique NK cell donors. **D** Graphical depiction of EGFR CAR NK cell recognition of an EGFR^+^ tumor cell. **E** Knock-in efficiency of a fluorescent reporter at either *TRAC* (Target site 1 and Target site 2) or *B2M* (Target site 3) loci using 4 μM RNP and 2.5 × 10^4^ vg/cell AAV6 titer carrying the transgenes. **F** Tumor spheroid killing mediated by GFP^+^ or αEGFR CAR^+^ NK cells. Engineered NK cells were co-cultured with EGFR^+^ PC-3 spheroids at effector-to-target cell ratios of 2:1 (E:T). The spheroid size was monitored over time by Incucyte real-time cell analysis as a proxy for NK cytotoxicity. Two-way ANOVA was performed to evaluate statistical significance (****P-value ≤ 0.0001). Raw source data are provided in the Source Data File.

NK cells hold great promise for immunotherapy because they have natural anti-cancer cell targeting and are not associated with graft-versus-host disease^39^. Similar to T cells, NK cell anti-tumor activity can be blocked by inhibitor cytokines such as TGF-β^40^, which can be abundant in the tumor microenvironment^40^. Thus, engineering an NK cell therapy that is resistant to the immunosuppressive effects of TGF-β is highly desirable. We used AsCas12a Ultra to knockout one of the subunits of the TFG-β receptor, *TGFBR2* (Fig. 6A). We first assessed editing at *TGFBR2* using AsCas12a Ultra across a range of RNP concentrations (Fig. 6B), revealing that AsCas12a Ultra mediated highly efficient editing (EC50: 5.2 nM) of *TGFBR2* across three different donors, which is noteworthy given reports^41^ of donor variability significantly affecting NK cell editing (Fig. 6B). We next demonstrated that *TGFBR2* knockout NK cells kill SK-OV-3 ovarian tumor spheroids more efficiently than control unedited NK cells in the presence of 10 ng/mL TGF-β at an effector to target ratio of 10:1 (Fig. 6C). In support of this observation, we further demonstrated that *TGFBR2* knock-out NK cells experience lower levels of SMAD2/3 phosphorylation than control unedited NK cells after stimulation with 10 ng/mL TGF-β (Supplementary Fig. 13a), providing a biochemical mechanism to support the functional improvement observed in tumor killing assay (Fig. 6C).

Like T cells, NK cells can be re-directed to tumors using CARs^39^. To enhance NK cell anti-tumor reactivity, we knocked-in a CAR that targets epidermal growth factor receptor (αEGFR-CAR) to the *TRAC* locus (Fig. 6D). To achieve efficient knock-in, we first tested the condition optimized with T cells, including the use of the AAV6 vectors for insertion at two sites at *TRAC* locus and one site at *B2M* locus. After optimizing for the appropriate titer of AAV6 (Supplementary Fig. 12a), and the timing post-editing to evaluate knock-in efficiency for notably slower dividing NK cells in comparison to T cells (Supplementary Fig. 12b–d), we were able to achieve 45% knock-in efficiency of fluorescent reporters at *TRAC* locus (Fig. 6E, Supplementary Fig. 13b) with minimal background signal when RNP or AAV6 vector was used alone (Supplementary Fig. 13c, d). We next performed the EGFR-CAR knock-in under the optimized condition (4 μM RNP and 2.5 × 10^4^ vg/cell AAV6). We then used these αEGFR-CAR^+^ NK cells (Supplementary Fig. 13e) in our tumor-killing assays as well as GFP knock-in NK cells to serve as our negative control, with each cargo inserted into the same site in the *TRAC* locus (Supplementary Fig. 13f). When αEGFR-CAR^+^ NK cells were co-cultured with EGFR^+^ PC-3 prostate tumor spheroids, the spheroid size significantly decreased compared to co-culture with GFP control knock-in NK cells (Fig. 6F). This trend held for all effector-to-target ratios, suggesting the αEGFR-CAR knock-in considerably enhances NK cell cytotoxicity (Supplementary Fig. 13g–h). Our data demonstrate that AsCas12a Ultra can be used to edit and knock-in a clinically relevant transgene that enhances effector cell function.

## Discussion

We demonstrated efficient editing with AsCas12a Ultra, approaching 100% at several clinically relevant target sites across multiple and diverse primary human cell types. AsCas12a Ultra is a potent genome editing solution, achieving high editing at >10-fold lower RNP concentration than wild-type AsCas12a^7^ without compromising the enzyme’s characteristic specificity^9,42^. In the first clinical report of the use of SpCas9 to edit genes in human cells, three genes (*TRAC, TRBC, and PDCD1*) were subject to ex vivo editing in primary human T cells, which were subsequently expanded and infused into cancer patients^43^. These investigators reported low (15–45%) individual gene knockout and when multiplexed gene editing was attempted, the resulting three-gene edited cell products were under 5% of total cells administered to patients. By comparison, 93% simultaneous disruption of three genes was achieved in this study using AsCas12a Ultra. Importantly, previous investigators had reported multiple genotoxicity events, such as off-target gene disruption at *CLIC2* (chloride intracellular channel 2); a transcriptional regulator (ZNF609) and a long inter-genic non-protein coding RNA (LINC00377). The long-term consequences of these events are unknown. Comparing this published report of SpCas9 fidelity at the *TRAC* locus (nine detected off-targets) to our data with AsCas12a Ultra (0 detected off-targets) in T cells support our premise that AsCas12a Ultra may have significant advantages as a clinical genome editing reagent.

Recent reports called into question the utility of AsCas12a to mediate robust HDR, where it was observed that AsCas12a exhibits non-specific single-stranded DNA cleavage activity after binding^31,33,44^, raising concern that AsCas12a is not compatible with HDR-mediated targeted integration. Our results invalidate this concern and in fact, demonstrate robust knock-in is possible with AsCas12a using either ssODNs or AAV6 as donor templates. However, our data do not rule out that ssDNA cleavage activity may explain why higher RNP concentrations boost knockout efficiency, but not knock-in efficiency. Using AAV6, knock-in efficiency reached up to 60% in T cells, 50% in NK cells, and 30% in HSPCs. We observed nearly 40% double knock-in efficiency in T cells, which represents greater than a twofold improvement over a previous report using wild-type LbCas12a^33^. In NK cells, we observed knockout efficiencies above 90% and show that the *TGFBR2* edit significantly enhances tumor killing in a model intended to mimic the tumor microenvironment of difficult-to-treat solid tumors. We also show knock-in efficiencies up to 50% using a single transgene. Furthermore, we demonstrate, for the first time, site-specific insertion of an anti-tumor antigen-directed CAR with AsCas12a Ultra significantly increases the anti-tumor activity of NK cells. These data demonstrate the clinical potential of the AsCas12a Ultra platform for making engineered NK cells for cancer immunotherapy.

Our data support AsCas12a Ultra as an ideal nuclease for researchers and clinicians, in that it retains both high activities, generally approaching >90% editing, as well as a specificity profile that is superior to that of SpCas9. In addition, the shorter guide RNAs for AsCas12a Ultra presents researchers with a more economical way to apply this platform using RNP delivery, as it is much less onerous to procure a 40-mer guide than the 100-mer guide required for SpCas9. Combine this guide advantage with the observed high potency of AsCas12a Ultra, with as low as 10 nM RNP required to achieve high editing in primary cells, this nuclease is therefore an attractive option for large-scale screening studies, such as whole-genome knockout analyses. AsCas12a Ultra shows notably higher specificity and efficacy across all cell types tested compared to other engineered Cas12a variants in the field^42^ and we demonstrate that this is likely explained by AsCas12a Ultra’s superior on-target specificity. This observation manifests as greater potency since fewer sites in the genome will be saturated with off-target binding. While demonstrating higher editing efficiency than other reported Cas12a mutants, it seems likely that the activity-enhancing mutations of AsCas12a Ultra can be ported to most Cas12a orthologs and engineered PAM variants if targeting beyond the canonical PAM is required. We believe that these attributes of AsCas12a Ultra also make it a promising nuclease for therapeutic applications. Specifically, we are applying AsCas12a Ultra as an ex vivo approach to editing HSPCs to upregulate fetal hemoglobin production as a treatment for sickle cell anemia^45^ as well as developing off-the-shelf, NK cell-based immunotherapies for treating cancer^46^.

Taken together, AsCas12a Ultra maximizes editing efficiency without compromising specificity, greatly expanding the gene-editing toolbox for researchers and clinicians regardless of cell type. These advances open the door for this nuclease to help patients reap the benefits of gene editing.

## Methods

### Directed evolution of AsCas12a in *E. coli*

The bacterial selection of AsCas12a was adapted from previous work for identifying SpCas9 with enhanced specificity (HiFi-Cas9)^11^. *E. coli* BW25141 (DE3) was transformed with pIDT-ccdB-HPRT38346-TTTT plasmid, and a single colony with ampicillin resistance was grown in SOB medium to prepare electrocompetent cells using ice-cold 10% glycerol.

To generate AsCas12a library with random mutations, the wild-type gene sequence was amplified with low-fidelity PCR (GeneMorph II, Agilent) using primer *Cpf1_PID_mut_fwd* and *Cpf1_PID_mut_REV* (Supplementary Table 1). The PCR product was column-purified, size-selected, and recovered from a 1% 1X TAE agarose gel, and sub-cloned into pACYTDuet-1 vector by Gibson assembly^47^. The assembly reaction was purified (Qiaquick PCR purification kit, Qiagen), and the eluted DNA plasmids were delivered into ElectroMAX DH5-E Competent Cells (Thermo) by electroporation. Transformed *E. coli* cells were recovered in SOC, plated on 245 cm^2^ LB agar with 25 μg/mL chloramphenicol, and incubated at 37 °C overnight. To collect colonies, each LB plate was washed with 3 × 20 mL of LB medium, followed by centrifugation at 4700 × *g* for 20 min. The plasmid library was extracted using ZymoPURE II Plasmid Kits (Zymo) and stored at 4 °C.

AsCas12a mutants with enhanced activity were sequentially enriched over five rounds of selections. Approximately 10^6^ *E. coli* cells (BW25141DE3: pIDT-ccdB-HPRT38346-TTTT) were transformed with AsCas12a library in each round of selection by electroporation. Cells were allowed to recover in SOB medium for 1.5 h (30 °C, 250 rpm), followed by adding 1 mM IPTG to induce the expression of AsCas12a for 1 h, and then plated on LB-chloramphenicol medium with 2% L-arabinose. Plasmids from survived cells were extracted and used for the subsequent round of selection. The mutagenized region of AsCas12a within each round of library was amplified using primer *Cpf1_PID_nextera_F* and *Cpf1_PID_nextera_R* (Supplementary Table 1), and pair-end sequenced (2 × 151-bp) as Illumina Nextera library on a MiSeq instrument. The sequencing reads were aligned to the reference amplicon with the wild-type coding sequence of AsCas12a. Mutations were counted and normalized to the total sequencing depth of each position.

### Protein expression and purification

DNA sequences encoding wild-type or mutant AsCas12a (Supplementary Table 11) were cloned into pET28a vector by Gibson assembly^11^. For protein expression, a single transformed *E. coli* BL21(DE3) colony was inoculated into 20 mL LB medium supplemented with 50 μg/mL kanamycin, and grown overnight at 37 °C, 250 rpm. The overnight culture was transferred to 1 l TB medium with kanamycin, grown at 37 °C, 250 rpm for ~2–3 h until OD600 reached 0.6. The culture was chilled at 4 °C for 30 min prior to induction with 1 mM IPTG, and further incubated at 18 °C, 250 rpm for 12~18 h.

The recombinant AsCas12a protein was purified as previously described for SpCas9^11^. Briefly, *E. coli* cells were harvested by centrifugation, and homogenized with Emulsiflex-C3 high-pressure homogenizer (Avestin, Ottawa ON, Canada). The AsCas12a protein in clarified lysate was sequentially purified using immobilized metal affinity chromatography (HisTrap HP, GE Healthcare) and heparin chromatography (HiTrap Heparin HP, GE Healthcare). Purified protein was concentrated and dialyzed against storage buffer (20 mM TrisHCl, 300 mM NaCl, 0.1 mM EDTA, 50% Glycerol, 1 mM DTT, pH 7.4) overnight. The protein concentration was measured by NanoDrop using extinction coefficient at 143,940 M^−1^cm^−1^, diluted to 60 μM, and stored at −20 °C.

### Cell culture

All cells were grown in humidified 37 °C, 5% CO incubators. Human cell lines HEK293 and Jurkat were obtained from ATCC and used for the development work of AsCas12a Ultra. HEK293 cells were cultured in Dulbecco’s Modified Eagle Medium supplemented with 10% FBS and 100 U/mL penicillin-streptomycin solution (Thermo Fisher Scientific). Jurkat cells were maintained in RPMI-1640 medium with 10% FBS and 100 U/mL penicillin-streptomycin solution. CD4^+^ or CD8^+^ T cells were isolated from peripheral blood mononuclear cells as previously described. Cells were frozen in cryopreservation media at a density of 20 × 10^6^ cells/mL. Upon thawing, T cells were activated with anti-D3/anti-CD28 Dynabeads (ThermoFisher Scientific) for 48 h. Cells were maintained at 1.3 × 10^6^ cells/mL in proprietary base media supplemented with IL-2, IL-7, and IL-15. After removal of Dynabeads, T cells were expanded for an additional 72 h before electroporation. NK cells were isolated from CD3-depleted peripheral blood mononuclear cells as previously described, then frozen in cryopreservation media at 50 × 10^6^ cells/mL. Upon thawing, NK cells were activated with G-Rex plates (Wilson Wolf). Cells were maintained in proprietary base media supplemented with IL-15. Media was refreshed every eight days, with cells harvested on day fourteen of culture for electroporation. CD34^+^ HSPCs were isolated from cord blood, and then frozen in cryopreservation media at a density of 10 × 10^6^ cells/mL. HSPCs were gated on CD34 with a measured population purity of >95% CD34+. Upon thawing, HSPCs were maintained at 1.5 × 10^6^ cells/mL in proprietary base media supplemented with SFC, TPO, and FLT3. iPSCs were cultured on Vitronectin-coated 96-well plates in E8 media supplemented with Clone R and prepared for electroporation as described previously^48^. Briefly, Vitronectin (Stem Cell Technologies) was added to plates by pipetting and mixing gently without disturbing the coating on the bottom of the plate. 100 µL of the E8 plus CloneR (Stem Cell Technologies) was added to the plate and put the plate in the 37 C incubator. Lonza P3 nucleofection buffer was brought to room temperature. After obtaining the cell count, iPSCs were spun down at 115 × *g* for 3 min at room temperature. The supernatant was removed and cells resuspend the cell pellet in P3 buffer (20 µL per well). Then 3 µL of precomplexed RNP per well was added directly into the nucleofection plate, 20 µl of cells were then added to the well and mixed by pipetting up and down gently five times. The 23 µL mixture was nucleofected using the CA-137 Lonza program. Immediately after nucleofection, 80 µL of E8 media supplemented with CloneR was added to wells containing cells in the nucleofection plate, then fully transferred to a culture plate and left in a 37 C incubator. No media was changed for 48 h post nucleofection. After that, wells were refreshed with fresh E8 daily. Cells were then harvested for gDNA extraction for analysis by NGS. iPSCs were gated on Oct4, Nanog, and Tra-160 and demonstrated >95% purity.

### Electroporation

To edit human cell lines, RNP was complexed using purified AsCas12a proteins and chemically synthesized RNA oligonucleotides (Integrated DNA Technologies) at 1:1.2 ratio in 1× PBS. RNP was incubated at room temperature for >10 min prior to electroporation with the Amaxa Nucleofector 96-well Shuttle (Lonza). 0.2 × 10^6^ cells resuspended in 20 μL of Lonza buffer were mixed with 5 μL RNP and 3 μM Cas12a Electroporation Enhancer (IDT) and electroporated according to Lonza specifications (HEK293 cells: SF buffer, DS-150 pulse code; Jurkat: SE buffer, CL-120 pulse code). Cells were harvested 72 h post-electroporation for analysis.

To edit primary cells, RNP was complexed using purified AsCas12a proteins and chemically synthesized RNA oligonucleotides (Integrated DNA Technologies) at 1:2 ratio in H1-50 buffer (10 mM N-2-hydroxyethylpiperazine-N′2-ethanesulfonic acid, 150 mM NaCl, 0.5 mM tris(2-carobxyethyl) phosphine, pH 7.5). RNP was incubated at room temperature for >10 min prior to electroporation with the Amaxa Nucleofector 96-well Shuttle (Lonza). 0.1–0.2 × 10^6^ cells resuspended in 20 μL Lonza buffer were mixed with 3 μL of RNP, then electroporated with the optimal pulse code (T cells: P2 buffer, CA-137 pulse code; NK cells: P2 buffer, CA-137 pulse code; HSPCs: P2 buffer, CA-137 pulse code, iPSCs: P3 buffer, CA-137 pulse code). Electroporated cells were immediately transferred to a 96-well plate with 180 μL of pre-warmed media for incubation. For knock-in experiments, T cells or NK cells were transduced with AAV6 (Sirion Biotech) containing the cargo of interest at the denoted MOI within 30 min after electroporation. All AAV6 knock-in experiments followed the Lonza format procedures for RNP only experiments. Edited cells were then analyzed 4 days later for cargo expression by flow cytometry using the appropriate channel and gating for the fluorescent marker of interest^11^. Cells were harvested 96 h post-electroporation for analysis.

### GUIDE-Seq

GUIDE-Seq in HEK293 cells was performed as previously described^15^. Briefly, 0.35 × 10^6^ cells were resuspended in 20 μL buffer SF and mixed 10 μL reaction mix including 400 ng AsCas12a expression plasmid, 100 ng linear double-stranded DNA fragment encoding the crRNA, and 36 pmole of GUIDE-Seq oligo tag. Cells were electroporated using Lonza Amaxa Nucleofector 96-well Shuttle (Lonza) with DS-150 pulse code, transferred and grown in a 6-well plate for 72 h. The genomic DNA was harvested and purified using GeneJET Genomic DNA Purification Kit (Thermo), and fragmented by Covaris sonication. The sequencing library was prepared as previously described and sequenced on an Illumina MiSeq instrument at ~1–2 million reads per sample (2 × 151-bp, 8-bp index 1, 16-bp index 2).

For T-cells, 4 × 10^6^ cells were resuspended in 80 μL P2 buffer and were mixed with 16 μL of 8 μM RNP and 4 μL of 50 μM double-stranded oligodeoxynucleotides (Supplementary Table 9). Cells were electroporated in Lonza Amaxa Nucleofector 96-well Shuttle (Lonza) with CA-137 pulse code. Electroporated cells were immediately transferred to a 96-well plate with 180 μL of pre-warmed media. Media was refreshed 48 h post-electroporation. 96-hours post-electroporation cells were harvested for genomic DNA extraction. The purified genomic DNA was fragmented by sonication, and prepared for sequencing on a MiSeq instrument with ~2 million reads^15^. The reads were mapped to the human reference genome to determine the sequencing count at each putative off-target sites tagged by GUIDE-seq dsODN as previously described^11^.

### Quantification of genome editing by next-generation sequencing

To quantify editing and HDR frequencies using NGS, libraries were prepared using an amplification-based method as described previously^40^. In short, the first round of PCR was performed using target-specific primers, and the second round of PCR incorporates P5 and P7 Illumina adapters to the ends of the amplicons for universal amplification. Libraries were purified using Agencourt® AMPure® XP system (Beckman Coulter, Brea, CA, USA), and quantified with qPCR before loading onto the Illumina® MiSeq platform (Illumina, San Diego, CA, USA) for paired-end sequencing using V2 chemistry (2 × 150). Data were demultiplexed using Picard tools v2.9 (https://github.com/broadinstitute/picard) and analyzed using a custom pipeline. Paired reads were merged into extended amplicons (flash v1.2.11) before being aligned against the GRCh38 genomic reference (minimap2 v2.12). Reads were assigned to targets in the multiplex primer pool (bedtools tags v2.25) and re-aligned to the target, favoring alignment choices with indels near the predicted cut site(s). At each target, editing was calculated as the percentage of total reads containing an indel within an 8 bp window of the cut site for Cas9 or a 9 bp window from the −3 position of the Cas12a PAM distal cut site.

### Flow cytometry

Cells were centrifuged at 526 × *g* for five minutes, resuspended in staining buffer, incubated at 4 °C for 30 min, and resuspended in FACS buffer in preparation for flow cytometry. Staining buffer consisted of the following antibodies diluted in FACS Buffer: APC anti-human HLA-A/B/C (BioLegend Clone W6/3, 1:100 dilution), BV421 anti-human TCR-α/β (BioLegend Clone IP26, 1:100 dilution) For EGFR CAR staining, cells were initially labeled with biotinylated human EGFR (Sino Biological, 1:100 dilution), then stained with streptavidin-conjugated FITC (ThermoFisher, 1:200 dilution). Data were acquired using the LSRFortessa (Becton Dickinson) and analyzed using FlowJo V10.

### In vitro cleavage assay for individual DNA sequences

The protospacer used in the bacterial screen was ordered as gBlock dsDNA fragment to evaluate the cleavage activity of WT or AsCas12a mutants. The gBlock was amplified using primer *HPRTCF_fwd* and *HPRTCF_rev* (Supplementary Table 2) and purified using either AmPure XP beads (Beckman Coulter) or Qiaquick PCR purification kit (Qiagen). The cleavage reactions were initiated by titrating assembled AsCas12a RNP into DNA substrate (10 nM) in 1× reaction buffer (25 mM TrisHCl, pH 7.4, 150 mM KCl, 10% Glycerol, 5 mM MgCl_2_, and 1 mM DTT). The reactions were sampled at various time points and quenched using 50 mM EDTA. After digesting the AsCas12a protein by protease K, the percentage DNA cleavage in the reactions was determined by capillary electrophoresis (Fragment Analyzer, Agilent).

### Spec-seq/SEAM-seq DNA binding and cutting assays

Spec-seq and SEAM-seq assay methods beyond those mentioned in the “Results” section were performed as described previously^10^. To begin with, a ssDNA library containing a random 4-bp window across the protospacer was ordered as individual Ultramers (Supplementary Table 2) and pooled equally for klenow extension. The dsDNA library was purified (Qiagen MinElute column) and concentrated to 1 μM in buffer EB (10 mM TrisHCl, pH 8.5).

To determine the binding and cleavage specificity, WT or nuclease-inactivated AsCas12a (400 nM) was incubated with crRNA (480 nM) in 1× reaction buffer for 10 min at 37 °C to assemble the ribonucleoprotein complex (25 mM TrisHCl, pH 7.4, 150 mM KCl, 10% Glycerol, 5 mM MgCl_2_, and 1 mM DTT). The assembled RNPs were incubated with 50 nM DNA library for 1 h at 37 °C in 1× reaction buffer supplemented with 40 ng/μL Salmon Sperm DNA (Invitrogen 15632011). For Spec-seq (with dAsCas12a), the binding reaction was resolved on a 4–20% native PAGE in 1× Tris-Glycine buffer supplemented with 5 mM MgCl_2_ at room temperature. Both RNP bound and unbound fractions were excised. DNA in each fraction was extracted by electroelution (Millipore D-Tube™ Dialyzer Midi, MWCO 3.5 kDa) and purified (Qiagen MinElute PCR purification kit) for downstream PCR amplification. For SEAM-seq (with WT-AsCas12a), the cleavage reaction was treated with protease K at room temperature for 10 min to digest AsCas12a protein. The uncut fraction was purified and prepared for PCR reaction. Of note, a mock digestion reaction using buffer alone was performed, and the recovered DNA was purified and sequenced to determine the sequence distribution in the initial library (i.e., Input fraction).

The purified DNA fractions were amplified using KAPA HiFi PCR kit (Roche) to add Illumina sequencing adapters (Supplementary Table 2). A 10 μL qPCR reaction was performed first (1X KAPA PCR buffer supplemented with 0.5X EVA Green, Biotium) to estimate the minimal number of cycles to amplify each sample, which is defined as the end of linear amplification phase of qPCR. Samples were then amplified in a 90 μL reaction with appropriate cycles, and purified by AmPure XP beads (Beckman Coulter). DNA libraries were quantified using the Qubit dsDNA HS assay kit (ThermoFisher) and pooled equally for sequencing. Each library was sequenced to ~10–20 million single-end reads (83-bp) using NextSeq 500/550 High Output Kit v2.5 (75 Cycles) on an Illumina NextSeq instrument.

### Genomic DNA extraction

Genomic DNA of human cancer cell lines was extracted as previously described using QuickExtract DNA extraction solution (Epicentre)^49^. For primary cells (HSPCs, T cells, iPSCs, and NK cells), genomic DNA was isolated using the Agencourt DNAdvance Kit (Beckman Coulter) according to the manufacturer’s instructions and quantified using Quant-iT PicoGreen dsDNA Assay Kit (Thermo Fisher Scientific).

### Tumor spheroid assay

Key reagents include Sony SH800Z, Incucyte S3 (Essen Bioscience), Cellometer Auto 2000 Cell Viability Counter (Nexcelom), RPMI-1640 (Thermo 72400047), Fetal Bovine Serum, heat-inactivated (Thermo 10082147), PC-3 (ATCC CRL-1435), NucLight Red Lentivirus (Essen Bioscience 4625), Trypsin-EDTA, 0.05% (Thermo 25300054), MEM Non-Essential Amino Acids Solution, 100X (Thermo 11140050), AO/PI Viability Stain (Fisher CS201065ML), Corning 96-well Clear Round-bottom Ultra-Low Attachment Microplates (Fisher 07-201-680).

PC-3 cells were transduced using NucLight Red (NLR) lentivirus; cells expressing the transduced construct were sorted by FACS using a Sony SH800Z to isolate NLR^+^ PC-3 line. To initiate spheroid assays, NLR^+^ PC-3 cells were trypsinized and resuspended in RPMI-1640 supplemented with 10% FBS and 0.1 mM non-essential amino acids, hereafter referred to as complete spheroid media. 5000 cells were seeded into each well of a 96-well round-bottom ultra-low attachment plate in 100 µl total and the plate was centrifuged at 234 × *g* for 10 min. Cells were incubated at 37 °C undisturbed to allow spheroid formation. On the fifth day after seeding, NK cells (7 days post-electroporation) were counted, centrifuged at 234 × *g,* and resuspended at the appropriate concentration in complete spheroid media; a dilution series was made to produce lower effector-to-target (E:T) ratios. Here, E:T ratios represent the number of effector cells divided by the number of tumor cells initially seeded, not the number of tumor cells present after spheroid formation. 100 µl of the NK cell suspension was carefully added to each well, to avoid disturbing the spheroids. Plates were placed in the Incucyte S3 and incubated at 37 °C for 30 min before imaging. Plates were imaged every two hours for 6 days. Images were acquired using bright field and red fluorescent channels. Total Red Object Integrated Intensity was used as a proxy for spheroid size. Normalized spheroid size represents the size of a spheroid at a given timepoint as a fraction of its size at the first imaging timepoint after NK cells were added.

### Reporting summary

Further information on research design is available in the Nature Research Reporting Summary linked to this article.

## Supplementary information

Supplementary Information

Reporting Summary

## Data Availability

Raw sequencing files are deposited at SRA under BioProject PRJNA730915. Source data are provided with this paper.

## Source data

Source Data
