# Author Correction: AsCas12a ultra nuclease facilitates the rapid generation of therapeutic cell medicines

**DOI:** 10.1038/s41467-021-24770-w

**Published:** 2021-07-19

**Authors:** Liyang Zhang, John A. Zuris, Ramya Viswanathan, Jasmine N. Edelstein, Rolf Turk, Bernice Thommandru, H. Tomas Rube, Steve E. Glenn, Michael A. Collingwood, Nicole M. Bode, Sarah F. Beaudoin, Swarali Lele, Sean N. Scott, Kevin M. Wasko, Steven Sexton, Christopher M. Borges, Mollie S. Schubert, Gavin L. Kurgan, Matthew S. McNeill, Cecilia A. Fernandez, Vic E. Myer, Richard A. Morgan, Mark A. Behlke, Christopher A. Vakulskas

**Affiliations:** 1 Integrated DNA Technologies, Inc, Coralville, IA USA; 2 Editas Medicine Inc, 11 Hurley St, Cambridge, MA USA; 3 University of California - Merced, 5200 Lake Rd, Merced, CA USA

**Keywords:** Genetic engineering, Targeted gene repair, CRISPR-Cas9 genome editing, Bacterial genetics, CRISPR-Cas9 genome editing

Correction to: *Nature Communications* 10.1038/s41467-021-24017-8, published online 23 June 2021.

The original version of this Article incorrectly listed Liyang Zhang, John A. Zuris, Ramya Viswanathan, Jasmine N. Edelstein, Rolf Turk, Bernice Thommandru and H. Tomas Rube as equally contributing authors under one group. There were two groups of authors that had equally contributed.

This has now been corrected in both the PDF and HTML versions of the Article.

