# Supplementary material for: AsCas12a ultra nuclease facilitates the rapid generation of therapeutic cell medicines
Source: Nat Commun. 2021 Jun 23;12:3908. doi: 10.1038/s41467-021-24017-8 (PMC8222333; doi:10.1038/s41467-021-24017-8)
Supplement: Supplementary file 1 — Supplementary Information [file 41467_2021_24017_MOESM1_ESM.pdf]

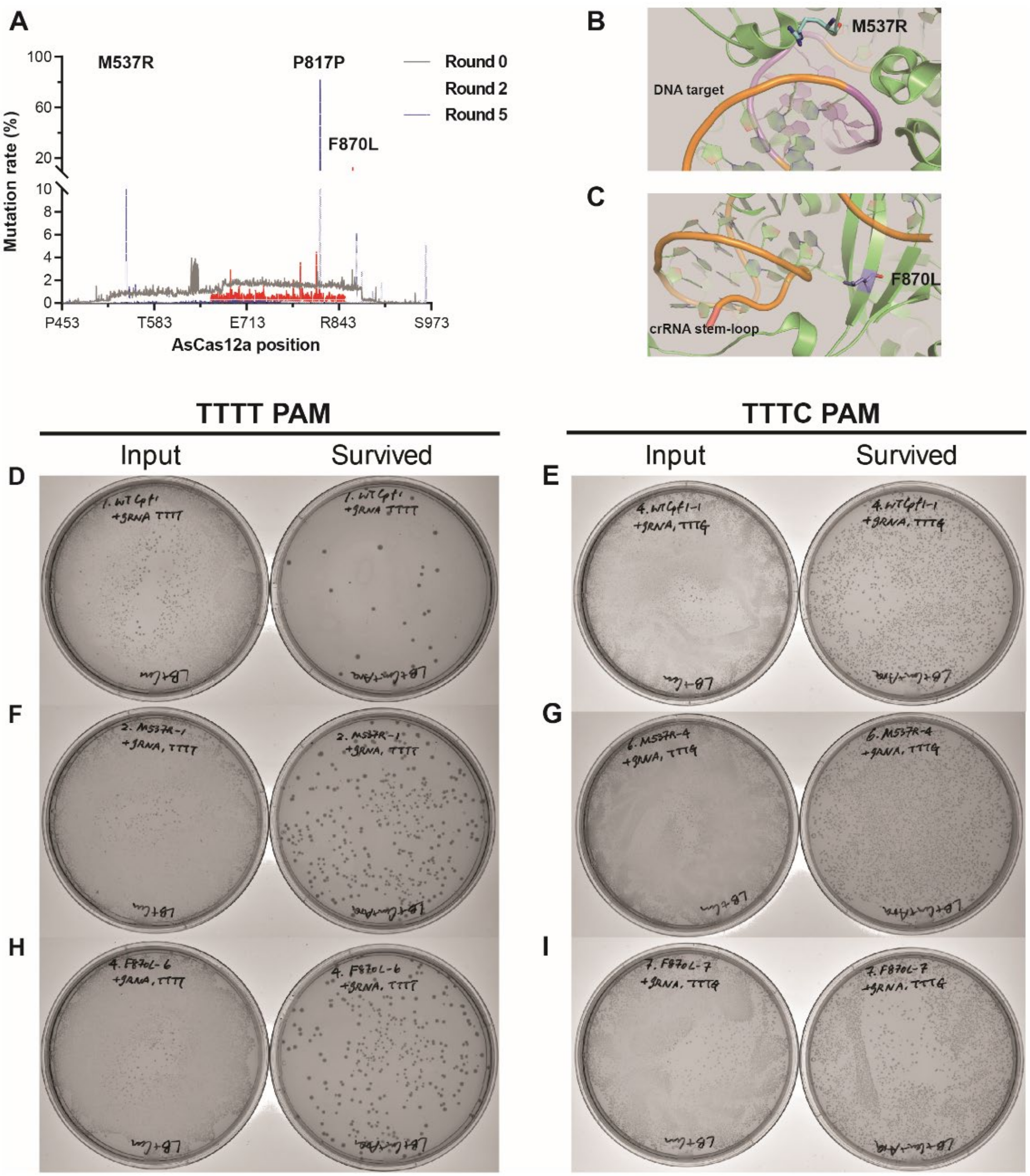


**Supplementary Fig. 1 Identification and validation of AsCas12a mutants with enhanced activity in *E.coli*. a** Frequency of mutations across the mutated region of AsCas12a gene over multiple rounds of selection. **b** Mapping M537R (M537R) on the crystal structure of AsCas12a (PDB: 5B43). The PAM region of DNA target is highlighted in purple. (C) Mapping the F870L on the crystal structure of AsCas12a (PDB: 5B43). **d-i** Activities of WT-AsCas12a, M537R and F870L in bacterial section assay with TTTC or TTTT PAM sequences. Higher survival rate upon arabinose selection ("Survived") relative to the transformation control (no induction, "Input") indicates a mutant with enhanced cleavage activity. Raw source data are provided in an online supplementary data file.

Figure S2

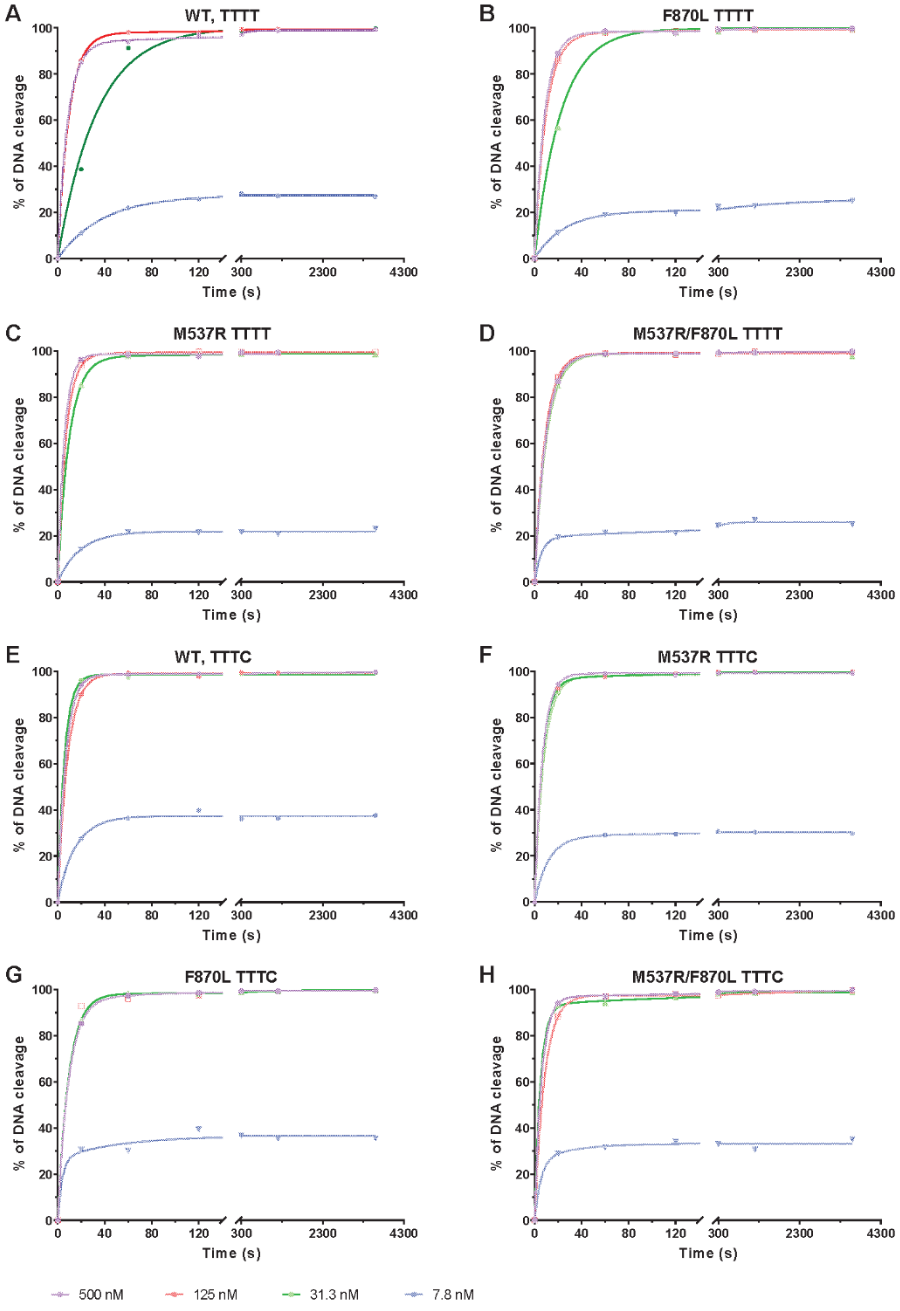


**Supplementary Fig. 2 The *in vitro* cleavage activity of AsCas12a variants.** The assembled AsCas12a RNPs were titrated into dsDNA substrates (15 nM) containing the target site used bacterial selection with TTTC or TTTT PAM sequences. Cleavage reactions were sampled at various time-points and the substrate cleavage was determined by capillary electrophoresis. protein identity and PAM sequence of DNA substrate used in each assay are listed on the top of each figure.

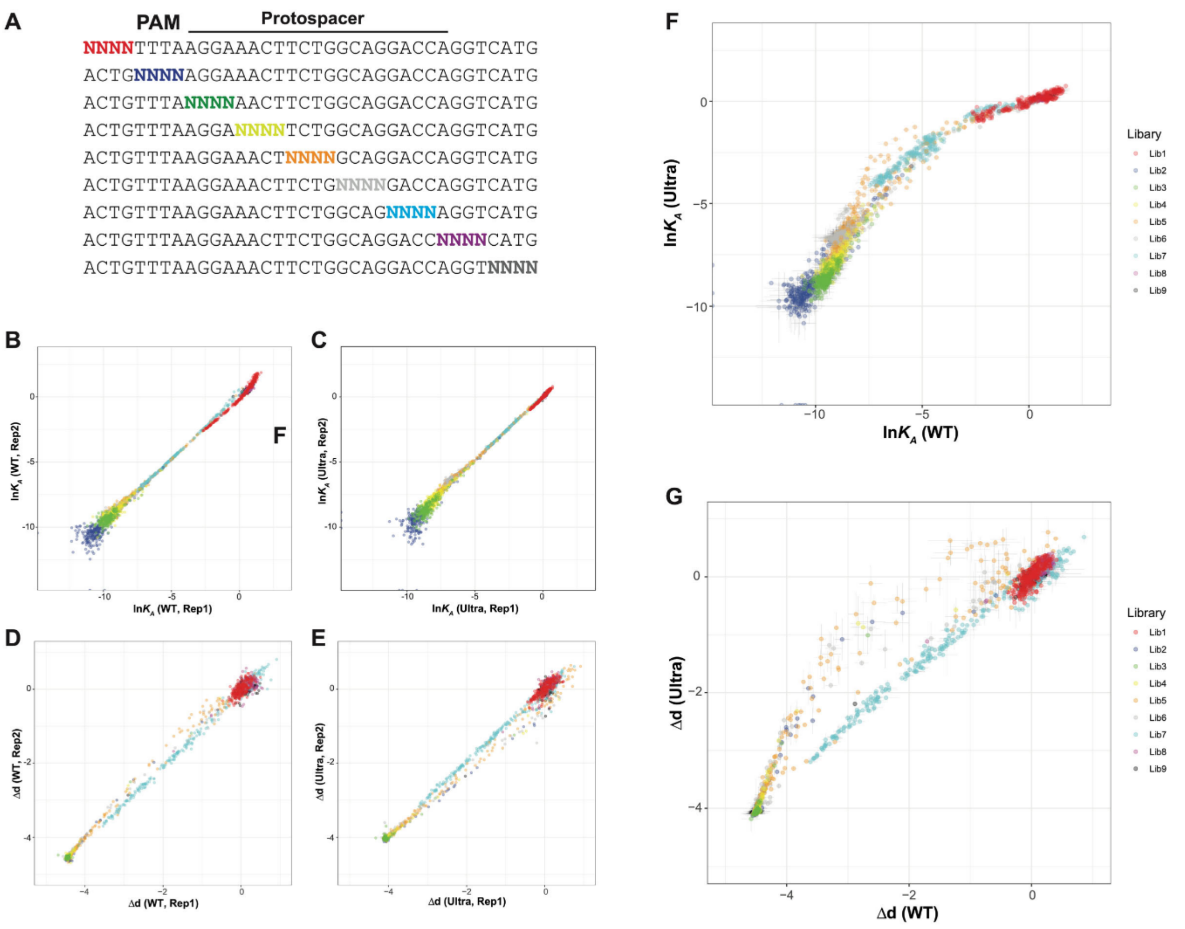


**Supplementary Fig. 3 Measuring the intrinsic DNA binding and cleavage specificities of WT- and AsCas12a-Ultra by Spec-seq/SEAM-seq pair**. **a** Library design with randomized region for Spec-seq/SEAM-seq. **b** Relative DNA binding affinity of nuclease-deactivated WT-AsCas12a (dAsCas12a: D908A/E993A/D1235A) over DNA sequences with mismatches in the library over two biological replicates. **c** Same as (b) but for nuclease-deactivated AsCas12a-Ultra (dAsCas12a-Ultra: D908A/E993A/D1235A/M537R/F870L). **d-e** Relative cleavage activity of WT and Ultra over DNA sequences in the library over two biological replicates. **f** Comparison of relative DNA binding affinity between WT and Ultra for each DNA sequence in the library. **g** Same as (f) but for relative DNA cleavage activity.

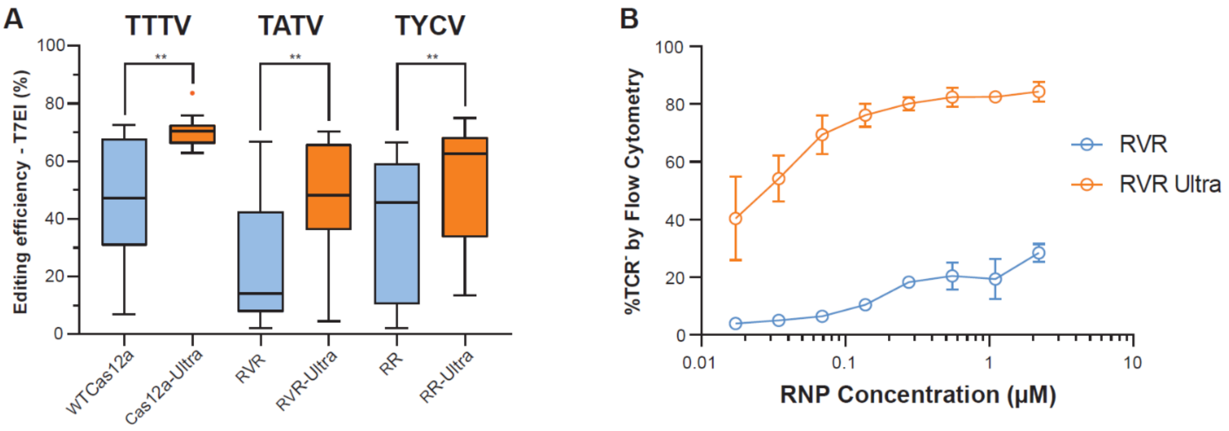


**Supplementary Fig. 4 Ultra mutations substantially increased the activity of AsCas12a-RR and RVR variants**. **a** Ultra mutations (M537R/F870L) were stacked on WT-, RR (S542R/K607R), and RVR (S542R/K548R/N552R) variants, and the editing efficiency of each variant was measured in HEK293 cells. Eleven crRNAs with optimal PAM sequence were tested for each variant (WT±Ultra: TTTV; RR±Ultra: TYCV; RVR±Ultra: TATV, n = 11 for each nuclease, 33 crRNAs in total). **b** Knock-out efficiency of RVR and RVR-Ultra over multiple doses in primary T cells with one crRNA (n = 1). Data are presented as mean values +/- SD. Paired, two-tailed t-test was performed to evaluate statistical significance (**: P-value = 0.0024, 0.0056, and 0.0029 respectively). Raw source data are provided in an online supplementary data file.

## Figure S5

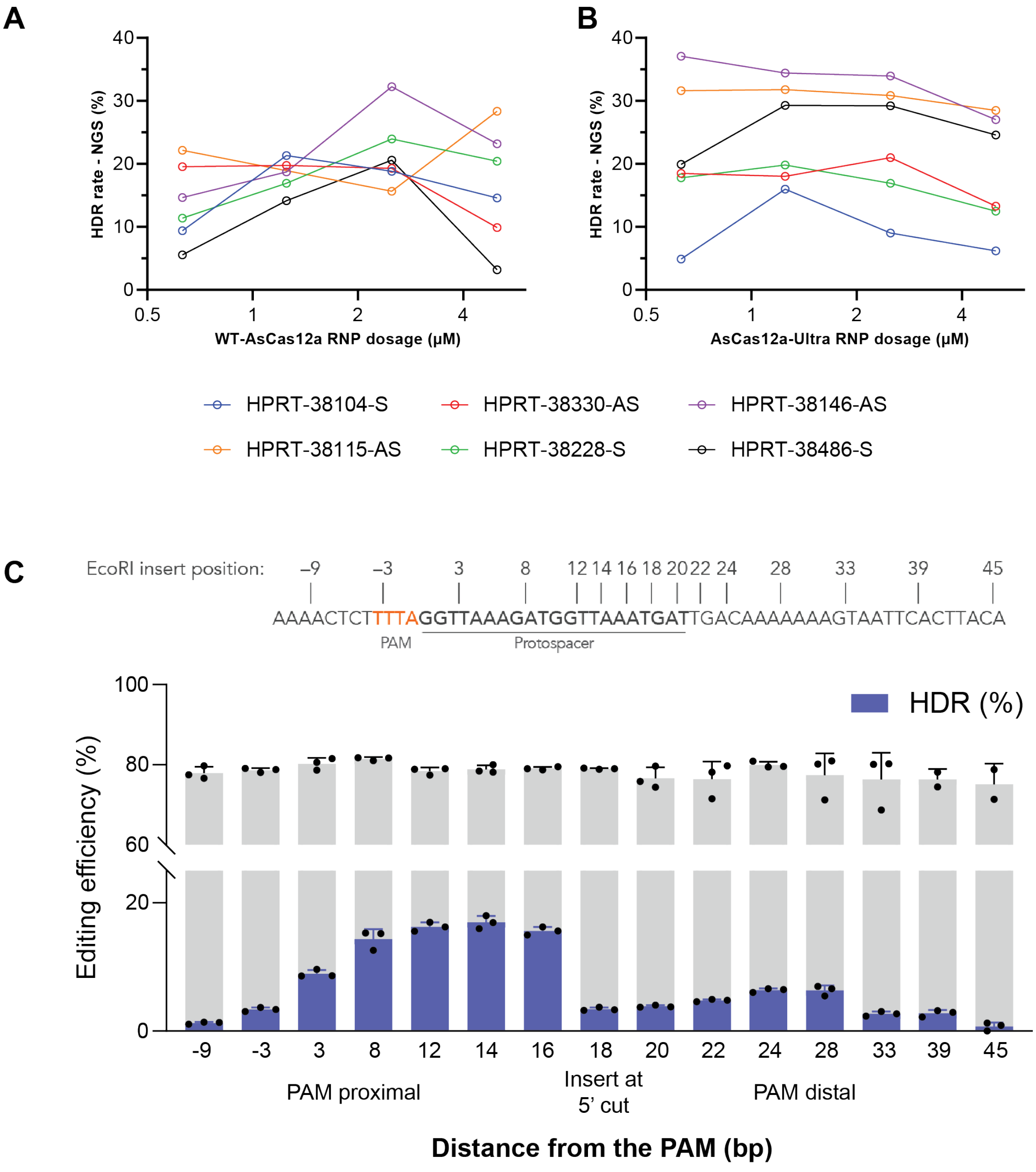


**Supplementary Fig. 5 Optimizing experimental conditions to achieve efficient HDR rate by AsCas12a-Ultra**. **a,b** Effect of RNP dosages of WT and Ultra on HDR rate. Assembled RNPs were titrated in the nucleofection reaction with fixed concentration of ssODN (3 μM) as donor template. Six pair of crRNA/ssODN were evaluated in human Jurkat cells. **c** Effect of insertion position within the target site of AsCas12a-Ultra on HDR efficiency. An EcoRI restriction sequence was inserted at various positions of the target site using ssODN (3 μM) as donor sequence. Ultra RNP was fixed at 2 μM in nucleofection reaction. Data are presented as mean values +/- SD. Raw source data are provided in an online supplementary data file.

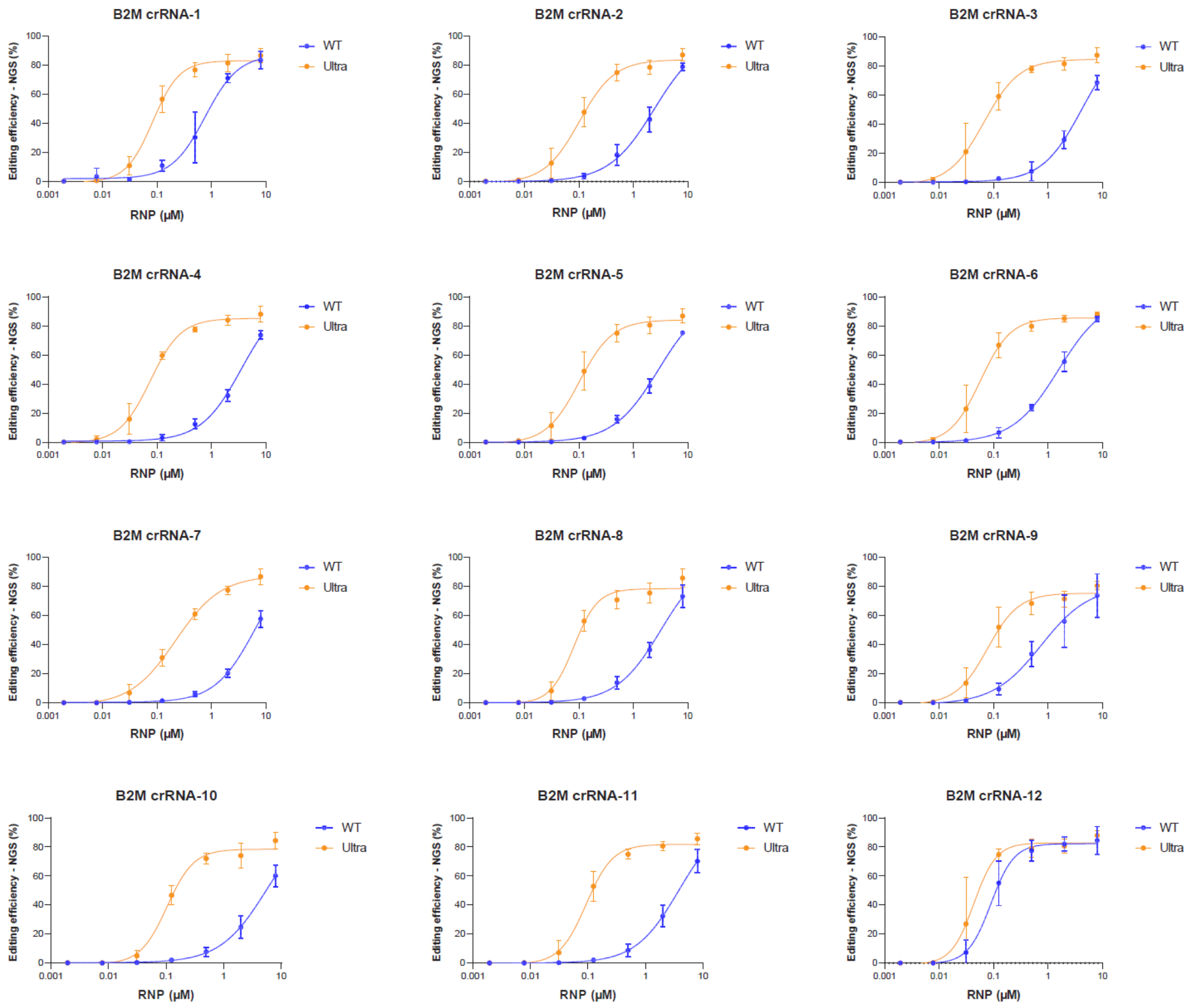


**Supplementary Fig. 6 In-depth activity comparison of AsCas12a-WT and Ultra in primary T cells targeting *B2M* locus**. The editing efficiency of WT and Ultra over a wide range of RNP doses at 12 crRNAs targeting *B2M* locus were measured by NGS 3-days post-electroporation. Dose-response curves were generated to estimate the EC50 of the editing, and summarized in Fig. 3c. Data are presented as mean values +/- SD. Raw source data are provided in an online supplementary data file.

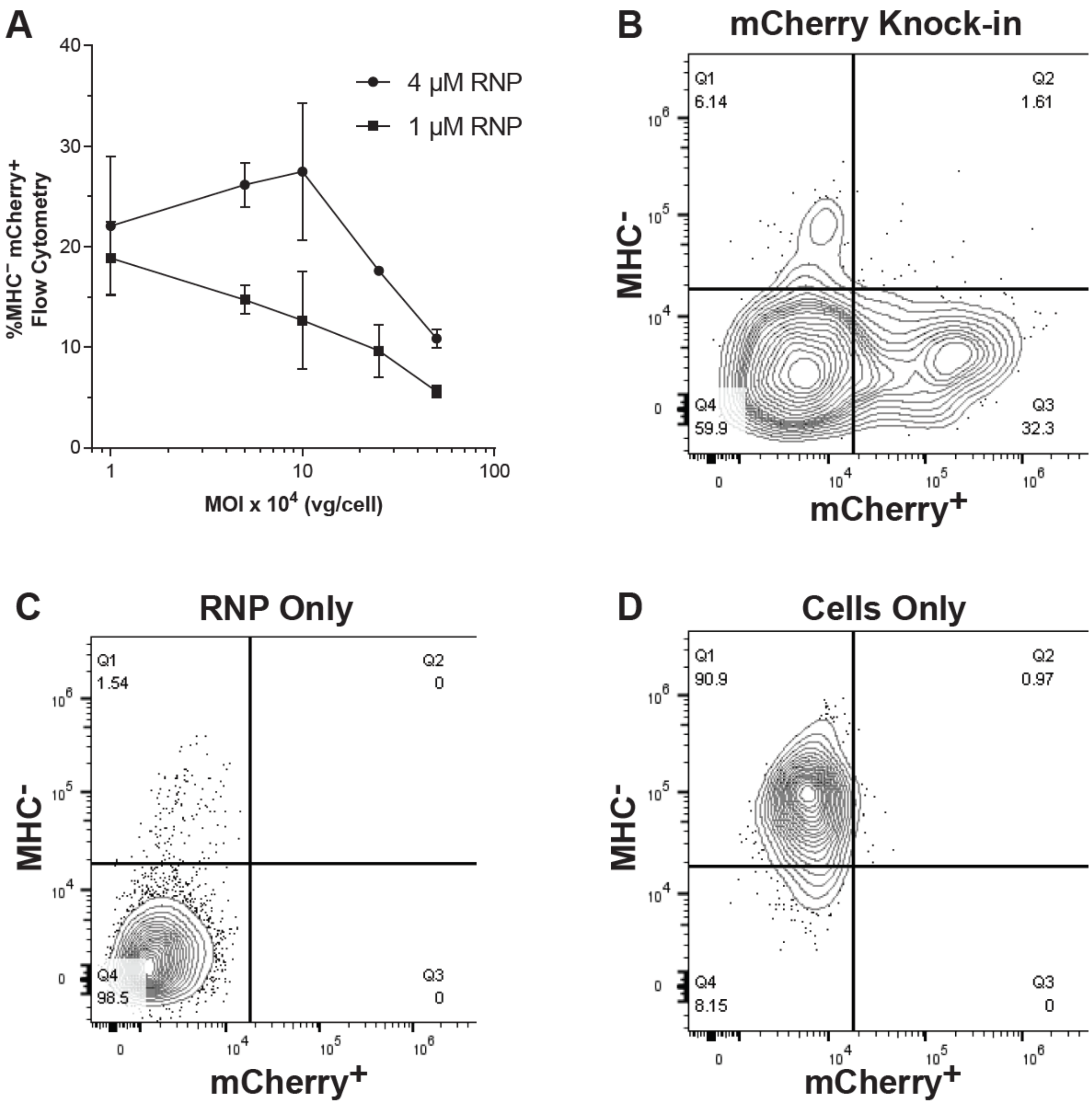


**Supplementary Fig. 7 Knock-in a transgene construct at *B2M* locus in HSCs by AsCas12a-Ultra using AAV6 as donor template. a** Knock-in efficiency of mCherry reporter transgene with variable MOI of AAV donor (1x$10^5$ vg/cell) at two RNP dosages. Data are presented as mean values +/- SD. **b-d** Representative result of flow cytometry to evaluate the knock-in efficiency of mCherry at *B2M* locus in NK cells with present of both AsCas12a RNP and AAV6 donor (b), or RNP (c) / Cell only (d). Raw source data are provided in an online supplementary data file.

## Figure S8

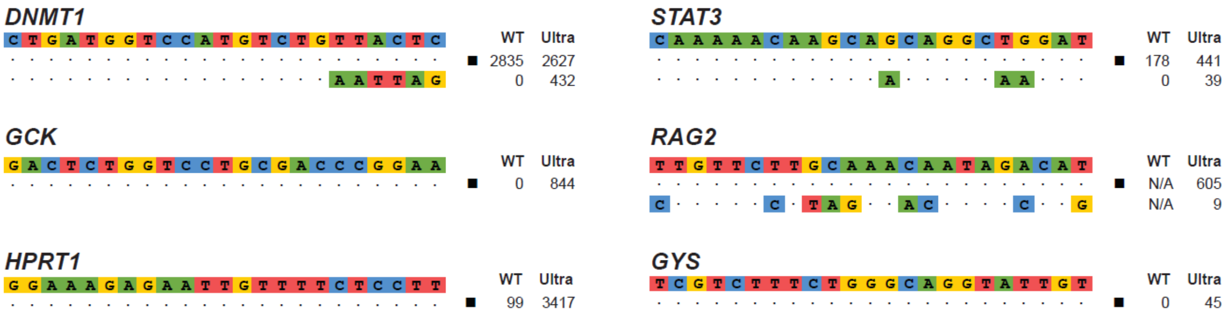


**Supplementary Fig. 8 Off-target activity of AsCas12a-WT and Ultra in HEK293 cells**. GUIDE-seq was performed for WT and Ultra over 6 crRNAs to detect any off-target tag integration. N/A: dataset not available.

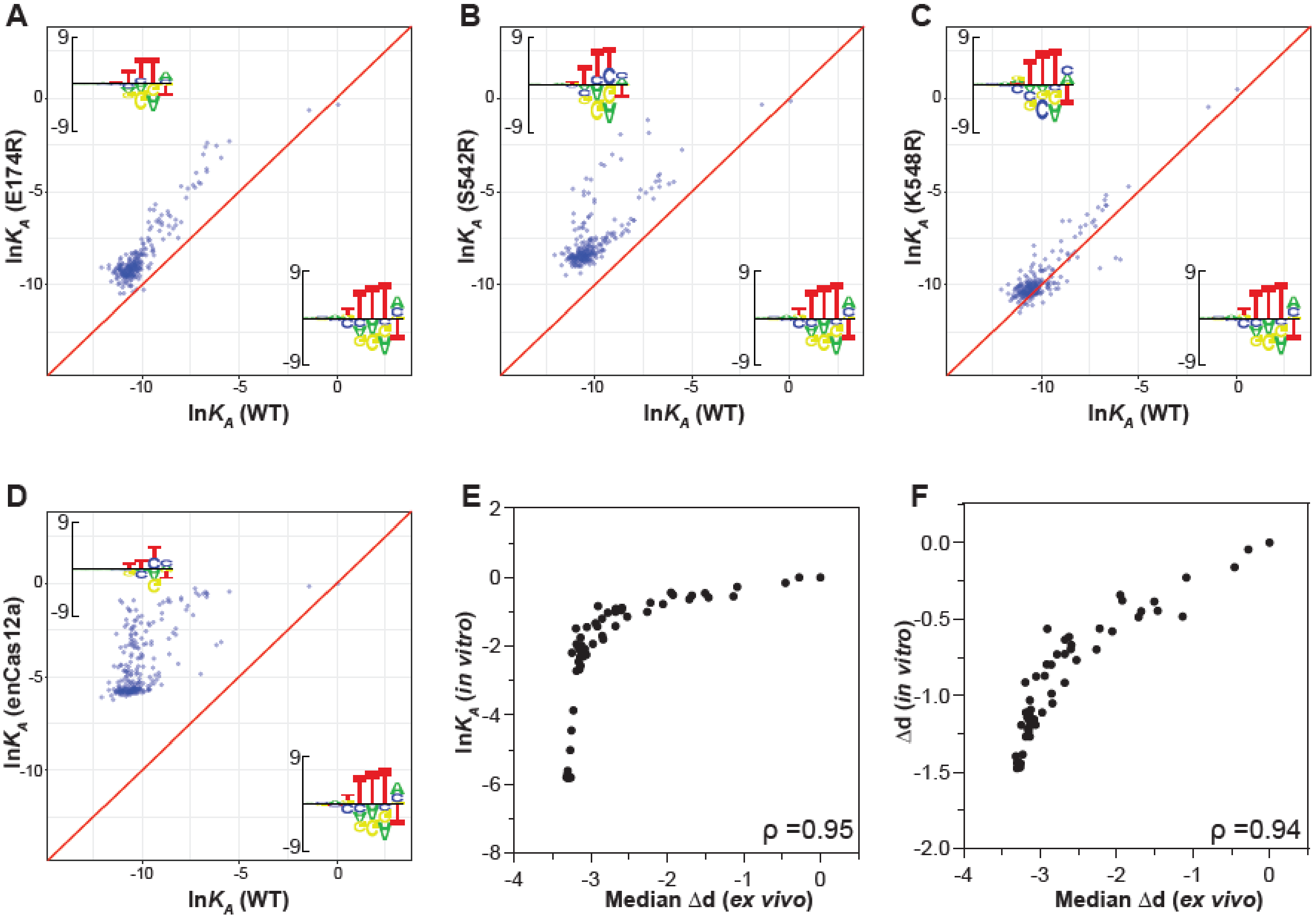


G

| Rank | WT | | E174R | | S542R | | K548R | | enCas12a | |
|---|---|---|---|---|---|---|---|---|---|---|
| | PAM | $\ln K_A$ | PAM | $\ln K_A$ | PAM | $\ln K_A$ | PAM | $\ln K_A$ | PAM | $\ln K_A$ |
| 1 | TTTC | 0.1 | TTTA | 0.0 | TTTA | 0.0 | TTTC | 0.5 | TTTC | 0.0 |
| 2 | TTTA | 0.0 | TTTC | -0.3 | TTTC | -0.1 | TTTA | 0.0 | TTTA | 0.0 |
| 3 | TTTG | -1.4 | TTTG | -0.6 | TTTG | -0.3 | TTTG | -0.5 | TTTG | -0.2 |
| 4 | TTTT | -5.5 | TTTT | -2.3 | TTCC | -1.2 | TTTT | -4.7 | TTCC | -0.3 |
| 5 | TCTC | -5.9 | CTTA | -2.4 | TTCA | -1.9 | ATTC | -5.2 | TTTT | -0.4 |
| 6 | TCTA | -6.2 | TCTA | -2.5 | TCCC | -1.9 | ATTA | -5.4 | ATTA | -0.5 |
| 7 | CTTA | -6.7 | ATTA | -2.6 | TTTT | -2.8 | CTTC | -5.7 | TTCA | -0.5 |
| 8 | ATTC | -6.7 | GTTA | -3.0 | TTCG | -3.0 | CTTA | -5.8 | ATTC | -0.5 |
| 9 | CTTC | -6.7 | TCTC | -3.2 | TTCT | -3.3 | GTTC | -5.8 | GTTA | -0.5 |
| 10 | ATTA | -6.7 | CTTC | -3.8 | TCCA | -3.5 | GTTA | -6.0 | CTTA | -0.6 |

**Supplementary Fig. 9 The expanded PAM flexibility of enCas12a is mediated by S542R. a-d** The relative DNA binding affinities of E174R (a), S542R (b), K548R (c), and enCas12a (d) over all 256 PAM variants *in vitro*. Upper left: position-specific affinity matrix logo for each AsCas12a variant over PAM region. Bottom right: logo for WT-AsCas12a. Although all 3 point mutations globally enhanced the non-specific DNA binding affinity, only S542R significantly expanded the PAM repertoire of AsCas12a over sequences with cytosine at 3$^{rd}$ position of the PAM region. **e,f** PAM preference of enCas12a defined by *in vitro* approach (this study) and lentivirus-based *ex vivo* approach over 57 PAM sequences covered by both studies. Normalized depletion of each crRNA targeting essential genes in the lentivirus-based tiling assay was calculated. The natural logarithm of median depletion for each PAM sequence was plotted against the relative binding affinity (e), and cleavage specificity (f) determined *in vitro*. Highly consistent PAM preference of enCas12a was defined by two distinct approaches, as judged by the Spearman's Rho. (g) The top 10 PAM sequences targeted by each nuclease based on the *in vitro* DNA binding specificity. Raw source data are provided in an online supplementary data file.

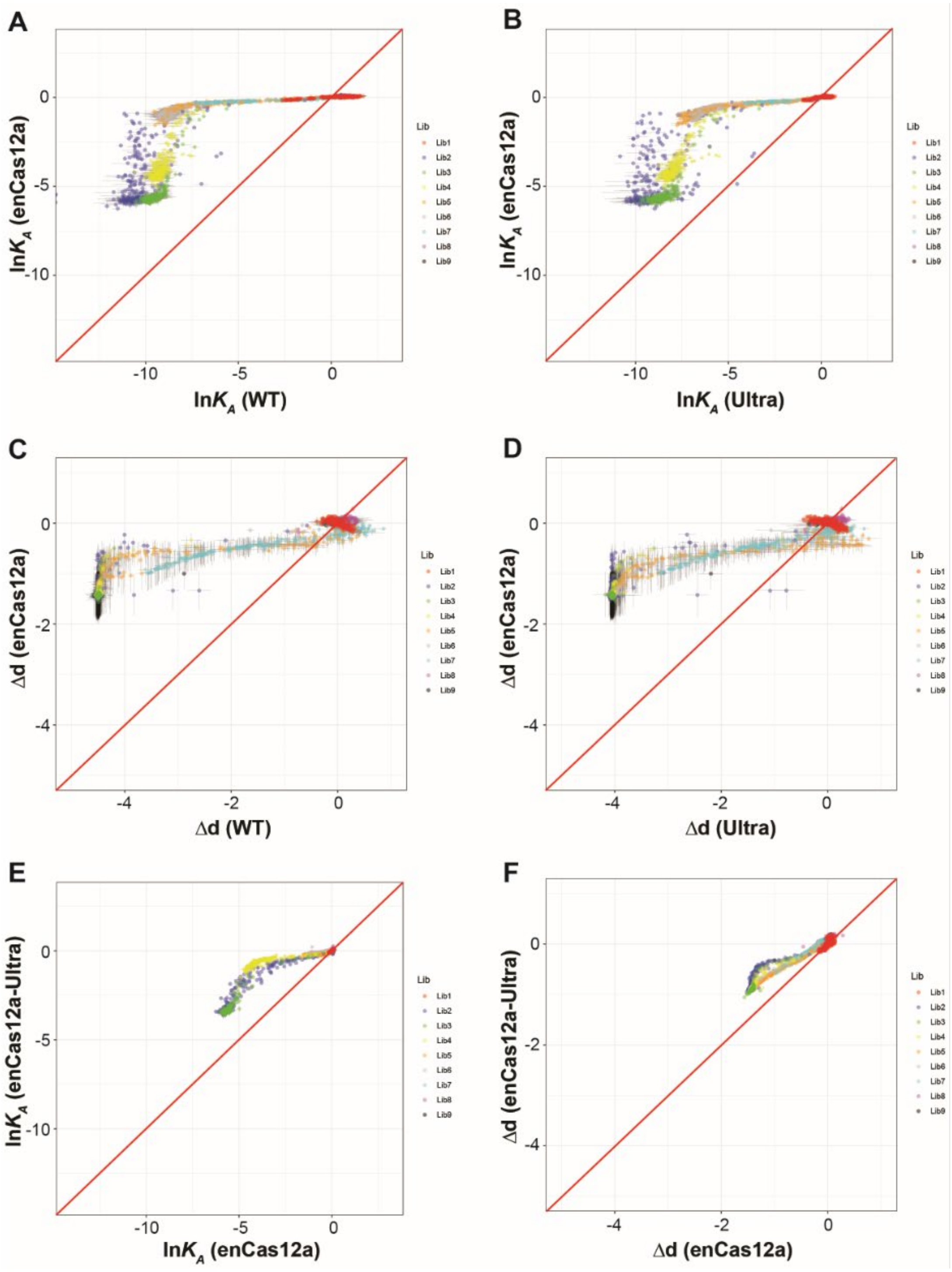


**Supplementary Fig. 10 Evaluating the intrinsic DNA binding and cleavage specificities of enCas12a and enCas12a-Ultra. a,b** Comparison of relative DNA binding affinity of WT (a) or Ultra (b) versus enCas12a. **c,d** Same as (a,b) but for relative DNA cleavage activity. **e,f** Comparison of relative DNA binding and cleavage between enCas12a and enCas12a-Ultra. Data are presented as mean values +/- SD.

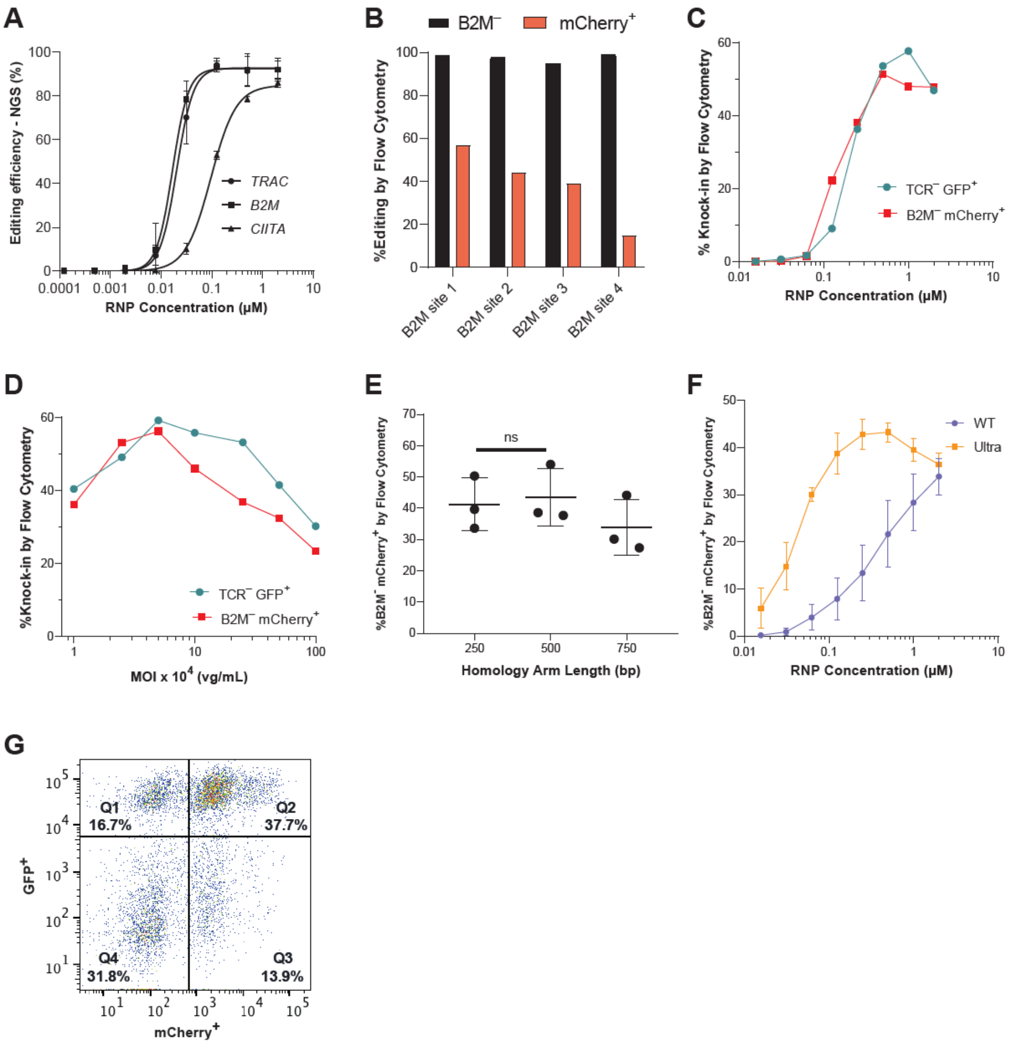


**Supplementary Fig. 11 Optimizing experimental conditions to achieve efficient HDR rate by AsCas12a-Ultra using AAV6 as HDR donor template in primary T cells**. **a** Editing efficiency of Ultra at *TRAC*, *B2M*, *CIITA* locus over a range of RNP dose. Note: these specific crRNAs were used for the knock-in experiments. Data are presented as mean values +/- SD. **b** Knock-in efficiency of mCherry transgene using Ultra at 4 target sites in *B2M* locus. **c** Knock-in efficiency of fluorescent reporter transgenes at each genomic locus over a range of RNP dose with fixed MOI of AAV donor (1x10$^5$ vg/cell). **d** Knock-in efficiency of a fluorescent reporter at either *TRAC* or *B2M* locus across a range of MOIs. RNP dose was fixed at 1 μM. **e** Knock-in efficiency of mCherry at *B2M* locus with using donor templates with different length of homology arm (RNP:1 μM; AAV MOI: 1x10$^5$ vg/cell). Data are presented as mean values +/- SD. One-way ANOVA was performed to evaluate statistical significance (ns: P-value > 0.05). **f** Knock-in efficiency of mCherry at *B2M* locus using WT or Ultra over a range of RNP dose. Data are presented as mean values +/- SD. **g** Representative result of flow cytometry for simultaneous double knock-in of fluorescent reporters at both *TRAC* and *B2M* loci by Ultra (RNP:1 μM; AAV MOI: 1x10$^5$ vg/cell). Raw source data are provided in an online supplementary data file.

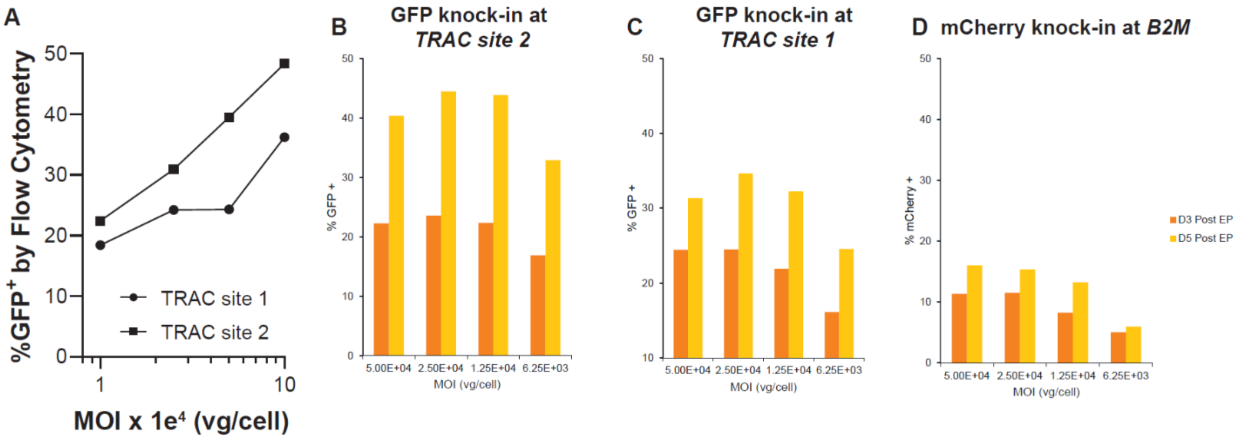


**Supplementary Fig. 12 Additional optimization of knock-in condition to generate CAR-NK cells. a** Transgene knock-in by AsCas12a-Ultra at two RNPs doses using variable MOI of AAV donor template. **b-d** Assessment of HDR rate by flow cytometry 3 or 5-days post-delivery over three target loci.

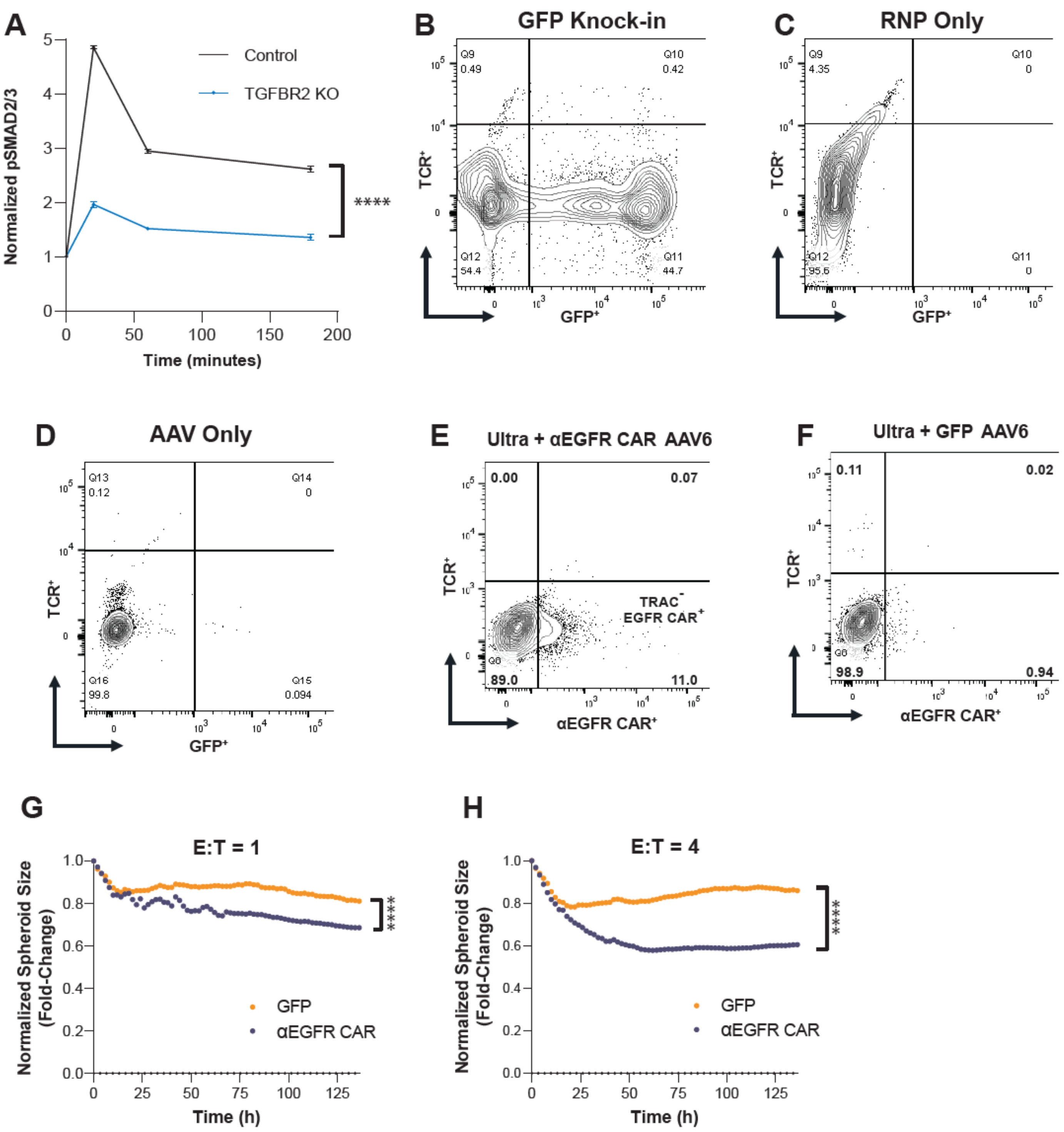
A
Normalized pSMAD2/3
Time (minutes)
Control
TGFBR2 KO
****
B
GFP Knock-in
TCR+
GFP+
Q9 0.49
Q10 0.42
Q12 54.4
Q11 44.7
C
RNP Only
Q9 4.35
Q10 0
Q12 95.6
Q11 0
D
AAV Only
Q13 0.12
Q14 0
Q16 99.8
Q15 0.094
E
Ultra + αEGFR CAR AAV6
0.00
0.07
TRAC-
EGFR CAR+
89.0
11.0
αEGFR CAR+
F
Ultra + GFP AAV6
0.11
0.02
98.9
0.94
G
E:T = 1
Normalized Spheroid Size (Fold-Change)
Time (h)
GFP
αEGFR CAR
H
E:T = 4

**Supplementary Fig. 13 Additional information to generate CAR-NK cells. a** Phospho flow cytometry of *TGFBR2* knock-out NK cells as compared to control unedited NK cells. NK cells were cultured and edited as per SOP. 72 hours post editing, NK cells were stimulated with 10ng/mL TGF-β for indicated time, fixed and stained for pSMAD2/3 prior to interrogation by flow cytometry. Data are representative of 3 independent experiments with 4 unique NK cell donors. Error bars represent standard deviation of 3 technical replicates. Data are presented as mean values +/- SD. **b** Flow cytometry plot of GFP knock-in with AAV6 using $2.5x10^4$ vg/cell MOI in NK cells. Flow cytometry plots of RNP only and AAV only controls are shown in **c** and **d**, respectively. Additional information of CAR-NK cells generated by Ultra. **e** Representative result of flow cytometry to evaluate the knock-in efficiency of *EGFR* CAR at *TRAC* locus in NK cells. **f** Same as in (a), but using an AAV6 carrying GFP transgene as negative control to evaluate the specificity of EGFR antibody used in flow analysis. **g,h** Tumor killing assay of CAR-NK at effector-to-target cells ratios (E:T) of 1 and 4, respectively. Two-way ANOVA was performed to evaluate statistical significance (****: P-value ≤ 0.0001). Raw source data are provided in an online supplementary data file.

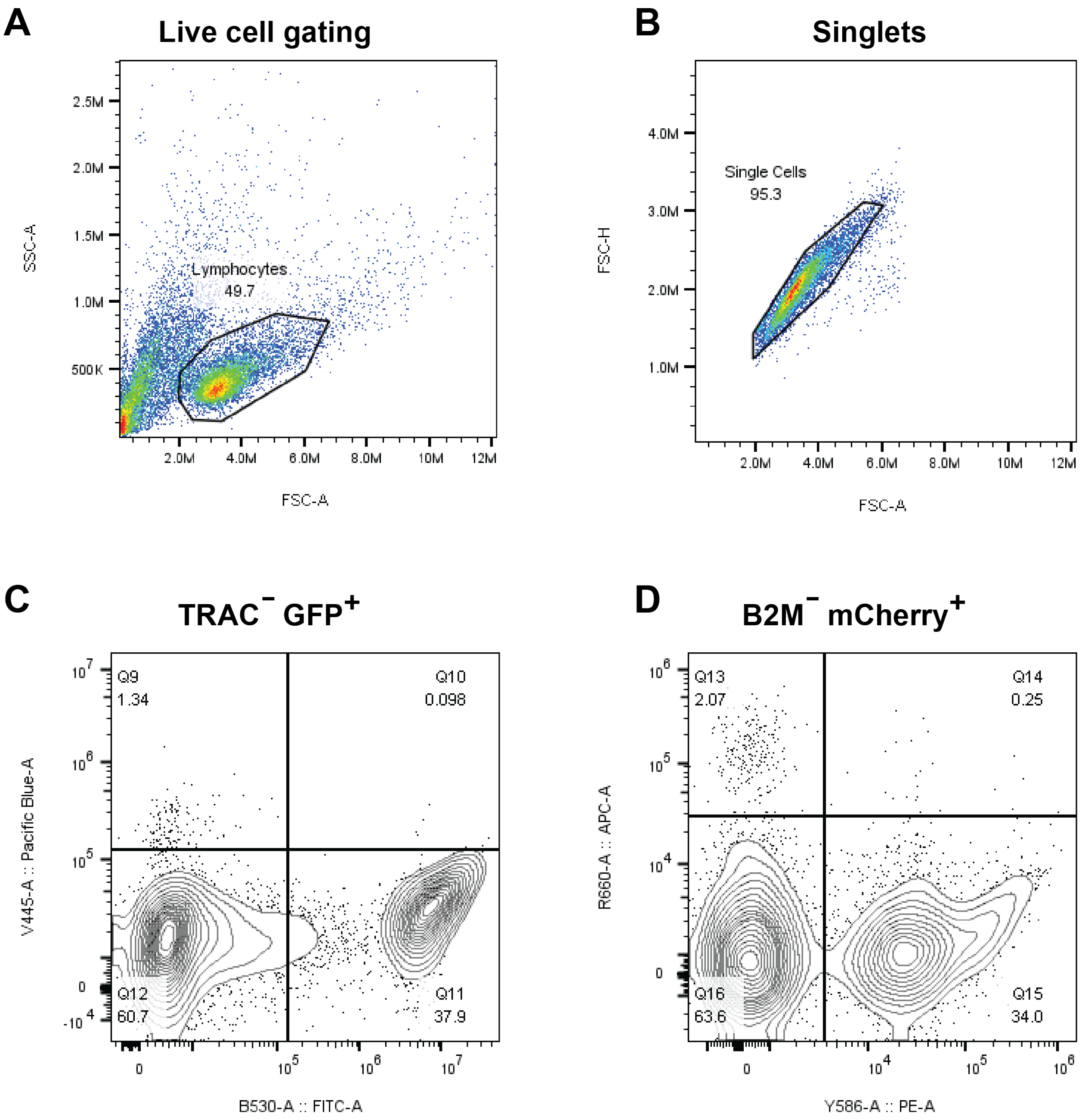


**Supplementary Fig. 14 Flow cytometry gating strategy for mCherry and GFP knock-in at TRAC and B2M locus in primary T cells. a** Gating strategy for live cells and **b** singlets. **c** Flow cytometry analysis of *TRAC⁻/GFP⁺* or **d** *B2M⁻/mCherry⁺* cells.

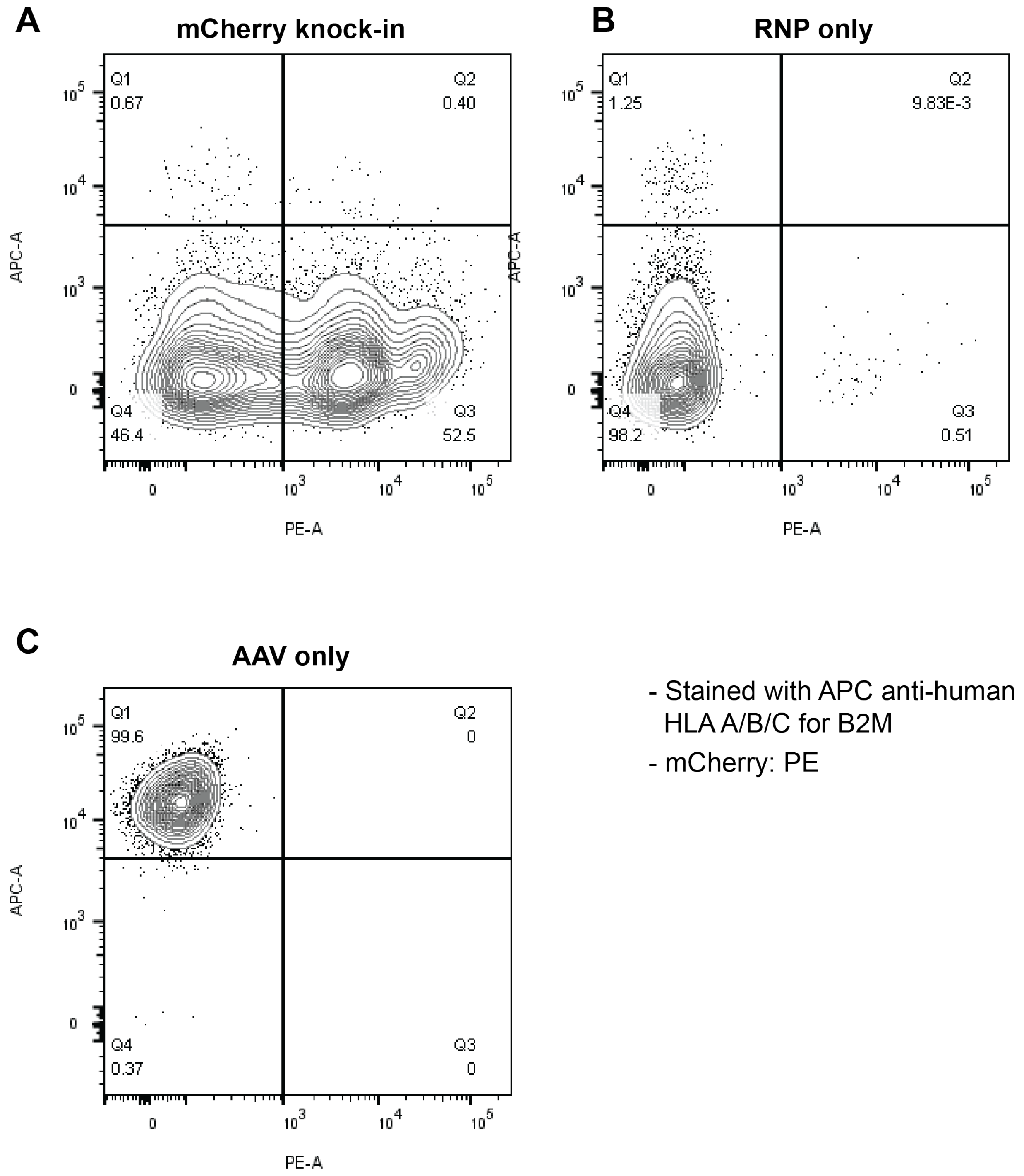


**Supplementary Fig. 15 Flow cytometry gating strategy for mCherry knock-in at B2M locus in primary T cells. a** Flow cytometry results when both RNP and AAV donor are used or **b,c** when only RNP / AAV is used in the electroporation.

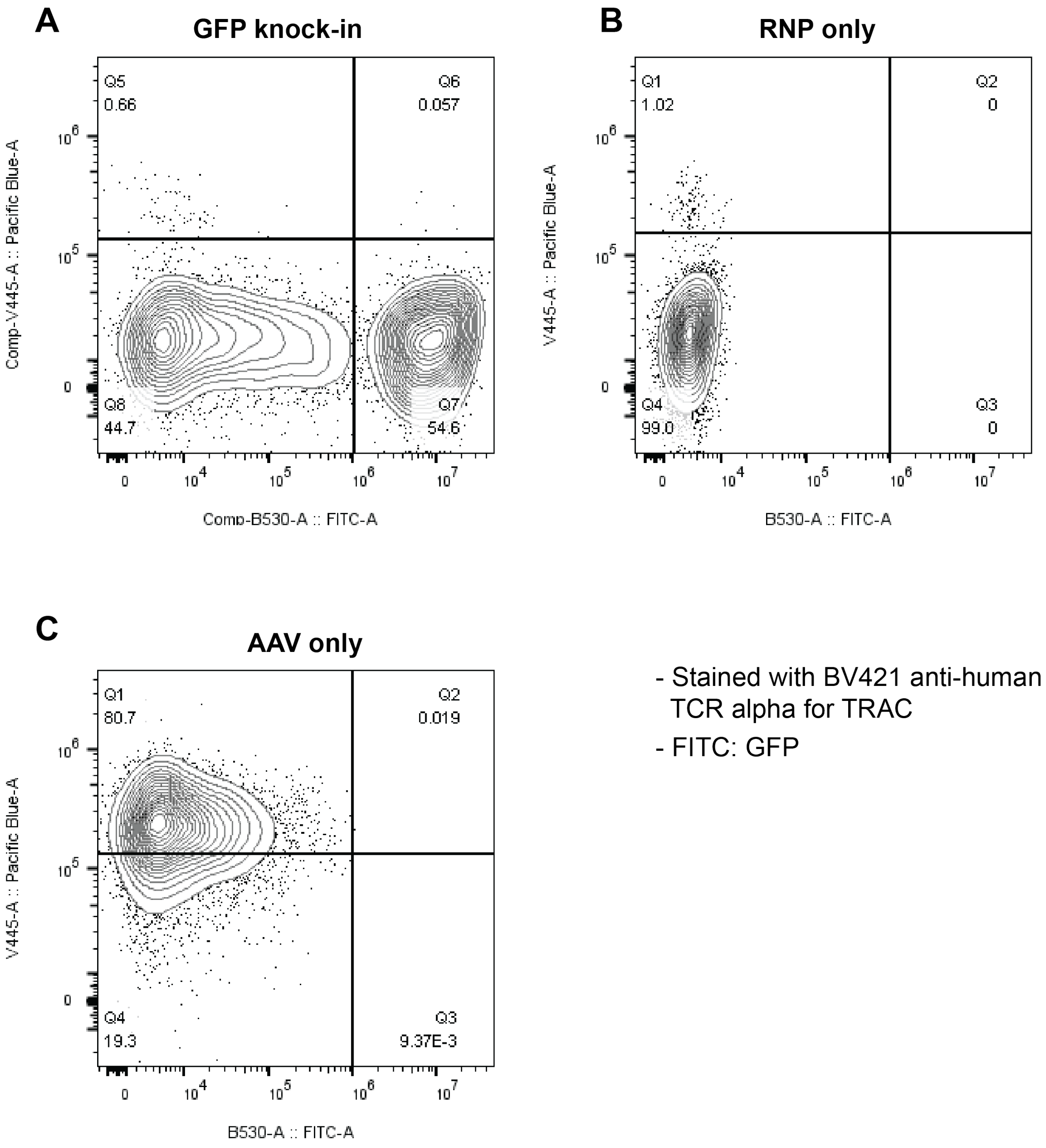


**Supplementary Fig. 16 Flow cytometry gating strategy for GFP knock-in at B2M locus in primary T cells. a** Flow cytometry results when both RNP and AAV donor are used, or **b,c** when only RNP / AAV is used in the electroporation.

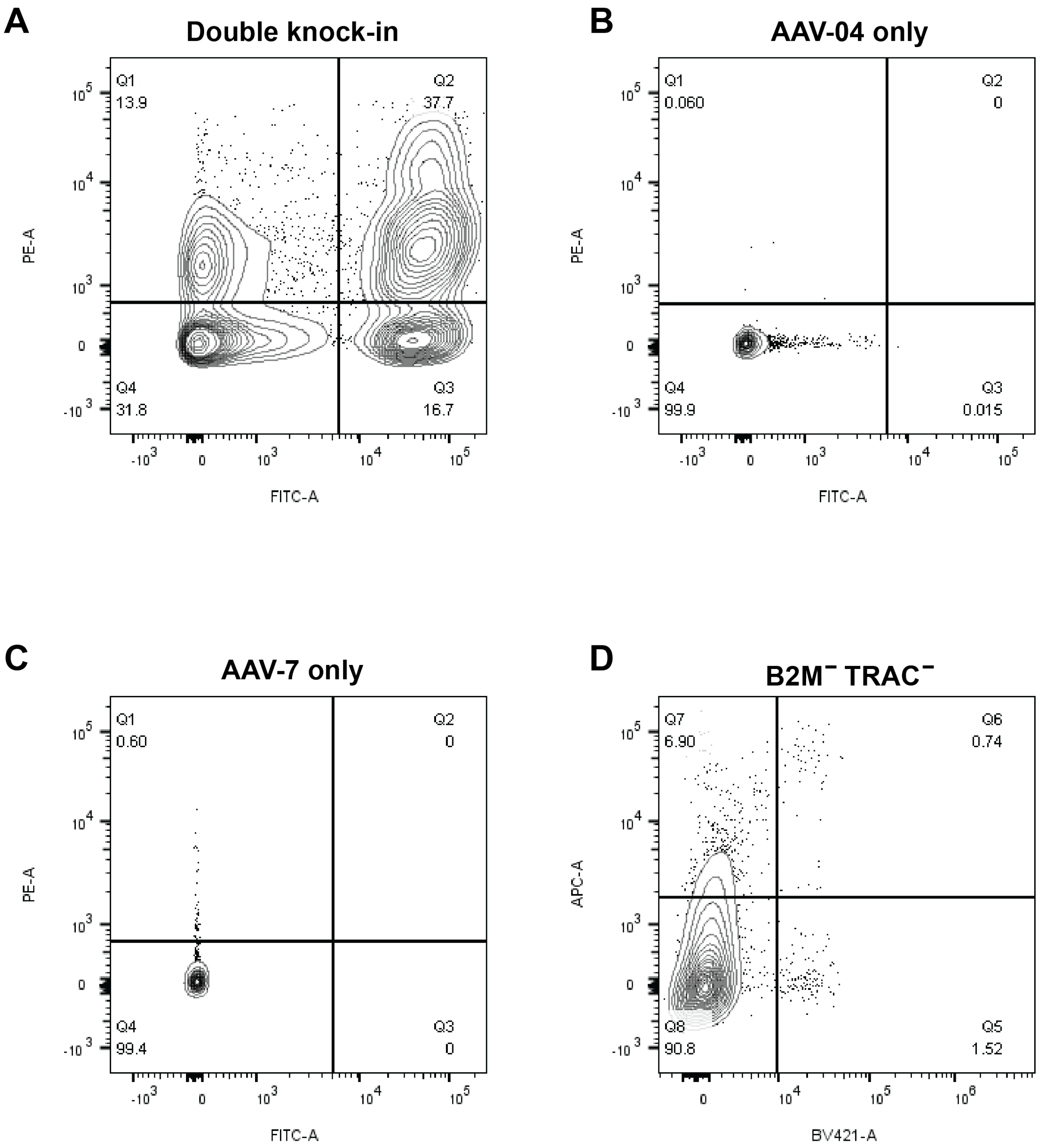


- Stained with APC anti-human HLA A/B/C for B2M
- Stained with BV421 anti-human TCR alpha for TRAC
- mCherry: PE;  GFP: FITC

**Supplementary Fig. 16 Flow cytometry gating strategy for GFP/mCherry double knock-in at B2M and TRAC loci in primary T cells.** **a** Flow cytometry analysis of GFP / mCherry expression when both RNPs and AAV donors are used, or **b,c** when AAV template alone is used in the electroporation. **d** Flow cytometry analysis for B2M and TRAC expression when both RNPs and AAV donors are used.

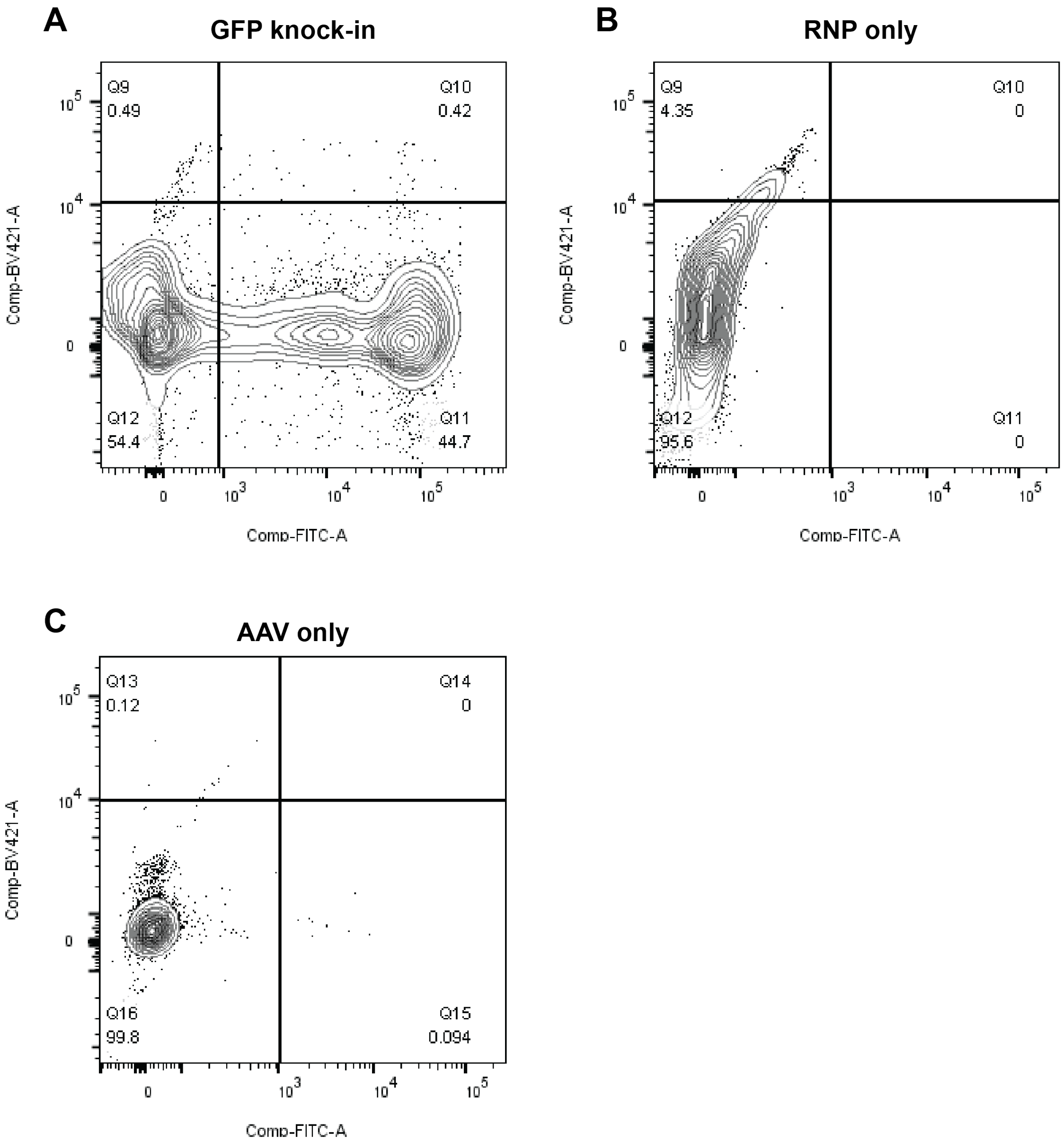


- Stained with BV421 anti-human TCR alpha for TRAC
- GFP: FITC

**Supplementary Fig. 17 Flow cytometry gating strategy for GFP knock-in at TRAC locus in primary NK cells. a** Flow cytometry analysis of GFP / TRAC expression when both RNPs and AAV donors are used, or **b,c** when either AAV template alone is used in the electroporation.

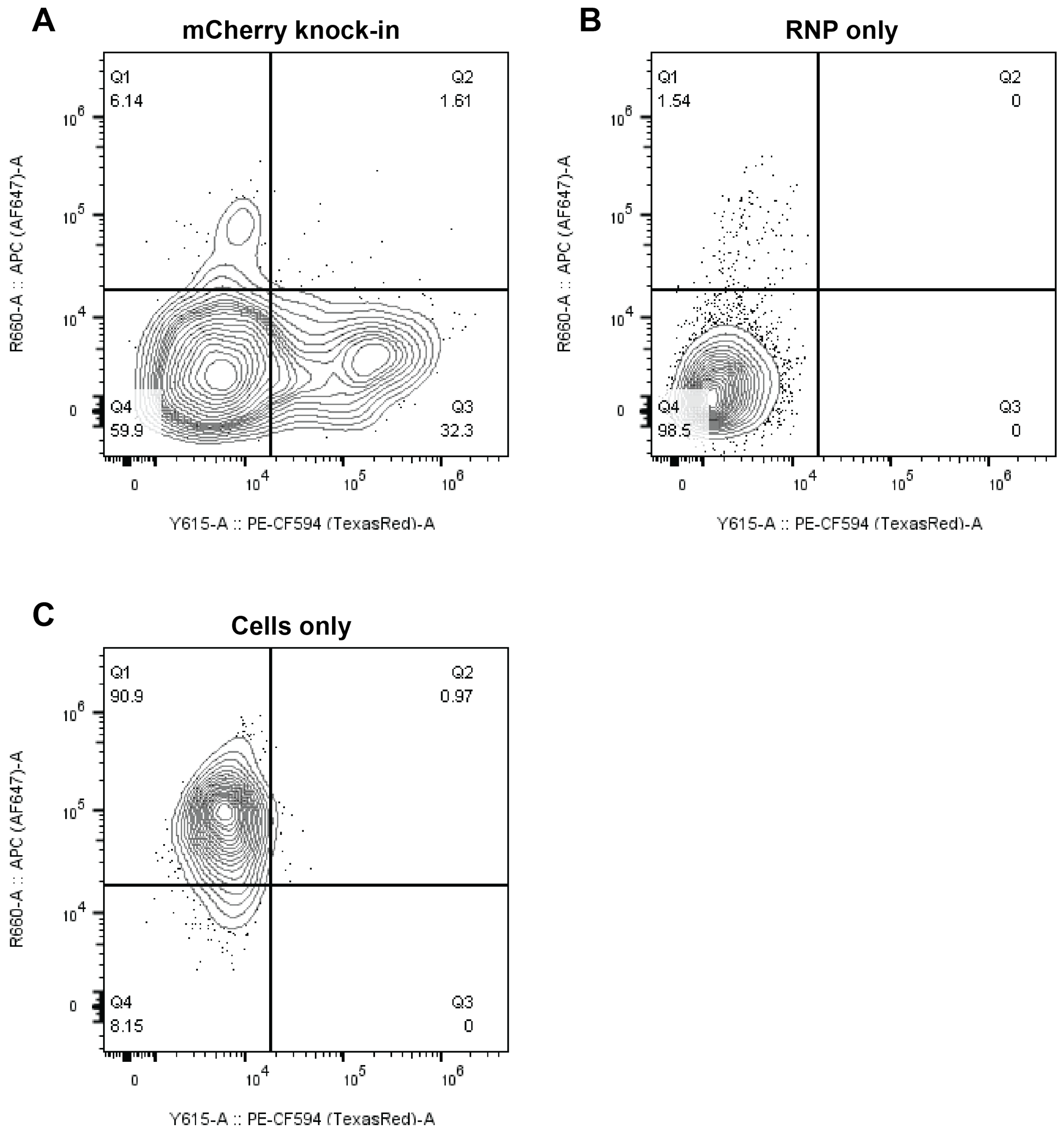


- Stained with APC anti-human HLA A/B/C for B2M
- mCherry: PE

**Supplementary Fig. 18 Flow cytometry gating strategy for mCherry knock-in at B2M locus in HSCs. a** Flow cytometry analysis of mCherry / B2M expression when both RNPs and AAV donors, or **b** only RNP is used**. c** Expression measurement of HSC cells without RNP and AAV donor.

| | |
|---|---|
| **AsCas12a gene sequence for bacterial screen** | |
| atgggcaagcccatccctaacccccctgttggggctggacagcaccgctcccaaaaagaaaaggaaggtgggcattcacggcgtgcctgcggccACCCAGTTCGAGGGCTTCACCAACCTGTACCAGGTGTCC | |
| AAGACCCTGAGATTCGAGCTGATCCCCCAGGGCAAGACACTGAAGCACATCCAGGAACAGGGCTTCATCGAAGAGGACAAGGCCCGGAACGACCACTACAAAGAGCTGAAGCCCATCATCGACCGGATCTAC | |
| AAGACCTACGCCGACCAGTGCCTGCAGCTGGTGCAGCTGGACTGGGAGAATCTGAGCGCCGCCATCGACAGCTACCGGAAAGAGAAAACCGAGGAAACCCGGAACGCCCTGATCGAGGAACAGGCCACCTAC | |
| AGAAACGCCATCCACGACTACTTCATCGGCCGGACCGACAACCTGACCGACGCCATCAACAAGCGGCACGCCGAGATCTATAAGGGCCTGTTCAAGGCCGAGCTGTTCAACGGCAAGGTGCTGAAGCAGCTG | |
| GGCACCGTGACCACCACCGAGCACGAAAACGCCCTGCTGCGGAGCTTCGACAAGTTCACCACCTACTTCAGCGGCTTCTACGAGAACCGGAAGAACGTGTTCAGCGCCGAGGACATCAGCACCGCCATCCCC | |
| CACAGAATCGTGCAGGACAACTTCCCCAAGTTCAAAGAGAACTGCCACATCTTCACCCGGCTGATCACCGCCGTGCCCAGCCTGAGAGAACACTTCGAGAACGTGAAGAAGGCCATCGGCATCTTCGTGTCC | |
| ACCAGCATCGAGGAAGTGTTCAGCTTCCCATTCTACAACCAGCTGCTGACCCAGACCCAGATCGACCTGTATAATCAGCTGCTGGGCGGCATCAGCAGAGAGGCCGGCACCGAGAAGATCAAGGGCCTGAAC | |
| GAAGTGCTGAACCTGGCCATCCAGAAGAACGACGAGACAGCCCACATCATTGCCAGCCTGCCCCACCGGTTCATCCCTCTGTTCAAGCAGATCCTGAGCGACAGAAACACCCTGAGCTTCATCCTGGAAGAG | |
| TTCAAGTCCGATGAGGAAGTGATCCAGAGCTTCTGCAAGTATAAGACCCTGCTGAGGAACGAGAATGTGCTGGAAACCGCCGAGGCCCTGTTCAATGAGCTGAACAGCATCGACCTGACCCACATCTTTATC | |
| AGCCACAAGAAGCTGGAAACAATCAGCAGCGCCCTGTGCGACCACTGGGACACACTGCGGAATGCCCTGTACGAGCGGCGGATCTCTGAGCTGACCGGCAAGATCACCAAGAGCGCCAAAGAAAAGGTGCAG | |
| CGGAGCCTGAAGCACGAGGATATCAACCTGCAGGAAATCATCAGCGCCGCTGGCAAAGAACTGAGCGAGGCCTTTAAGCAGAAAACCAGCGAGATCCTGTCCCACGCCCACGCCGCACTGGATCAGCCTCTG | |
| CCTACCACCCTGAAGAAGCAGGAAGAGAAAGAGATCCTGAAGTCCCAGCTGGACAGCCTGCTGGGCCTGTACCATCTGCTGGATTGGTTCGCCGTGGACGAGAGCAACGAGGTGGACCCCGAGTTCTCCGCC | |
| AGACTGACAGGCATCAAACTGGAAATGGAACCCAGCCTGTCCTTCTACAACAAGGCCAGAAACTACGCCACCAAGAAACCCTACAGCGTGGAAAAGTTTAAGCTGAACTTCCAGATGCCCACCCTGGCCAGC | |
| GGCTGGGACGTGAACAAAGAGAAGAACAACGGCGCCATCCTGTTCGTGAAGAACGGACTGTACTACCTGGGCATCATGCCTAAGCAGAAGGGCAGATACAAGGCCCTGTCCTTTGAGCCCACCGAAAAGACC | |
| AGCGAGGGCTTTGACAAGATGTACTACGATTACTTCCCCGACGCCGCCAAGATGATCCCCAAGTGCAGCACCCAGCTGAAGGCCGTGACCGCCCACTTTCAGACCCACACCACCCCCATCCTGCTGAGCAAC | |
| AACTTCATCGAGCCCCTGGAAATCACCAAAGAGATCTACGACCTGAACAACCCCGAGAAAGAGCCCAAGAAGTTCCAGACCGCCTACGCCAAGAAAACCGGCGACCAGAAGGGCTACCGCGAGGCTCTGTGC | |
| AAGTGGATCGACTTTACCCGGGACTTCCTGAGCAAGTACACCAAGACCACCTCCATCGATCTGAGCAGCCTGCGGCCCAGCTCCCAGTACAAGGATCTGGGCGAGTACTACGCCGAGCTGAACCCTCTGCTG | |
| TACCACATCAGCTTCCAGCGGATCGCCGAAAAAGAAATCATGGACGCCGTGGAAACCGGCAAGCTGTACCTGTTCCAGATCTATAACAAGGACTTCGCCAAGGGCCACCACGGCAAGCCCAATCTGCACACC | |
| CTGTACTGGACCGGCCTGTTTAGCCCCGAGAATCTGGCCAAGACCAGCATCAAGCTGAACGGCCAGGCCGAACTGTTTTACCGGCCCAAGAGCCGGATGAAGCGGATGGCCCATAGACTGGGCGAGAAGATG | |
| CTGAACAAGAAACTGAAGGACCAGAAAACCCCTATCCCCGACACACTGTATCAGGAACTGTACGACTACGTGAACCACCGGCTGAGCCACGACCTGTCCGACGAAGCTAGAGCACTGCTGCCCAACGTGATC | |
| ACAAAAGAGGTGTCCCACGAGATCATCAAGGACCGGCGGTTTACCTCCGATAAGTTCTTCTTCCACGTGCCCATCACCCTGAACTACCAGGCCGCCAACAGCCCCAGCAAGTTCAACCAGAGAGTGAACGCC | |
| TACCTGAAAGAGCACCCCGAGACACCCATCATTGGCATCGACAGAGGCGAGCGGAACCTGATCTACATCACCGTGATCGACAGCACAGGCAAAATCCTGGAACAGAGAAGCCTGAACACCATCCAGCAGTTC | |
| GACTACCAGAAGAAACTGGACAACCGGGAAAAAGAACGGGTGGCCGCCAGACAGGCTTGGAGCGTCGTGGGCACCATTAAGGACCTGAAGCAGGGCTACCTGAGCCAAGTGATTCACGAGATCGTGGACCTG | |
| ATGATCCACTATCAGGCTGTGGTGGTGCTGGAAAACCTGAACTTCGGCTTCAAGAGCAAGCGGACCGGAATCGCCGAGAAAGCCGTGTACCAGCAGTTTGAGAAAATGCTGATCGACAAGCTGAATTGCCTG | |
| GTGCTGAAAGACTACCCCGCTGAGAAAGTGGGAGGCGTGCTGAATCCCTACCAGCTGACCGACCAGTTCACCTCCTTTGCCAAGATGGGAACCCAGAGCGGCTTCCTGTTCTACGTGCCAGCCCCCTACACC | |
| AGCAAGATCGACCCTCTGACCGGCTTCGTGGACCCCTTCGTGTGGAAAACCATCAAGAACCACGAGTCCCGGAAGCACTTCCTGGAAGGCTTTGACTTCCTGCACTACGACGTGAAAACAGGCGATTTCATC | |
| CTGCACTTCAAGATGAATCGGAATCTGTCCTTCCAGAGGGGCCTGCCCGGCTTCATGCCTGCCTGGGATATCGTGTTCGAGAAGAATGAGACACAGTTCGACGCCAAGGGAACCCCCTTTATCGCCGGCAAG | |
| AGGATCGTGCCTGTGATCGAGAACCACAGATTCACCGGCAGATACCGGGACCTGTACCCCGCCAACGAGCTGATTGCCCTGCTGGAAGAGAAGGGCATCGTGTTCCGGGACGGCAGCAACATCCTGCCCAAG | |
| CTGCTGGAAAATGACGACAGCCACGCCATCGATACCATGGTGGCACTGATCCGCAGCGTGCTGCAGATGCGGAACAGCAATGCCGCCACCGGCGAGGACTACATCAATAGCCCAGTGCGGGACCTGAACGGC | |
| GTGTGCTTCGACAGCAGATTCCAGAACCCCGAGTGGCCCATGGATGCCGACGCCAATGGCGCCTACCACATTGCCCTGAAGGGACAGCTGCTGCTGAACCATCTGAAAGAGAGCAAAGACCTGAAACTGCAG | |
| AACGGCATCTCCAACCAGGACTGGCTGGCCTATATCCAGGAACTGCGGAACcccaaaaagaaaaggaaggtg | |
| **Primers for Random mutagenesis** | |
| Cpf1_PID_mut_fwd | ATCAAACTGGAAATGGAACCCAGCCTGTCCTTCTACAACAAGGCCAGAAACTACGCCACC |
| Cpf1_PID_mut_REV | AATGATGGGTGTCTCGGGGTGCTCTTTCAGGTAGGCGTTCACTCTCTGGTTGAACTTGC |
| **Primers for vector linearization** | |
| Cpf1_VecBB_PID_rev | GGTGGCGTAGTTTCTGGCCTTGTTGTAGAAGGACAGGCTGGGTTCCATTTCCAGTTTGAT |
| Cpf1_VecBB_PID_fwd | AGCAAGTTCAACCAGAGAGTGAACGCCTACCTGAAAGAGCACCCCGAGACACCCATCATT |
| **PCR primers to amplify mutagenized region for Nextera sequencing** | |
| Cpf1_PID_nextera_F | CCTCTGCCTACCACCCTGAAG |
| Cpf1_PID_nextera_R | AGGTAGCCCTGCTTCAGGTC |
| **crRNA for bacterial selection** | |
| ACATAAAACTCTTTTAGGTTA | |

**Supplementary Table 1 DNA sequences used in the bacterial selection of AsCas12a Ultra.**

| | |
|---|---|
| **gBlock sequence for indivudal in-vitro clevage assay** | |
| HPRT-38346-TTTT | CTCGGCCCAGGCTGGCCATTCCGGATGATTCTCCTACTTCCTCCTCGGAGTCCTCAAGTATCACTGTCCCCTTGCACTCCCTGTCTTTGAATCTTACTTTCCATCCTTTTTACATAAAACTC<br>TTTTAGGTTACCAAGCTGGACCCCACGAAGTGTTGGATATAAGAAAAACTCTGGAGAGCAGTGAGCCCAGCAGAGCCCCCCTAGGGATCTTCAGCCCCAGCCTGAAGTATTCATTATAGTCA<br>AAAAAGGCTGTGGTTTCACAGGTGCAGGTCCAACCCTCCTTTCTCTGCCTTGTGGCTGCCTGTCCTGGGGCAGGTTACGTTGCCCCTGGGAGCCTCTGTTTTCTGTAA |
| HPRT-38346-TTTC | CTCGGCCCAGGCTGGCCATTCCGGATGATTCTCCTACTTCCTCCTCGGAGTCCTCAAGTATCACTGTCCCCTTGCACTCCCTGTCTTTGAATCTTACTTTCCATCCTTTTCACATAAAACTC<br>TTTTAGGTTACCAAGCTGGACCCCACGAAGTGTTGGATATAAGGAAAACTCTGGAGAGCAGTGAGCCCAGCAGAGCCCCCCTAGGGATCTTCAGCCCCAGCCTGAAGTATTCATTATAGTCA<br>AGAAAGGCTGTGGTTTCACAGGTGCAGGTCCAACCCTCCTTTCTCTGCCTTGTGGCTGCCTGTCCTGGGGCAGGTTACGTTGCCCCTGGGAGCCTCTGTTTTCTGTAA |
| **Primers for gBlock amplification** | |
| HPRTCF_fwd | TTACAGAAAACAGAGGCTCCCAGGG |
| HRPTCF_rev | CTCGGCCCAGGCTGGCCATTCCGGA |
| **Spec-seq/SEAM-seq** | |
| Protospacer | AGGAAACTTCTGGCAGGACC |
| On-target site | ACTGTTTAAGGAAACTTCTGGCAGGACCAGGTCATG |
| **Spec-seq Library** | |
| Lib_1 | GCTATCTAGTGCTCAGTACGC(N:25252525)(N)(N)(N)TTTAAGGAAACTTCTGGCAGGACCAGGTCATGagatcggaagagcacacgtctgaactccagtca |
| Lib_2 | GCTATCTAGTGCTCAGTACGCACTG(N:25252525)(N)(N)(N)AGGAAACTTCTGGCAGGACCAGGTCATGagatcggaagagcacacgtctgaactccagtca |
| Lib_3 | GCTATCTAGTGCTCAGTACGCACTGTTTA(N:25252525)(N)(N)(N)AACTTCTGGCAGGACCAGGTCATGagatcggaagagcacacgtctgaactccagtca |
| Lib_4 | GCTATCTAGTGCTCAGTACGCACTGTTTAAGGA(N:25252525)(N)(N)(N)TCTGGCAGGACCAGGTCATGagatcggaagagcacacgtctgaactccagtca |
| Lib_5 | GCTATCTAGTGCTCAGTACGCACTGTTTAAGGAAACT(N:25252525)(N)(N)(N)GCAGGACCAGGTCATGagatcggaagagcacacgtctgaactccagtca |
| Lib_6 | GCTATCTAGTGCTCAGTACGCACTGTTTAAGGAAACTTCTG(N:25252525)(N)(N)(N)GACCAGGTCATGagatcggaagagcacacgtctgaactccagtca |
| Lib_7 | GCTATCTAGTGCTCAGTACGCACTGTTTAAGGAAACTTCTGGCAG(N:25252525)(N)(N)(N)AGGTCATGagatcggaagagcacacgtctgaactccagtca |
| Lib_8 | GCTATCTAGTGCTCAGTACGCACTGTTTAAGGAAACTTCTGGCAGGACC(N:25252525)(N)(N)(N)CATGagatcggaagagcacacgtctgaactccagtca |
| Lib_9 | GCTATCTAGTGCTCAGTACGCACTGTTTAAGGAAACTTCTGGCAGGACCAGGT(N:25252525)(N)(N)(N)agatcggaagagcacacgtctgaactccagtca |
| **Reverse primer for Klenow extension of Spec-seq library** | |
| Spec-Rev | TGACTGGAGTTCAGACGTGT |
| **Primers for NGS Library amplification (P5)** | |
| Spec-i5-1 | AATGATACGGCGACCACCGAGATCTACACTCTTTCCCTACACGACGCTCTTCCGATCTTAACTAGAGGCTATCTAGTGCTCAGTACGC |
| Spec-i5-2 | AATGATACGGCGACCACCGAGATCTACACTCTTTCCCTACACGACGCTCTTCCGATCTATCATGCTGAGCTATCTAGTGCTCAGTACGC |
| Spec-i5-3 | AATGATACGGCGACCACCGAGATCTACACTCTTTCCCTACACGACGCTCTTCCGATCTGATGCACATCTGCTATCTAGTGCTCAGTACGC |
| Spec-i5-4 | AATGATACGGCGACCACCGAGATCTACACTCTTTCCCTACACGACGCTCTTCCGATCTCGATGGTTCGACGCTATCTAGTGCTCAGTACGC |
| Spec-i5-5 | AATGATACGGCGACCACCGAGATCTACACTCTTTCCCTACACGACGCTCTTCCGATCTTCGATCGCACTTCGCTATCTAGTGCTCAGTACGC |
| Spec-i5-6 | AATGATACGGCGACCACCGAGATCTACACTCTTTCCCTACACGACGCTCTTCCGATCTATCGATAGTTGCTTGCTATCTAGTGCTCAGTACGC |
| Spec-i5-7 | AATGATACGGCGACCACCGAGATCTACACTCTTTCCCTACACGACGCTCTTCCGATCTGATCGATCCAGTTAGGCTATCTAGTGCTCAGTACGC |
| Spec-i5-8 | AATGATACGGCGACCACCGAGATCTACACTCTTTCCCTACACGACGCTCTTCCGATCTCGATCGATTTGAGCCTGCTATCTAGTGCTCAGTACGC |
| Spec-i5-9 | AATGATACGGCGACCACCGAGATCTACACTCTTTCCCTACACGACGCTCTTCCGATCTACGATCGATACACGATCGCTATCTAGTGCTCAGTACGC |
| Spec-i5-10 | AATGATACGGCGACCACCGAGATCTACACTCTTTCCCTACACGACGCTCTTCCGATCTTACGATCGATAGTCCAGAGCTATCTAGTGCTCAGTACGC |
| **Primers for NGS Library amplification (P7)** | |
| i7_IDT-1 | CAAGCAGAAGACGGCATACGAGATACGATCAGGTGACTGGAGTTCAGACGTGT |
| i7_IDT-2 | CAAGCAGAAGACGGCATACGAGATTCGAGAGTGTGACTGGAGTTCAGACGTGT |
| i7_IDT-3 | CAAGCAGAAGACGGCATACGAGATCTAGCTCAGTGACTGGAGTTCAGACGTGT |
| i7_IDT-4 | CAAGCAGAAGACGGCATACGAGATATCGTCTCGTGACTGGAGTTCAGACGTGT |
| i7_IDT-5 | CAAGCAGAAGACGGCATACGAGATTCGACAAGGTGACTGGAGTTCAGACGTGT |
| i7_IDT-6 | CAAGCAGAAGACGGCATACGAGATCCTTGGAAGTGACTGGAGTTCAGACGTGT |
| i7_IDT-7 | CAAGCAGAAGACGGCATACGAGATATCATGCGGTGACTGGAGTTCAGACGTGT |
| i7_IDT-8 | CAAGCAGAAGACGGCATACGAGATTGTTCCGTGTGACTGGAGTTCAGACGTGT |
| i7_IDT-9 | CAAGCAGAAGACGGCATACGAGATATTAGCCGGTGACTGGAGTTCAGACGTGT |
| i7_IDT-10 | CAAGCAGAAGACGGCATACGAGATCGATCGATGTGACTGGAGTTCAGACGTGT |
| i7_IDT-11 | CAAGCAGAAGACGGCATACGAGATGATCTTGCGTGACTGGAGTTCAGACGTGT |

| | |
|---|---|
| i7_IDT-12 | CAAGCAGAAGACGGCATACGAGATAGGATAGCGTGACTGGAGTTCAGACGTGT |

**Supplementary Table 2 DNA sequences used in the *in vitro* characterization of sequence specificity / activity of AsCas12a mutants**

| Gene | Assay | Cas12a crRNA | Cas12a PAM | Cas9 sgRNA | Forward primer | Reverse primer |
|---|---|---|---|---|---|---|
| **A1CF** | A1CF-5-S | UGGGAUGCCCCCAACAAAUAA | TTTT | UAAUCGGCAGUUGUCCACAC | acactctttccctacacgacgctcttccgatctCACCTTCAGTAACCTTTTTCATCT | gtgactggagttcagacgtgtgctcttccgatctTCAACACTATGAACCCAAACATC |
| | A1CF-7-S | GGAUGCCCCCAACAAAUAAUC | TTTG | | | |
| | A1CF-48-AS | UGCCAGUGUGGACAACUGCCG | TTTG | | | |
| | A1CF-125-AS | AACCUGAAUCCUACAAGGAUU | TTTC | | | |
| **AADACL3** | AADACL3-44-S | CGCUUUGGGACAAUCCCUGUG | TTTC | GGCAUCGUGUACUACCACGG | acactctttccctacacgacgctcttccgatctGCTCAGAATCTGTTCTATGCC | gtgactggagttcagacgtgtgctcttccgatctGGTCTGAGAAAATGGTTCTTACT |
| **ABCG2** | ABCG2-50-S | AUGAUCCCACUGUAAACACAA | TTTC | ACCUGGUCUCAACGCCAUCC | acactctttccctacacgacgctcttccgatctCACATTAAAAGTGCACAGAAAACG | gtgactggagttcagacgtgtgctcttccgatctCAGTGCCTACCCTAAATTAATAGAA |
| | ABCG2-69-AS | GUGUUUACAGUGGGAUCAUGA | TTTT | | | |
| **ABCG5** | ABCG5-127-AS | UGUCUCCUGCAGGUGGAGGCC | TTTG | CAUGUGGCAGACCGACUGAU | acactctttccctacacgacgctcttccgatctGCTAGTAACAGTTCTGGGTG | gtgactggagttcagacgtgtgctcttccgatctCCCATGAATTGAAATAGCAGC |
| **ABHD6** | ABHD6-34-S | GGUUUGAAUCCCACAGGCCUG | TTTG | GAUUGUCAGUUGAGUACUGC | acactctttccctacacgacgctcttccgatctTATTAACTCTGGGCTGCTGT | gtgactggagttcagacgtgtgctcttccgatctGACATAGGAGCAGAGCTGAAG |
| | ABHD6-80-S | UACAACGGCUCAAAGAACUGC | TTTG | | | |
| **ABRAXAS1** | ABRAXAS1-55-AS | CUGCUAUUAACACCAAGUAUA | TTTT | AAGCUGCUCUACUCAUCGAC | acactctttccctacacgacgctcttccgatctCAGTGACTGGCCAACATTTA | gtgactggagttcagacgtgtgctcttccgatctCTGCTTCACAAAAACTTGCAG |
| **ACAT1** | ACAT1-102-AS | CAAUCAAAUCUUCAAGCUUUA | TTTA | UCAAGCUUUACCCCACCAUA | acactctttccctacacgacgctcttccgatctGGGTTTGCAAAATATTTGTATTAACATT | gtgactggagttcagacgtgtgctcttccgatctCAGTATTGGACATTAGATAGCATTTAT |
| **ACVR2A** | ACVR2A-24-AS | AUUGGUUCUGUCUUUUUCCCA | TTTG | ACUGGUGUUGAACCGUGUUA | acactctttccctacacgacgctcttccgatctTAGGTGCTATACTTGGTAGATCAGAAA | gtgactggagttcagacgtgtgctcttccgatctCAGAAATATTCTTCCAGGTAGCAAAA |
| **ADAM30** | ADAM30-45-AS | ACUCGUAUGAAGUCACCAUUC | TTTG | UACGAGUCAAACUCCCCUUC | acactctttccctacacgacgctcttccgatctCTTTTAACTGCAGTAGGTAGGA | gtgactggagttcagacgtgtgctcttccgatctGACAATGCTCCTTAAGTCTCTT |
| **ADAM7** | ADAM7-44-S | UUUUUCCUUUAUGAGUUUUCC | TTTC | GCCAGUAUCAGUACGUGUAA | acactctttccctacacgacgctcttccgatctTCATTGTTTTTACCAAGGATCCAT | gtgactggagttcagacgtgtgctcttccgatctTAAATCATAGGCTACAGCTGAAA |
| | ADAM7-63-S | CCUAUGGAUUUUCAAAGAAGU | TTTT | | | |
| | ADAM7-105-S | AUCACUCCUGGUAUAUAGAAA | TTTT | | | |
| **ADGRL3** | ADGRL3-44-AS | CACCACGCCCCAGAUUGGUGG | TTTG | UUUGAGUCCGACCACCAAUC | acactctttccctacacgacgctcttccgatctCCTTCCAACAGTTTTTCTTTGTC | gtgactggagttcagacgtgtgctcttccgatctGATGAATACTCAGTCAGGGTATCA |
| **AGAP9** | AGAP9-13-S | CUUCUUUAGGUGUUUCUUUUU | TTTT | UGGUGGUGCUGGAGAUACCG | acactctttccctacacgacgctcttccgatctTTCAAAGTGCCACGTTTG | gtgactggagttcagacgtgtgctcttccgatctCTAGCCACATCAGCCT |
| | AGAP9-21-S | GGUGUUUCUUUUUAUUGGCAU | TTTA | | | |
| **AHCTF1** | AHCTF1-51-AS | UCGUAUCUGUUAAUAAAAUGG | TTTA | AAGGAUAACAUCGUUACCAC | acactctttccctacacgacgctcttccgatctGCAGAACCCACTAATACAAAGGA | gtgactggagttcagacgtgtgctcttccgatctCACAGACAAGCCCTCAGATATATT |
| | AHCTF1-38-S | UUAACAGAUACGAUAAAGACU | TTTA | | | |
| | AHCTF1-89-AS | AAUAGGUAAGUACAUCUUUUG | TTTT | | | |
| | AHCTF1-90-AS | UAAUAGGUAAGUACAUCUUUU | TTTT | | | |

| | | | | | | |
|---|---|---|---|---|---|---|
| **AHCYL1** | AHCYL1-9-S | UAAGAAGUAUCCAAACGUGUU | TTTA | CGGAUCUUCUUAAACACGUU | acactctttccctacacgacgctcttccgatctGTCTTCTGGCTGTTCTATAGATC | gtgactggagttcagacgtgtgctcttccgatctTCCAGAATCTGTTACCTGTGAA |
| **AIDA** | AIDA-28-AS | UUACCAAGGUUGCCAUCGGAA | TTTA | CCAAGGUUGCCAUCGGAACC | acactctttccctacacgacgctcttccgatctCCAGCATCTTTCAAACCAATTTT | gtgactggagttcagacgtgtgctcttccgatctACTGTGAAATGATATGGAGCTTTT |
| **AKAP10** | AKAP10-70-S | CAUGGAAACUUGGUGAGUUCC | TTTC | AGAAGCGGGACUAUUUCUAC | acactctttccctacacgacgctcttccgatctCACATATGTACACACAAGAAAATCACATA | gtgactggagttcagacgtgtgctcttccgatctAATAATAGAACTAACAGCACTCAGAATCA |
| **AKIRIN1** | AKIRIN1-40-AS | CUCUAAUAAAAAAGAAACAAA | TTTG | AAGAAUAUAGUCGUUAUCAG | acactctttccctacacgacgctcttccgatctAAGATCTTTGTCTCTTCCCACTT | gtgactggagttcagacgtgtgctcttccgatctCACAAGCTTCACTCTGATTAAGAA |
| | AKIRIN1-31-S | UUAUUAGAGCAAAUUUUUCAG | TTTT | | | |
| | AKIRIN1-56-AS | UGUUCUGAAAAAUUUGCUCUA | TTTA | | | |
| | AKIRIN1-50-S | AGAACAUAAAACAAGAAUAUA | TTTC | | | |
| **AKR1B10** | AKR1B10-70-AS | GGGAAAAGGUCAUCCCCAGAC | TTTG | GAACGUUGCUUUUCCACCGA | acactctttccctacacgacgctcttccgatctAGATGTGGTATTTAGCAAGAGTCA | gtgactggagttcagacgtgtgctcttccgatctCATGGATAACTGAAGATTTCTCTCC |
| | AKR1B10-68-S | CCCAAAGAUGAUAAAGGUAAU | TTTC | | acactctttccctacacgacgctcttccgatctAGATGTGGTATTTAGCAAGAGTCA | gtgactggagttcagacgtgtgctcttccgatctCATGGATAACTGAAGATTTCTCTCC |
| | AKR1B10-99-AS | CACCGAUGGCAUUACCUUUAU | TTTC | | acactctttccctacacgacgctcttccgatctAGATGTGGTATTTAGCAAGAGTCA | gtgactggagttcagacgtgtgctcttccgatctCATGGATAACTGAAGATTTCTCTCC |
| **ALS2** | ALS2-22-S | GUUCCGGCACAUACUGCUGGU | TTTG | ACAAUGGUGUCGCGUACAUG | acactctttccctacacgacgctcttccgatctCGCCAACTGTAAAATCCTGA | gtgactggagttcagacgtgtgctcttccgatctTAGCCCCATTCTAGAAAATGC |
| **AMZ2** | AMZ2-36-AS | AAAAGAAAUACAUAAAUGUAU | TTTA | AUCUUUACCCAAGAGACUCG | acactctttccctacacgacgctcttccgatctCATGCAGGTGAATTACACGA | gtgactggagttcagacgtgtgctcttccgatctAATACCATCTGTCAAAGAGGC |
| | AMZ2-24-S | UGUAUUUCUUUUUAAAUAGGG | TTTA | | | |
| **ANKRD13D** | ANKRD13D-57-AS | CCAGAGACACGGCCAGCUCCA | TTTC | CUUCGACACAAUGCCAACGU | acactctttccctacacgacgctcttccgatctCTCTTCTCTCCTGCCCTTT | gtgactggagttcagacgtgtgctcttccgatctTTGTCCTCTGCAGTACCTG |
| **ANKRD27** | ANKRD27-56-AS | CAGGUCUUAGUACCCUGCAAA | TTTC | UAGACUGGAUGCUGCUCGAC | acactctttccctacacgacgctcttccgatctGGTCTGAAAATGCTCTTCCA | gtgactggagttcagacgtgtgctcttccgatctTTCTGCTGAGGTGGTAAATGG |
| | ANKRD27-58-AS | UCCAGGUCUUAGUACCCUGCA | TTTT | | | gtgactggagttcagacgtgtgctcttccgatctTTCTGCTGAGGTGGTAAATGG |
| **ANKRD45** | ANKRD45-39-S | AAAGUUUCCAAACGACCCCAG | TTTC | CAAAGUUUCCAAACGACCCC | acactctttccctacacgacgctcttccgatctCTGAGAATATCTAGCAGCAACAT | gtgactggagttcagacgtgtgctcttccgatctGTCTGTTTGATCTCACCATCTT |
| **ANKRD50** | ANKRD50-43-S | GAAAACCUAAAGAAGAGAUAA | TTTC | UCGGAAGGCAUAUAUCGUCA | acactctttccctacacgacgctcttccgatctCGCAAAGCTTCTTCTTGATCTAAAC | gtgactggagttcagacgtgtgctcttccgatctGTTGACATTCTCTTTGAAGATATGGT |
| | ANKRD50-61-AS | UCUCUUCUUUAGGUUUUCGAA | TTTA | | | |
| | ANKRD50-62-AS | AUCUCUUCUUUAGGUUUUCGA | TTTT | | | |
| **ANTXRL** | ANTXRL-63-AS | AGCCUAAGGGAAAGAAGGCAU | TTTG | GGGGGCCAACGUUUACACCC | acactctttccctacacgacgctcttccgatctCACATGCATTTCAGGACACT | gtgactggagttcagacgtgtgctcttccgatctGGTTGGCTCGAAAGTGAC |
| **AP1S3** | AP1S3-9-S | UCCAGCAGCUCCACGUAACGA | TTTG | AGAGAUUGUGCAUCGUUACG | acactctttccctacacgacgctcttccgatctGGGACTGTAGCTAATCCTAAC | gtgactggagttcagacgtgtgctcttccgatctTTCGTGGGAAAAACTGTCTC |
| | AP1S3-70-S | CUAUUGCACAGCAAAAAUAUA | TTTT | | | |
| | AP1S3-89-AS | UAUUUUUGCUGUGCAAUAGAA | TTTA | | | |
| | AP1S3-105-AS | AAGGUAUGCUAGUUUAUAUUU | TTTC | | | |

| | | | | | | |
|---|---|---|---|---|---|---|
| | AP1S3-110-AS | AUUUCAAGGUAUGCUAGUUUA | TTTC | | | |
| **AP3B1** | AP3B1-57-AS | GGUGAAUAUUUUUGCACUAUU | TTTT | AAGCGCUUGCACGAAUUAGU | acactctttccctacacgacgctcttccgatctAGCATCATGATAGGTACAATAATTGG | gtgactggagttcagacgtgtgctcttccgatctTGCTATTGTCATATTACACCCTTTAAG |
| | AP3B1-80-AS | AAAUAUUGUAAAGUUGAAAUU | TTTA | | | |
| **APAF1** | APAF1-46-AS | AUCAGAAGCCCAGAUUUGUCU | TTTC | UCUUUGCACACGGUUGGAUC | acactctttccctacacgacgctcttccgatctGGCCTTAAGTTCATTATTCTTTCC | gtgactggagttcagacgtgtgctcttccgatctTAGCCTCTTCAATATTAAGTGGAA |
| **APOPT1** | APOPT1-54-AS | AGGUAUGUAAAAGUGAACAGG | TTTC | UGUAAAAGUGAACAGGUCGA | acactctttccctacacgacgctcttccgatctAAAGGTCTCAAGATTCTGCC | gtgactggagttcagacgtgtgctcttccgatctGTTTGCCCAGAACTGTTGATT |
| **ARHGAP36** | ARHGAP36-17-S | UUUUUUCUCUUUCUGUAGCUC | TTTC | AAGGUGUUCGGACGGUCAAU | acactctttccctacacgacgctcttccgatctCAGATCAGGGCATTGGGAT | gtgactggagttcagacgtgtgctcttccgatctCCTCTGCTGTCCTCTCAA |
| **ARL1** | ARL1-22-S | GAAAUGCCAAUUCGGUCUCGG | TTTG | CAUGGCAACUAACUCUGAUU | acactctttccctacacgacgctcttccgatctAATGACTGCCCCACATTTTA | gtgactggagttcagacgtgtgctcttccgatctCTCTAGGCCATACTGGAGAT |
| **ARL16** | ARL16-98-AS | UCCUAUAGGUGGGCACCAAUC | TTTT | UGCCACGAUGUCAGUAAGAU | acactctttccctacacgacgctcttccgatctGCCCATATAGGATTACAACCC | gtgactggagttcagacgtgtgctcttccgatctCTCTTCAGGAGTTCACAACG |
| **ARL6** | ARL6-26-S | AAUCACAUUAUGGGAUUGCUA | TTTG | UGCCACUAUUAUCUAGCCCA | acactctttccctacacgacgctcttccgatctCCAATATTTTCCATAACTTAAGGTGC | gtgactggagttcagacgtgtgctcttccgatctGCATCTAACAAAGATACTTACATTTGAA |
| **ARMCX1** | ARMCX1-47-S | CUUAUAAAAUUGAUGAUAUUC | TTTC | UUGAACUUGCCUCUCCGGAC | acactctttccctacacgacgctcttccgatctGGGCAAGTGGAAAATCCAAG | gtgactggagttcagacgtgtgctcttccgatctTTGTTACCCAGAGTGACCAA |
| | ARMCX1-86-AS | GGAGGUCGGGAGCACUCAGAA | TTTT | | | gtgactggagttcagacgtgtgctcttccgatctTTGTTACCCAGAGTGACCAA |
| **ARR3** | ARR3-18-S | CACCACCGGAGGCAGGCCCUG | TTTG | AGUACAAUUUGCACCACCGG | acactctttccctacacgacgctcttccgatctCTGGCTAATCTCTTGGTCTCT | gtgactggagttcagacgtgtgctcttccgatctATCAGTGAAGAAAGGGCATCA |
| **ATP1B4** | ATP1B4-79-AS | UCCUCCUCUUCUUCCUCCUCC | TTTC | GACGGUGGUGCCCAAAUCGG | acactctttccctacacgacgctcttccgatctCACTGTTCCAGGATGATCC | gtgactggagttcagacgtgtgctcttccgatctCTCTGGATCCCACAGGTATT |
| | ATP1B4-102-AS | CCUCCUCUUCCUCCUCUUCUU | TTTT | | | |
| **AUTS2** | AUTS2-53-AS | CGGAGUCCAUGGCCCCGCGUC | TTTG | AUGGACUCCGCAAAAAGCGG | acactctttccctacacgacgctcttccgatctTTTTGTGTGGCTGCGG | gtgactggagttcagacgtgtgctcttccgatctCTTGTCGGAGCCCGAC |
| **BANF1** | BANF1-16-S | UUUGGGAUUCCUAGAUUAAGC | TTTT | CCAAAAGCACCGAGACUUCG | acactctttccctacacgacgctcttccgatctTCTTACTATGAGATTGCTCGTGG | gtgactggagttcagacgtgtgctcttccgatctACACCTTGTCAAAACCCCTTTC |
| | BANF1-58-AS | GGAGGUUGUCAUCUUGAUCAG | TTTG | | | gtgactggagttcagacgtgtgctcttccgatctACACCTTGTCAAAACCCCTTTC |
| **BBOX1** | BBOX1-27-AS | UUGAGGGUGGAGAGCCACUUG | TTTC | ACCGGAGCAUCUGACAAACC | acactctttccctacacgacgctcttccgatctCAGCTACCCACTTTGGATTTT | gtgactggagttcagacgtgtgctcttccgatctGTCCTCAGTTGGGAACTATTT |
| | BBOX1-80-AS | AAACUUCUCCUGGUUUGUCAG | TTTG | | | |
| | BBOX1-90-AS | CCAAGUUUUGAAACUUCUCCU | TTTC | | | |
| | BBOX1-91-AS | CCCAAGUUUUGAAACUUCUCC | TTTT | | | |
| | BBOX1-122-S | AUGGGUGAGUCACCAAAUGGU | TTTT | | | |
| **BLOC1S1** | BLOC1S1-44-AS | UGGGGAGAGGGGGAAAAGGAA | TTTC | GAUCCACCAAAGCUUCUGUC | acactctttccctacacgacgctcttccgatctCAACTAGCAGAATAGTAATGGATGG | gtgactggagttcagacgtgtgctcttccgatctGAGGAAACTGATGTTGATAAGAGGT |
| | BLOC1S1-32-S | CCCCUCUCCCCAGAAAAGAGG | TTTC | | | gtgactggagttcagacgtgtgctcttccgatctGAGGAAACTGATGTTGATAAGAGGT |
| **BLVRA** | BLVRA-11-S | GCGUGGUGGUGGUUGGUGUUG | TTTG | ACACGAAGCCAAUCAGGUUC | acactctttccctacacgacgctcttccgatctCCTACAGTGTTTTCAGACTCCA | gtgactggagttcagacgtgtgctcttccgatctCTGGGCTTTCCATGAATTATGAA |

| | | | | | | |
|---|---|---|---|---|---|---|
| **BMT2** | BMT2-59-S | CGCUCCUGCUCCCGGGGCGGC | TTTC | UGGUGAAGAGCGUCCACCGG | acactctttccctacacgacgctcttccgatctTTCCTCCCTCACTCAGC | gtgactggagttcagacgtgtgctcttccgatctGCCGAAATACTGCTCGT |
| **BOLL** | BOLL-44-AS | AAAAAGGAUUUCCAUCUAUUA | TTTA | GGUGGGACCGAAGUUACCUC | acactctttccctacacgacgctcttccgatctGGTTGATGTGTGTTATTATTTGTAATTAT | gtgactggagttcagacgtgtgctcttccgatctCTAACAACTTCAACTGGATATCCTTATA |
| | BOLL-44-S | UAAAUGAAAUUACUUACAGGC | TTTT | | | |
| | BOLL-46-S | AAUGAAAUUACUUACAGGCCA | TTTA | | | |
| **BRAF** | BRAF-28-S | GGAUUACUUACUCAAGUUGGU | TTTT | GAACUUCUGUACUACAACGC | acactctttccctacacgacgctcttccgatctCCTCACAGACTTTTAGACATCGTAG | gtgactggagttcagacgtgtgctcttccgatctTCACCTTAGCATTTTGTGACTTTT |
| | BRAF-29-S | GAUUACUUACUCAAGUUGGUC | TTTG | | | |
| **BRD8** | BRD8-66-S | CUGCUUGACGAGCUAGAACAU | TTTA | CCGGAGGUUACCCACUGUGA | acactctttccctacacgacgctcttccgatctCTCTTCCATAGTGGTTGGAGT | gtgactggagttcagacgtgtgctcttccgatctCTGCTTTAGTAATGCAACATACCT |
| | BRD8-87-AS | AUGUUCUAGCUCGUCAAGCAG | TTTT | | | |
| **BRDT** | BRDT-89-AS | AAAACUACAACAAACUUACUU | TTTA | UUCUCCCUUGAACGUGGUAC | acactctttccctacacgacgctcttccgatctCGCAACAGAAAAAGTATTTAAGCAG | gtgactggagttcagacgtgtgctcttccgatctTTGCTAGACGCTGAAGACTAATTTT |
| **BRIX1** | BRIX1-57-AS | CUCUUGGUUGCCGCCAUCUUG | TTTC | GCAACCAAGAGGAAACGGCG | acactctttccctacacgacgctcttccgatctGAGCCGCCGAACCATA | gtgactggagttcagacgtgtgctcttccgatctCGGCTCCGCATCTATTTC |
| **BTN3A1** | BTN3A1-32-AS | CAGGCCGAGGAGGGAACUUCU | TTTC | GAGGGAACUUCUGAUGGUAC | acactctttccctacacgacgctcttccgatctAGTAGCAGCATCTGTGATCAT | gtgactggagttcagacgtgtgctcttccgatctAGGTTGACTGCAGACACTAA |
| **BUD23** | BUD23-54-AS | CUUUGGCACUGUUAGGGUAGU | TTTG | GUAGUCUACCACCAUGCCAC | acactctttccctacacgacgctcttccgatctGCTGCCTTTCTTTCCTCA | gtgactggagttcagacgtgtgctcttccgatctGCTATGCCACTACCCTCC |
| **C10orf111** | C10orf111-8-S | AAAUGUUAUUCGGCCGCUUCA | TTTA | UCGGAGUCGCUGCAAAGUCG | acactctttccctacacgacgctcttccgatctGTCTTCTAAGTTCTGGCCAA | gtgactggagttcagacgtgtgctcttccgatctGAATATCCCGTCCAGTGTC |
| **C10orf67** | C10orf67-62-AS | CUCCUCCUUGAGGGGGACCUU | TTTC | GGGGGACCUUUGGCACACGC | acactctttccctacacgacgctcttccgatctTGAATTCCCGAGCTTCTCG | gtgactggagttcagacgtgtgctcttccgatctGATCGCAGGGCTCATTATGT |
| **C16orf82** | C16orf82-28-S | CUCGAGGGAGAAAAAGGGGAA | TTTT | AUCCUCUGUCCAGAAUGAGC | acactctttccctacacgacgctcttccgatctTCACAGCCAACATTCAGAG | gtgactggagttcagacgtgtgctcttccgatctGCTCTAAGCTGGGTGACT |
| **C17orf102** | C17orf102-37-AS | GCAGCCGCUCUUCCAGAGGCG | TTTT | AAGGAUGCACGCUCCCCACC | acactctttccctacacgacgctcttccgatctTGATTCCCAGCCAGTG | gtgactggagttcagacgtgtgctcttccgatctCGTTTCTCCAGGGTAGA |
| **C1D** | C1D-31-AS | UUGUUGUAUUCCUGAGGUUUG | TTTG | AACCCAAGGAGUUAAUCCUA | acactctttccctacacgacgctcttccgatctGCTCACTTTCATAATTTCAACTCGAATT | gtgactggagttcagacgtgtgctcttccgatctCATTCACCATTTTATTCCATGAAATTTTT |
| **C1orf61** | C1orf61-69-S | ACUCACCGCCCGGGAGCUGUC | TTTC | UCUAUCGUCCACACGGAGGA | acactctttccctacacgacgctcttccgatctCAAAGCTGACTGCAAACAATT | gtgactggagttcagacgtgtgctcttccgatctTCTCTTCCCTTTAGCTTCTCAC |
| **C1orf68** | C1orf68-27-AS | GAUGUAGGUCAUCUGGCAGGG | TTTT | CACCCUGGACAUAGCACGUC | acactctttccctacacgacgctcttccgatctCAACCTATGTAAAATGCCCAA | gtgactggagttcagacgtgtgctcttccgatctCTTGCAGGAGCTTGAACATA |
| **C2orf16** | C2orf16-26-AS | GGGUCAACCCCACAGAUUUCU | TTTG | GGGUUGACCCCAAAGCUAAC | acactctttccctacacgacgctcttccgatctCAGAATCTTCAGAAATGGCACAA | gtgactggagttcagacgtgtgctcttccgatctATTTTGGTACCTGAAGATCTGG |
| **C2orf74** | C2orf74-22-AS | CUGCCUUGGAAACUGAAAACA | TTTC | CCUUGUACAGAUGCAAACGG | acactctttccctacacgacgctcttccgatctCACCTTAAAATCAGGGCCATT | gtgactggagttcagacgtgtgctcttccgatctATTGCTTGTCACCACTTTGG |
| | C2orf74-8-S | AGUUUCCAAGGCAGGAAAGGU | TTTC | | | |

**Supplementary Table 3 Genomic targets used to compare the activity of WT and AsCas12a Ultra in HEK293 and Jurkat cells.**

| Assay | AsCas12a crRNA | LbCas12a crRNA |
|---|---|---|
| HPRT 38104-S | uaauuucuacucuuguagaucuugggugugu uaaaagugacca | uaauuucuacuaaguguagaucuugggugugu uaaaagugacca |
| HPRT 38115-AS | uaauuucuacucuuguagauacacacccaaggaaagacuau | uaauuucuacuaaguguagauacacacccaaggaaagacuauga |
| HPRT 38228-S | uaauuucuacucuuguagauuaauuaacagcuugcuggugaaa | uaauuucuacuaaguguagauuaauuaacagcuugcuggugaaa |
| HPRT 38330-AS | uaauuucuacucuuguagaugguuaaagaugguuaaaugau | uaauuucuacuaaguguagaugguuaaagaugguuaaaugauug |

| T7EI primer | |
|---|---|
| HPRT-FWD | AAGAATGTTGTGATAAAAGGTGATGCT |
| HPRT-RWD | ACACATCCATGGGACTTCTGCCTC |

**Supplementary Table 4 Genomic targets used to compare the activity of LbCas12a and AsCas12a Ultra in HEK293 cells at lower temperature.**

| Name | PAM | crRNA Sequence |
|---|---|---|
| Cpf1 HPRT 38094-S | TTTV | auagucuuuccuugggugugu |
| Cpf1 HPRT 38104-S | | cuugggugugguuaaaagugac |
| Cpf1 HPRT 38115-AS | | acacacccaaggaaagacuau |
| Cpf1 HPRT 38146-AS | | auccgugcugaguguaccaug |
| Cpf1 HPRT 38164-AS | | uaaacacuguuucauuucauc |
| Cpf1 HPRT 38164-S | | gaaacgucagucuucucuuuu |
| Cpf1 HPRT 38186-S | | uaaugcccuguagucucucug |
| Cpf1 HPRT 38228-S | | uaauuaacagcuugcuggugа |
| Cpf1 HPRT 38330-AS | | gguuaaagaugguuaaaugau |
| Cpf1 HPRT 38343-S | | ugugaaauggcuuauaauugc |
| Cpf1 HPRT 38455-S | | guuguuggauuugaaauucca |
| HPRT_Cpf1_TATV_1 | TATV | gggauuacuaggaaggggcag |
| HPRT_Cpf1_TATV_2 | | aaauggagagcuaaauuaugg |
| HPRT_Cpf1_TATV_3 | | uuauaugucacauuuuguaau |
| HPRT_Cpf1_TATV_4 | | ugucacauuuuguaauuaaca |
| HPRT_Cpf1_TATV_5 | | ucacauuuuguaauuaacagc |
| HPRT_Cpf1_TATV_6 | | agccagacuguaagugaauua |
| HPRT_Cpf1_TATV_7 | | caacacuucguggggguccuuu |
| HPRT_Cpf1_TATV_8 | | ugaaauggcuuauaauugcuu |
| HPRT_Cpf1_TATV_9 | | auugcuuagagaauauuugua |
| HPRT_Cpf1_TATV_10 | | uaaaugaugaauuaugauucu |
| HPRT_Cpf1_TATV_11 | | auucuuuuuaguuguuggauu |
| HPRT_Cpf1_TYCV_1 | TYCV | uaguaaucccccauaauuuagc |
| HPRT_Cpf1_TYCV_2 | | cauaauuuagcucuccauuuc |
| HPRT_Cpf1_TYCV_3 | | uuucauagucuuuccuugggu |
| HPRT_Cpf1_TYCV_4 | | uagucuuuccuugggugguguu |
| HPRT_Cpf1_TYCV_5 | | uugggugugguuaaaagugacc |
| HPRT_Cpf1_TYCV_6 | | uuucauccgugcugaguguac |
| HPRT_Cpf1_TYCV_7 | | uccgugcugaguguaccaugg |
| HPRT_Cpf1_TYCV_8 | | ugcugaguguaccauggucac |
| HPRT_Cpf1_TYCV_9 | | ccagcaagcuguuaauuacaa |
| HPRT_Cpf1_TYCV_10 | | acacuucguggggguccuuuuc |
| HPRT_Cpf1_TYCV_11 | | cuuacagucuggcuuauaucc |

**Supplementary Table 5 Genomic targets used to compare the editing efficiency of AsCas12a RR and RVR variants ± Ultra mutations.**

| Gene | Assay | crRNA | PAM | Donor sequence |
|---|---|---|---|---|
| **ALDH2** | ALDH21203-S | CUUACAAUACUGGUAGUCAGU | TTTC | T*G*CTTTTTCTTGTTTTTTTTTTTCCTTACAATACTGGTAGGAATTCTCAGTATTAGGTTGACAGCTGGTCCTGAGAACTTCTTTCCTTC*T*C |
| | ALDH2169-S | CUGAGUGAGUGAAGUAAUAAU | TTTG | T*C*GCAGGTGTTCATTAAGTGTTTGCTGAGTGAGTGAAGTAGAATTCATAATCTAGGGCTGGGCATGGTGACTCACACCTGCAATCCCAG*G*A |
| | ALDH2299-AS | UUGUAGAGAUGAGGUCCCUCU | TTTC | G*T*TCTTTTATTTACCTATTGTTTTTTGTAGAGATGAGGTCGAATTCCCTCTATATTACCCAGGCTGTCTTGAACTCCACTCAAGAGATC*G*A |
| | ALDH2994-S | UUACACAGCAAUGGAUAACUA | TTTG | G*C*TGCTAAGTTGTGGGCTGTTTTGTTACACAGCAATGGATGAATTCAACTAACACATGAGGCCTGGCGAGCACAGAGCAAAGCTGCCCA*G*G |
| **APOC** | APOC1198-AS | UCAAAGUAUCAGCAAACUUAU | TTTA | T*G*TTGTGCAGCACCTAACAGTTTATCAAAGTATCAGCAAAGAATTCCTTATTGTCCTGTTTGACCTTCCGCACAAAGCTGTCAAGGAAG*G*C |
| | APOC639-S | GGGGCUGGGUGACCGAUGGCU | TTTA | C*T*GTTGCTTCCCCTGACTGATTTAGGGGCTGGGTGACCGAGAATTCTGGCTTCAGTTCCCTGAAAGACTACTGGAGCACCGTTAAGGAC*A*A |
| | APOC668-AS | AGGGAACUGAAGCCAUCGGUC | TTTC | T*T*AACGGTGCTCCAGTAGTCTTTCAGGGAACTGAAGCCATGAATTCCGGTCACCCAGCCCCTAAATCAGTCAGGGGAAGCAACAGAGCA*G*G |
| | APOC921-AS | UUGGGAGGCCAGCAUGCCUGG | TTTA | A*T*AGCAGCTTCTTGTCCAGCTTTATTGGGAGGCCAGCATGGAATTCCCTGGAGGGGGGCCAGGCATGAGGTGGGGTAGGAGAGCACTGA*G*A |
| **AR** | AR1407-AS | AGGUCAGCGGAGCAGCUGCUU | TTTA | C*T*GGCCTCGCTCAGGATGTCTTTAAGGTCAGCGGAGCAGCGAATTCTGCTTAAGCCGGGGAAAGTGGGGCCCAGCAGGGACAACGTGGA*T*G |
| | AR454-AS | GCUACUGAAGACCUGACUGCC | TTTG | T*G*TTTTTGTTTGTTTTGTTTTTTGGCTACTGAAGACCTGAGAATTCCTGCCTTTTCATCTTTTGATCTCTGGCTCCGCACTCGGGGTGG*G*G |
| | AR490-AS | UUUCGGCUUUUUUGUUUUUGU | TTTA | C*T*GAGTTATTATCTTTTTCTTTTATTTCGGCTTTTTTGTTGAATTCTTTGTTTGTTTTGTTTTTTGGCTACTGAAGACCTGACTGCCTT*T*T |
| | AR710-S | CGUGGUUGCUCCCGCAAGUUU | TTTG | T*T*GAGGCTGTCAGAGCGCTTTTTGCGTGGTTGCTCCCGCAGAATTCAGTTTCCTTCTCTGGAGCTTCCCGCAGGTGGGCAGCTAGCTGC*A*G |
| **BCAT** | BCAT1172-AS | ACAGUGAAAAGAUCGUUCUCC | TTTA | G*T*TTTATATGCGATTGCAGTTTTCCAGTGTGTGAAACTAGGAATTCGAAAACAAAACACTCAACGGTGTACATCCCTACACCTAAATAG*T*C |
| | BCAT1396-AS | CAGUGUGUGAAACUAGGAAAA | TTTC | T*A*AAACCCGAAGAGCAACTTTTTCCAAGGAAAGTGAAGGAGAATTCTGGGCAGATATTCTTGCCATCTAACATTTTGGAATTGAGATGA*C*C |
| | BCAT1440-S | CAAGGAAAGUGAAGGAUGGGC | TTTC | T*C*CCTCCCCTGCTTGTGAAGTTTGGAAAGGAAGTGAGGGCGAATTCTTCTCTCAGTTTCTCCTATGCACAGGAGGTGGGGAATTTTGGA*G*A |
| | BCAT989-S | GAAAGGAAGUGAGGGCUUCUC | TTTG | A*A*TAAGAGGGGCCTTCTGCTTTTAACAGTGAAAAGATCGTGAATTCTCTCCCTCCCCTCTCCACCCGGGTCAACTCTTCCAGCCGCTCC*C*T |
| **BCL2** | BCL21031-AS | AUGAGGCACGUUAUUAUUAGU | TTTC | C*A*ATTCCTTTCGGATCTTTATTTCATGAGGCACGTTATTAGAATTCTTAGTAAGTATTGTTAATATCAGTCTACTTCCTCTGTGATGCT*G*A |
| | BCL21058-AS | UUCCAAUUCCUUUCGGAUCUU | TTTA | G*G*CATGAGATGCAGGAAATTTTTATTCCAATTCCTTTCGGGAATTCATCTTTATTTCATGAGGCACGTTATTATTAGTAAGTATTGTTA*A*T |
| | BCL2636-AS | AAACUUCCCAAUGAAUCAGGA | TTTG | C*A*CTCTCCAGTTATAGCTGATTTGAAACTTCCCAATGAATGAATTCCAGGAGTCGCGGGGAGAGGGAGTAAAAATTAGGAGGATTTCCA*G*A |
| | BCL2922-AS | CCUGUACACACUGAGUGAAAG | TTTC | T*A*AGTAAAAAATCAGGTGCGTTTCCCTGTACACACTGAGTGAATTCGAAAGCAGGGCATACACACTACAAGTAACACGGCTAAAAAGAA*T*G |
| **C5** | C5245-AS | GCACUAUGAAGAGUGGCUCAA | TTTG | A*A*GGGACCCTAAAATGGGAATTTGGCACTATGAAGAGTGGGAATTCCTCAAAGCTAGAAACCAGTAAGCCCATTGCTTTGGCCTTTCTT*C*T |
| | C5450-S | AAAGGAGACUAUCCUCAAAAG | TTTA | A*A*TTCTTCAAGTTCAGTTTATTTAAAAGGAGACTATCCTCGAATTCAAAAGTGTTATATCCGTGGTTTCCTGCTACCTCCAACCATGGG*C*C |
| | C5562-AS | CUCCUGUCCCCAGGUUUUCCC | TTTG | G*A*TAGCTTGTTTTACTTACGTTTGCTCCTGTCCCCAGGTTGAATTCTTCCCCAGGAAGATTAAAAAACAAAGTATTCCCAAAAGGCCCA*T*G |
| | C5729-S | AUUUGAUGAGUAGCUAACAAC | TTTG | T*G*AGAAAGTCATGGAAGCATTTTGATTTGATGAGTAGCTAGAATTCACAACATGACAGCTTTTATGGAATTGTTATTTATTAAAATTTA*C*G |
| **CTNNB1** | CTNNB1113-S | CCCUCCCUGGCUUUUAUUAUU | TTTC | A*G*CTGATTTTTGAATAGTGTTTTCCCCTCCCTGGCTTTTAGAATTCTTATTACAACTCTGTGCTTTTTCATCACCATCCTGAATATCTA*T*A |
| | CTNNB11210-AS | GUUCUCAAAACUGCAUUCUGA | TTTA | A*A*ACTATTATACACTAACTTTTTAGTTCTCAAAACTGCATGAATTCTCTGACTTTCAGTAAGGCAATGAAAAATAATACTCTTACCAGC*T*A |
| | CTNNB11293-S | GGAUAGCAAAUACUUAGGUAA | TTTA | T*A*GCAATGTCACTTTTACCATTTAGGATAGCAAATACTTAGAATTCGGTAAATGCTGAACTGTGGATAGTGAGTGTTGAATTAACCTTT*T*C |
| | CTNNB1803-S | UUGGCUGUCUUUCAGAUUUGA | TTTC | A*C*CCTGGCTATCATTCTGCTTTTCTTGGCTGTCTTTCAGAGAATTCTTTGACTTTATTTCTAAAAATATTTCAATGGGTCATATCACAG*A*T |
| **CYP27A1** | CYP27A1131-AS | GAAUUAUAGAAUCACCAAAAU | TTTA | G*A*AATCCCATACTCCTAGAATTTAGAATTATAGAATCACCGAATTCAAAATCTCAAGTGACAGAATCTTAGAATCCCCAAACCAGAATC*T*C |

| | | | | |
|---|---|---|---|---|
| | CYP27A1600-S | CUACAUCCUGUUCGAGAAACG | TTTG | C*T*CTGTGCACTACTCAGCTATTTGCTACATCCTGTTCGAGGAATTCAAACGCATTGGCTGCCTGCAGCGATCCATCCCCGAGGACACCG*T*G |
| | CYP27A1791-S | GUGAGGACUCCCAGAUGGGGC | TTTG | G*T*TGGAATGCCATCTTTTCCTTTGGTGAGGACTCCCAGATGAATTCGGGGCCCAGGGAAGAGAGATGGGGGTGACTCCAGGTCTGTGCA*T*C |
| | CYP27A1961-S | ACAGGGAAGAAGCUGAUUGAU | TTTC | C*C*TTTCCTCTTCTCTGTTGCTTTCACAGGGAAGAAGCTGAGAATTCTTGATGAGAAGCTCGAAGATATGGAGGCCCAACTGCAGGCAGC*A*G |
| **CYP7A1** | CYP7A1139-S | UAAUAGAAUGAACAAAUGGCU | TTTC | C*A*GTTACTACCACTTTTTTTTTCTAATAGAATGAACAAAGAATTCTGGCTAATTGTTTGCTTTGTCAACCAAGCTCAAGTTAATGGAT*C*T |
| | CYP7A1441-S | CCUUCAUUUUUAUACAGUAAA | TTTA | T*G*ATTCATCCATTTAATTTTTTTACCTTCATTTTTATACAGAATTCGTAAATTTGGTTTTCTATACTTACACATATTAGCATTATCTTC*C*T |
| | CYP7A1512-AS | AUUUAAAAAAUAAGGAAGAUA | TTTC | T*T*AAAAATTCAAATCAAATTTTTCATTTAAAAAATAAGGAGAATTCAGATAATGCTAATATGTGTAAGTATAGAAAACCAAATTTACTG*T*A |
| | CYP7A1591-S | GUAUUGGCAUAUACCGAUGGU | TTTA | G*A*CATATGACAGCTTCCCTTTTTAGTATTGGCATATACCGGAATTCATGGTAATATATAAATGTATATTGGTGTTAAACATAACTGACA*G*A |
| **DICER** | DICER33497-AS | CUUAGGAAAGUGUCUGUAAAC | TTTC | T*C*TTCACATAGTGCATGTATTTTCCTTAGGAAAGTGTCTGGAATTCTAAACAATAAAAATTTCCTGTGCAGCTCCTCTTGCTCATGTTT*G*A |
| | DICER33625-S | AAAGCGUUGAGUGGUAUAAUA | TTTG | A*A*CCATATGAGCGACAGCAGTTTGAAAGCGTTGAGTGGTAGAATTCTAATAATAGAAATCAGGATAATTATGTGTCATGGAGTGATTCT*G*A |
| | DICER33765-S | UGCGGAAUUAUUUUUGUGGAA | TTTG | C*C*TTCTCCTTTTACCAACATTTTGTGCGGAATTATTTTTGGAATTCTGGAAAGAAGATACACAGCAGTTGTCTTAAACAGGTAAATCTG*T*A |
| | DICER33781-S | UGGAAAGAAGAUACACAGCAG | TTTG | A*C*ATTTTGTGCGGAATTATTTTTGTGGAAAGAAGATACACGAATTCAGCAGTTGTCTTAAACAGGTAAATCTGTAAGTTGACTCCTTTC*A*C |
| **EGFR** | EGFR1320-AS | UCAUGGGCAGCUCCUUCAGUC | TTTC | C*C*AGCCTCTCACCCTGTAAATTTCTCATGGGCAGCTCCTTGAATTCCAGTCCGGTTTTATTTGCATCATAGTTAGATAAGACTGCTAAG*G*C |
| | EGFR439-S | CCAGCUAAUGUCAAGUAAUAA | TTTA | G*A*GTTTTATGAGAAAGCCATTTTACCAGCTAATGTCAAGTGAATTCAATAACTAGAAAAGGATATCAAATAGAAACAGGCTAATCTGGA*G*T |
| | EGFR461-AS | UAGUUAUUACUUGACAUUAGC | TTTC | C*T*GTTTCTATTTGATATCCTTTTCTAGTTATTACTTGACAGAATTCTTAGCTGGTAAAATGGCTTTCTCATAAAACTCTCGTTTGTAGC*T*C |
| | EGFR759-S | GAUGGGGUUUACUUAGCCUUC | TTTG | G*C*TTCAGAGTTTCACTGAATTTTGGATGGGGTTTACTTAGGAATTCCCTTCTTCTGTTTTTCTTTTACCTTTCCTTTTTAAATAAGAAA*T*A |
| **EMX1** | EMX110685-S | CAGCUAUCAGCUAUUUGUCAA | TTTC | C*C*AGGCAAGCTTACCTGTCATTTCCAGCTATCAGCTATTTGAATTCGTCAAGCATCAGTCACCCCCAACAGCCCCCCTGCCATGCATTT*C*T |
| | EMX110839-AS | AUGAGAGCAGACAGAAGUAUA | TTTA | A*G*CCAGTTTTGGGGAGGCAGTTTAATGAGAGCAGACAGAAGAATTCGTATATGGCTAGATGTAAAACAGCTACGGGGTGGGACAGATTA*G*A |
| | EMX110875-S | CGAUUUUCUAUUAGUUAUAUG | TTTC | C*C*AAAACTGGCTAATTTATATTTCCGATTTTCTATTAGTTGAATTCATATGTTCTTCCTAACAATAAGAACTATTTACCCATTTGTTCA*T*T |
| | EMX110955-S | CCUUCUGUGUUUCCACCAUUC | TTTA | T*C*ATTATCTTTGTCATCTATTTTACCTTCTGTGTTTCCACGAATTCCATTCATCTCAAACTTCATCTCCATAGAGCTATTTCGCAACTG*A*C |
| | EMX117707-AS | UGGUUGCCCACCCUAGUCAUU | TTTG | C*A*GCACTCTGCCCTCGTGGGTTTGTGGTTGCCCACCCTAGGAATTCTCATTGGAGGTGACATCGATGTCCTCCCCATTGGCCTGCTTCG*T*G |
| | EMX117983-S | UUUUGAUGCAUUUCUGUUUUA | TTTG | T*C*CGTGTCTCCAATCTCCCTTTTGTTTTGATGCATTTCTGGAATTCTTTTAATTTATTTTCCAGGCACCACTGTAGTTTAGTGATCCCC*A*G |
| | EMX118014-S | CAGGCACCACUGUAGUUUAGU | TTTC | G*C*ATTTCTGTTTTAATTTATTTTCCAGGCACCACTGTAGTGAATTCTTAGTGATCCCCAGTGTCCCCCTTCCCTATGGGAATAATAAAA*G*T |
| | EMX118033-S | GUGAUCCCCAGUGUCCCCCUU | TTTA | T*T*TTCCAGGCACCACTGTAGTTTAGTGATCCCCAGTGTCCGAATTCCCCTTCCCTATGGGAATAATAAAAGTCTCTCTCTTAATGACAC*G*G |
| **ERBB2** | ERBB2468-S | AAUUAAAGUUCCCGGAUUUUU | TTTA | C*T*CCGCTGAAGTCCACACAGTTTAAATTAAAGTTCCCGGAGAATTCTTTTTGTGGGCGCCTGCCCCGCCCCTCGTCCCCCTGCTGTGTC*C*A |
| | ERBB2579-AS | CUCAUUAACCCAUCAGGCGUC | TTTG | G*A*TCATGGAAAACACTTCAGTTTGCTCATTAACCCATCAGGAATTCGCGTCCGCCTTCCCTTAACCCTATCGCCTCGATATATGGACAC*A*G |
| | ERBB2612-S | AGGUAGGGCUGUUUACUGUCA | TTTG | A*A*GTGTTTTCCATGATCTTTTTTGAGGTAGGGCTGTTTACGAATTCTGTCACCACCCCTGTCGGATTTTACTTCCTAAACGTACCTGTA*A*C |
| | ERBB2627-S | CUGUCACCACCCCUGUCGGAU | TTTA | T*C*TTTTTTGAGGTAGGGCTGTTTACTGTCACCACCCCTGTGAATTCCGGATTTTACTTCCTAAACGTACCTGTAACTATCCACTTCTCT*C*C |
| **G6PC** | G6PC177-S | GGGGACCAGGAGGGCAGACCC | TTTC | G*T*CCTGTGTCTCTGGCCTGGTTTCGGGGACCAGGAGGGCAGAATTCGACCCTTGCACTGCCAAGAAGCATGCCAAAGTTAATCATTGGC*C*C |
| | G6PC753-S | AGUGGUAAGAACCAUAUAGAG | TTTA | G*A*GACTGGCTCAACCTCGTCTTTAAGTGGTAAGAACCATAGAATTCTAGAGAGGAGATCAGCAAGAAAAGAGGCTGGCATTCGCTCTCG*C*A |
| | G6PC923-AS | UGUCUUCCCCCUUUAUGUAGA | TTTC | T*C*AACTCACTGGTAGGATTTTTTCTGTCTTCCCCCTTTATGAATTCGTAGATGATCCAAAGTCAGAGAGAGGGGTTGGAAGCATGAGTA*G*C |

| | | | | |
|---|---|---|---|---|
| | G6PC945-AS | AACUCACUGGUAGGAUUUUUU | TTTC | T*G*AAATAGGCTTTCCTGTATTTTCAACTCACTGGTAGGATGAATTCTTTTTCTGTCTTCCCCCTTTATGTAGATGATCCAAAGTCAGAG*A*G |
| **GCK** | GCK1282-S | CUGUGAAACACUGGGGGGAAU | TTTG | G*A*GAGCTGGAGACAGGGGACTTTGCTGTGAAACACTGGGGGAATTCGGAATGTGCCTGCATCACCCCAGCTGGGGGCCCAGGCAGAGTG*G*G |
| | GCK1284-AS | ACAGCAAAGUCCCCUGUCUCC | TTTC | C*A*GGCACATTCCCCCCAGTGTTTCACAGCAAAGTCCCCTGGAATTCTCTCCAGCTCTCTCCTTGCTCACTGCTGGGCTCAGGGCTGGCT*C*A |
| | GCK526-AS | AUUGUUCUCCAACGAGUCGGC | TTTC | T*T*CAGTCACAGTTTCCTCCTTTTCATTGTTCTCCAACGAGGAATTCTCGGCAAGCATTCCCTTCTGGACCCTTCCTCACTGAGCCTGGA*T*G |
| | GCK560-AS | GGGUUUGAGGUUCAGUCACAG | TTTG | T*G*TGGTTCTCCTCGGGCTGGTTTGGGGTTTGAGGTTCAGTGAATTCCACAGTTTCCTCCTTTTCATTGTTCTCCAACGAGTCGGCAAGC*A*T |
| **GYS2** | GYS21201-S | GGAAGAUGUAUAUGUGAUUAG | TTTA | A*G*TATAATTAAACATTTTATTTTAGGAAGATGTATATGTGGAATTCATTAGAACCAGTTGGAAACCTCAACTCTACATTCTGTTAGTAA*T*T |
| | GYS21337-AS | CCUUUUGAGAAGCUCCUGAAU | TTTG | T*T*CTGAAAGGAGCACAGTATTTTGCCTTTTGAGAAGCTCCGAATTCTGAATTAAAACATTAATTCCAAGCTAATTGTGAATATGAAACA*T*T |
| | GYS2950-AS | CUCACUCACUAGCAAUUGGUU | TTTC | T*A*TATATTCCGTTTAAATGCTTTCCTCACTCACTAGCAATGAATTCTGGTTATCACTAGAGAGTTATCTGTGAAATCTTACTCTACATT*C*C |
| | GYS2959-AS | AAUGCUUUCCUCACUCACUAG | TTTA | T*A*GCCTAAATATATATTCCGTTTAAATGCTTTCCTCACTCGAATTCACTAGCAATTGGTTATCACTAGAGAGTTATCTGTGAAATCTTA*C*T |
| **HPRT** | HPRT 38115-AS | ACACACCCAAGGAAAGACUAU | TTTA | C*T*GAGTGTACCATGGTCACTTTTAACACACCCAAGGAAAGGAATTCACTATGAAATGGAGAGCTAAATTATGGGGATTACTAGGAAGGG*G*C |
| | HPRT 38228-S | UAAUUAACAGCUUGCUGGUGA | TTTG | C*T*GTATGTTATATGTCACATTTTGTAATTAACAGCTTGCTGAATTCGGTGAAAAGGACCCCACGAAGTGTTGGATATAAGCCAGACTGT*A*A |
| | HPRT 38330-AS | GGUUAAAGAUGGUUAAAUGAU | TTTA | G*C*CATTTCACATAAAACTCTTTTAGGTTAAAGATGGTTAAGAATTCATGATTGACAAAAAAAGTAATTCACTTACAGTCTGGCTTATAT*C*C |
| | HPRT38082-S | GCUCUCCAUUUCAUAGUCUUU | TTTA | C*T*TCCTAGTAATCCCCATAATTTAGCTCTCCATTTCATAGGAATTCTCTTTCCTTGGGTGTGTTAAAAGTGACCATGGTACACTCAGCA*C*G |
| | HPRT38146-AS | AUCCGUGCUGAGUGUACCAUG | TTTC | C*G*TTTCTAAACACTGTTTCATTTCATCCGTGCTGAGTGTAGAATTCCCATGGTCACTTTTAACACACCCAAGGAAAGACTATGAAATGG*A*G |
| | HPRT38330-AS | GGUUAAAGAUGGUUAAAUGAU | TTTA | G*C*CATTTCACATAAAACTCTTTTAGGTTAAAGATGGTTAAGAATTCATGATTGACAAAAAAAGTAATTCACTTACAGTCTGGCTTATAT*C*C |
| | HPRT38628-S | AACAGACUGAUGGUUCCCAUU | TTTG | C*T*CCTTCCAGCACCTCATAATTTGAACAGACTGATGGTTCGAATTCCCATTAGTCACATAAAGCTGTAGTCTAGTACAGACGTCCTTAG*A*A |
| | HPRT411-AS | CCAGGGCUCCCCCGAGGAGGG | TTTC | C*T*TCCCACACGCAGTCCTCTTTTCCCAGGGCTCCCCCGAGGAATTCGAGGGACCCACCCCAAACCCCGCCATTCCGTCCTCCCTGCCGC*C*C |
| | HPRT677-S | CACACACUGUAGUUCAUCUUU | TTTG | G*A*CCAGGTTTTGCCTTTAGTTTTGCACACACTGTAGTTCAGAATTCTCTTTATGGAGATGCTCATGGCCTCATTGAAGCCCCACTACAG*C*T |
| | HPRT782-AS | CCUAGUUCCUUCGUGUGUCAA | TTTC | C*T*TGAAATGACCTAATGCCTTTTCCCTAGTTCCTTCGTGTGAATTCGTCAAATACGCATGGTTACCGCTACCAGAGCTGTAGTGGGGCT*T*C |
| | HPRT803-S | AAGCCGAAAUUCACAUGUGCU | TTTC | A*G*GGAAAAGGCATTAGGTCATTTCAAGCCGAAATTCACATGAATTCGTGCTAGAATCCAGATTCCATGCTGACCGATGCCCCAGGATAT*A*G |
| **MET** | MET1379-S | UUUACUUCUUGACGGUCCAAA | TTTA | A*T*GCCTTTGAAAGCAACAATTTTATTTACTTCTTGACGGTGAATTCCCAAAGGGAAACTCTAGATGCTCAGACTTTTCACACAAGAATA*A*T |
| | MET540-S | UCUUCAUUUCUGACAACUGAA | TTTC | A*A*TGGTATAGGTCTTTCAGTTTTCTCTTCATTTCTGACAAGAATTCCTGAACTGCTCTCGCCTTGAACCTGTTTTGGCAGATAAACCTC*T*C |
| | MET793-S | CUUGGUGCCACUAACUACAUU | TTTC | A*T*TCTACATGAGCATCACATTTTCCTTGGTGCCACTAACTGAATTCACATTTATGTTTTAAATGAGGAAGACCTTCAGAAGGTTGCTGA*G*T |
| | MET892-S | CCAUGUCAGGACUGCAGCAGC | TTTC | G*T*GCTGGAACACCCAGATTGTTTCCCATGTCAGGACTGCAGAATTCGCAGCAAAGCCAATTTATCAGGAGGTGTTTGGAAAGATAACAT*C*A |
| **MYC** | MYC238-AS | GUGGAUGCGGCAAGGGUUGCG | TTTC | G*C*CCGCTGCTATGGGCAAAGTTTCGTGGATGCGGCAAGGGGAATTCTTGCGGACCGCTGGCTGGGGGATCAGCGGGAGGGCTGGGCCAG*A*G |
| | MYC528-S | UCCCAUUCCUGCGCUAUUGAC | TTTC | A*A*TGCCTAAATAGGGTGTCTTTTCTCCCATTCCTGCGCTAGAATTCTTGACACTTTTCTCAGAGTAGTTATGGTAACTGGGGCTGGGGT*G*G |
| | MYC801-AS | UGCCCACCGCAAAGCAACCCC | TTTC | G*A*GCTCAGGATGCAAGGGGCTTTCTGCCCACCGCAAAGCAGAATTCACCCCCAGCCCCCCAAAACCCAGAGAGCAATTAACACAATAAA*G*C |
| | MYC932-S | UGACAGCCGGAGACGGACACU | TTTC | T*T*AAATTTCGGCTCACCGCATTTCTGACAGCCGGAGACGGGAATTCACACTGCGGCGCGTCCCGCCCGCCTGTCCCCGCGGCGATTCCA*A*C |
| **RAG2** | RAG2207-S | CUCAUUCAGUCCCACUGCAAG | TTTC | A*C*TGACCTAACTCCTTGGATTTTCCTCATTCAGTCCCACTGAATTCGCAAGCGTGTGGGAGGACTTAAAAAAATGCTATTCACATGTGA*A*G |
| | RAG2504-AS | CAUCCAGAUGGAAAACUCCAG | TTTA | T*C*AGTTTGACATGGTTATGCTTTACATCCAGATGGAAAACGAATTCTCCAGTGGGGCAGGATCTTTTGGGCCAGCCTTTTTGTCCAAAG*A*A |

| | | | | |
|---|---|---|---|---|
| | RAG2544-S | UCUAAGGAUUCCUGCUACCUC | TTTC | G*T*CAAACTGAAGCCTACAATTTTCTCTAAGGATTCCTGCTGAATTCACCTCCCTCCTCTTCGCTACCCAGCCACTTGCACATTCAAAGG*C*A |
| | RAG2907-S | CUGGUGGAUUUUGAAUUUGGG | TTTC | G*C*TGACTGCCTGCCCTGTGTTTTCCTGGTGGATTTTGAATGAATTCTTGGGTGTGCTACATCATACATTCTTCCAGAACTTCAGGATGG*G*C |
| **STAT3** | STAT346810-AS | GUUUCCUGGGUCAUACAGUCC | TTTG | A*T*TTCTCAGGAAAAGAAACATTTGAACTGAATTTGAAGGTGAATTCGAGTGAGTTCAGGTGTGTTTGGGCTGAAGCCCAGGCCATGCTG*A*G |
| | STAT347118-S | AACUGAAUUUGAAGGUGAGUG | TTTG | A*G*GGACAGAGGGTAAATATTTTTGGTTTCCTGGGTCATACGAATTCAGTCCCTGCCACAAATAGCCCTGCCACTGCAGCACCAAAGCAG*C*C |
| | STAT347155-S | GGCUGAAGCCCAGGCCAUGCU | TTTG | G*G*TGAGTGAGTTCAGGTGTGTTTGGGCTGAAGCCCAGGCCGAATTCATGCTGAGTGGATAGCGGGTGGGAAGAGAGTGTGGAAACACAC*T*G |
| | STAT347205-AS | CACACUCUCUUCCCACCCGCU | TTTC | T*C*TTCCCTGCATGCAGTGTGTTTCCACACTCTCTTCCCACGAATTCCCGCTATCCACTCAGCATGGCCTGGGCTTCAGCCCAAACACAC*C*T |
| **TTR** | TTR1166-S | CUUAGCUAGGAAGUGACCAGG | TTTG | G*A*AGGATGCCCTCTTTTTGTTTTGCTTAGCTAGGAAGTGAGAATTCCCAGGAACCTGAGCATCATTTAGGGGCAGACAGTAGAGAAAAG*A*A |
| | TTR1297-AS | AUCACCUACAUAAAGUGUUCU | TTTC | C*T*GGGCATAGAATGTTTACTTTTCATCACCTACATAAAGTGAATTCGTTCTGTGACCCAAAAGGGTTGCAAACCACAGCTAGAGGAGAG*G*A |
| | TTR1439-S | GGAAAAGGUAUAAUGUGUAUU | TTTA | A*A*GTGTCCTTGTCTTAGAATTTTAGGAAAAGGTATAATGTGAATTCGTATTAACCCATTAACAAAAGGAAAGGAATTCAGAAATATTAT*T*A |
| | TTR959-AS | UGAACACAUGCACGGCCACAU | TTTC | C*C*CAGGTGTCATCAGCAGCCTTTCTGAACACATGCACGGCGAATTCCACATTGATGGCAGGACTGCCTCGGACAGCATCTAGAACTTTG*A*C |

**Supplementary Table 6 Genomic targets used to compare the HDR efficiency mediated by WT or AsCas12a Ultra in Jurkat cells.**

| Gene | Assay | Crispr enzyme | sgRNA | donor_sequence |
|---|---|---|---|---|
| **A1CF** | Hs.Cas9.A1CF.1 | Cas9 | UAAUCGGCAGUUGUCCACAC | TTTTGCAGAAATGGGCGCCTCTTAGGGGTTTGTGCCAGTGGAATTCTGGACAACTGCCGATTATTTGTTGGGGGCATCCCAAAAAC |
| | Cas12a_A1CF-48-AS | Cas12a | UGCCAGUGUGGACAACUGCCG | AAATGGGCGCCTCTTAGGGGTTTGTGCCAGTGTGGACAACGAATTCTGCCGATTATTTGTTGGGGGCATCCCAAAAACCAAAAAGAGAGAA |
| **AADACL3** | Hs.Cas9.AADACL3.1 | Cas9 | GGCAUCGUGUACUACCACGG | ATGGTTCTTACTCAAACTCCCCATGACGCCCCCGCCACCGGAATTCTGGTAGTACACGATGCCAGGCTTCAGGGTGCAGGTGGATG |
| | Cas12a_AADACL3-44-S | Cas12a | CGCUUUGGGACAAUCCCUGUG | CCCGATGTTGTGGTCACGGATTTCCGCTTTGGGACAATCCGAATTCCTGTGAAGCTGTACCAACCCAAGGCATCCACCTGCACCCTGAAGC |
| **ABCG5** | Hs.Cas9.ABCG5.1 | Cas9 | CAUGUGGCAGACCGACUGAU | CTCACCCGTGGAAATGCCCCCCAAGCTGTAGTTGCCAATCGAATTCAGTCGGTCTGCCACATGGCTCAGACTCAGCTCTGCCATGA |
| | Cas12a_ABCG5-127-AS | Cas12a | UGUCUCCUGCAGGUGGAGGCC | GACTCCCAGCCTTCCTGTAATTTGTGTCTCCTGCAGGTGGGAATTCAGGCCGTCATGGCAGAGCTGAGTCTGAGCCATGTGGCAGACCGAC |
| **ABHD6** | Hs.Cas9.ABHD6.1 | Cas9 | GAUUGUCAGUUGAGUACUGC | TATCATCAAGCCATTTGGGTTTGAATCCCACAGGCCTGCAGAATTCGTACTCAACTGACAATCAATTTGTACAACGGCTCAAAGAA |
| | Cas12a_ABHD6-34-S | Cas12a | GGUUUGAAUCCCACAGGCCUG | GTGGATGTATCATCAAGCCATTTGGGTTTGAATCCCACAGGAATTCGCCTGCAGTACTCAACTGACAATCAATTTGTACAACGGCTCAAAG |
| **ABRAXAS1** | Hs.Cas9.ABRAXAS1.1 | Cas9 | AAGCUGCUCUACUCAUCGAC | TTAACACCAAGTATAATAACAGAAAGCTGCTCTACTCATCGAATTCGACTGGAACATTCCTTATATAAACCTCAAAAAGGGTAAAT |
| | Cas12a_ABRAXAS1-55-AS | Cas12a | CUGCUAUUAACACCAAGUAUA | TTTTCAAACCAAGACCTTGTTTTTCTGCTATTAACACCAAGAATTCGTATAATAACAGAAAGCTGCTCTACTCATCGACTGGAACATTCCT |
| **ACAT1** | Hs.Cas9.ACAT1.1 | Cas9 | UCAAGCUUUACCCCACCAUA | CAATGTTCCATATGTAATGAACAGAGGATCAACACCATATGAATTCGGTGGGGTAAAGCTTGAAGATTTGATTGTAAAAGACGGGC |
| | Cas12a_ACAT1-102-AS | Cas12a | CAAUCAAAUCUUCAAGCUUUA | AGACATCAGTTAGCCCGTCTTTTACAATCAAATCTTCAAGGAATTCCTTTACCCCACCATATGGTGTTGATCCTCTGTTCATTACATATGG |
| **ADAM30** | Hs.Cas9.ADAM30.1 | Cas9 | UACGAGUCAAACUCCCCUUC | CCTTAAGTCTCTTGGCGAAGATGTAATTTTTCACCCTGAAGAATTCGGGGAGTTTGACTCGTATGAAGTCACCATTCCTGAGAAGC |
| | Cas12a_ADAM30-45-AS | Cas12a | ACUCGUAUGAAGUCACCAUUC | TTTTTCACCCTGAAGGGGAGTTTGACTCGTATGAAGTCACGAATTCCATTCCTGAGAAGCTGAGCTTCCGGGGAGAGGTGCAGGGTGTGGT |
| **ADAM7** | Hs.Cas9.ADAM7.1 | Cas9 | GCCAGUAUCAGUACGUGUAA | AAGGAAAAAGAAAGATATTCTGCATTACCTTAGACCATTAGAATTCCACGTACTGATACTGGCAGCTGAATCATATTCGTGTACTA |
| | Cas12a_ADAM7-63-S | Cas12a | CCUAUGGAUUUUCAAAGAAGU | CTTTCTTTTTCCTTTATGAGTTTTCCTATGGATTTTCAAAGAATTCGAAGTTAACTTTGTTGATTTTGTTTTATCACTCCTGGTATATAGA |
| **ADGRL3** | Hs.Cas9.ADGRL3.1 | Cas9 | UUUGAGUCCGACCACCAAUC | GCTGCAGATAAAATTTATTTCATGCCCTGGACTCCCTATCGAATTCGTACCGATACTTTAATAGAATATGCTTCTTTAGAAGATTT |
| | Cas12a_ADGRL3-44-AS | Cas12a | CACCACGCCCCAGAUUGGUGG | TCAGATGCCTGCAGAGGGTCTTTGCACCACGCCCCAGATTGAATTCGGTGGTCGGACTCAAACAAATGTTCACTCTGGTATACTCCTTTTA |
| **AGAP9** | Hs.Cas9.AGAP9.1 | Cas9 | UGGUGGUGCUGGAGAUACCG | ACATATAGCTTTGGAGTTTAACCTTTCTGCCAATCCAGAGGAATTCTCAAGCACAATATTCCAGAGGAACTCTCAAACAGAAGGTG |
| | Cas12a_AGAP9-21-S | Cas12a | GGUGUUUCUUUUUAUUGGCAU | AGTTGTTGGTGCTTTTCTTCTTTAGGTGTTTCTTTTTATTGAATTCGGCATGAGGAGAGGGGGGCGGGTTGAGCTTGGGGCTGGTGGTGCT |
| **AHCTF1** | Hs.Cas9.AHCTF1.1 | Cas9 | AAGGAUAACAUCGUUACCAC | CCGTGGGCGCTGCCGTGGCCGCCGACGCGGCTCTCCGCGCGAATTCTTTGCACTCATTGGTACTCAGAGCCTCCCGGGGCCGGCCC |
| | Cas12a_AHCTF1-38-S | Cas12a | UUAACAGAUACGAUAAAGACU | GTCTATCTCATCAATTCCATTTTATTAACAGATACGATAAGAATTCAGACTTTATAGTTTCAAAAGATGTACTTACCTATTAAAAAGCAAA |
| **AKAP10** | Hs.Cas9.AKAP10.1 | Cas9 | AGAAGCGGGACUAUUUCUAC | GGAAATGAGCCTGAAGAGGTGAAGCTGCAGAATGCCAGCAGAATTCAACAGATTGTGCAGAATGCAATCCTGCAAGCTGTGCAGCA |
| | Cas12a_AKAP10-70-S | Cas12a | CAUGGAAACUUGGUGAGUUCC | TGTAGAGGAAGATTCTTGGGTTTCCATGGAAACTTGGTGAGAATTCGTTCCTGCTCTGGCCATTTCAAGACGGAGAGAATGGGCACTGTCA |

| | | | | |
|---|---|---|---|---|
| **AKR1B10** | Hs.Cas9.AKR1B10.1 | Cas9 | GAACGUUGCUUUUCCACCGA | CACTCTGATTAAGAACAACTTCTAAATGTCTCCACCTCTGGAATTCATAACGACTATATTCTTGTTTTATGTTCTGAAAAATTTGC |
| | Cas12a_AKR1B10-68-S | Cas12a | CCCAAAGAUGAUAAAGGUAAU | TCACAGTCTGGGGATGACCTTTTCCCCAAAGATGATAAAGGAATTCGTAATGCCATCGGTGGAAAAGCAACGTTCTTGGATGCCTGGGAGG |
| **ALS2** | Hs.Cas9.ALS2.1 | Cas9 | ACAAUGGUGUCGCGUACAUG | CCGTGACGCGCGCTCCCCTGCGCCCCCGGGGCCTCCCGTGGAATTCGGCTCCGTGCGGGGACAAAGCCAGCGCCAGCAGGAAGAGT |
| | Cas12a_ALS2-22-S | Cas12a | GUUCCGGCACAUACUGCUGGU | AATCAGCAATGCTGACAGGATTTGGTTCCGGCACATACTGGAATTCCTGGTTGGCTACTGCACACTGGCCAGCAGAATTCTCTCCCCACAT |
| **AMZ2** | Hs.Cas9.AMZ2.1 | Cas9 | AUCUUUACCCAAGAGACUCG | ATTCTGACCAGCTGGATCTCGCCCAGTTCCTCGTCCACAGGAATTCTCACGTCGTATGAATCCACCTGGGAAGGAGAACAGGCAAT |
| | Cas12a_AMZ2-24-S | Cas12a | UGUAUUUCUUUUUAAAUAGGG | ATATAATATACACTCATACATTTATGTATTTCTTTTTAAAGAATTCTAGGGGACATCCTGAAGTTCTTGAAAAAGAAGAAACCTGAAGATG |
| **ANKRD13D** | Hs.Cas9.ANKRD13D.1 | Cas9 | CUUCGACACAAUGCCAACGU | GCAGAAGTACCTGACGCGAGTGCCTGGAGTCAGACCACAGGAATTCGCGACAGGTGTAATCGTTCTTAAAATCCATCACTGAGCTA |
| | Cas12a_ANKRD13D-57-AS | Cas12a | CCAGAGACACGGCCAGCUCCA | GCACTCTCACAGACTCCAGGTTTCCCAGAGACACGGCCAGGAATTCCTCCAGTGGGGTCCGCCCGCGGGGTCCTCCTGTTCAATGTCGTG |
| **ANKRD27** | Hs.Cas9.ANKRD27.1 | Cas9 | UAGACUGGAUGCUGCUCGAC | CATCTGTCAAAGAGGCCTGTCCAAAGACAAAATTCCACGAGAATTCGTCTCTTGGGTAAAGATCAATCATTGTTATTCCCACAACA |
| | Cas12a_ANKRD27-56-AS | Cas12a | CAGGUCUUAGUACCCUGCAAA | AATGTTGCCCCGAATTGTTTTTTCCAGGTCTTAGTACCCTGAATTCGCAAAGGAAGCCTGTCGAGCAGCATCCAGTCTACTTGTCAGTTTG |
| **ANKRD45** | Hs.Cas9.ANKRD45.1 | Cas9 | CAAAGUUUCCAAACGACCCC | TGCCCGAAGAAAGAGGCTGCCCGAGATGGCCCAGCCAGTTGAATTCGACCCGGCTCACAATGTCAGCCGCCTGCACCGTGAGTGTC |
| | Cas12a_ANKRD45-39-S | Cas12a | AAAGUUUCCAAACGACCCCAG | ACATCCAGTTCTACCAGTGCTTTCAAAGTTTCCAAACGACGAATTCCCCAGGCTGCAGCACAATGTAAGAGTGTGTACCCTAACAAATGGA |
| **ANTXRL** | Hs.Cas9.ANTXRL.1 | Cas9 | GGGGGCCAACGUUUACACCC | TTGTTAGGGTACACACTCTTACATTGTGCTGCAGCCTGGGGAATTCGTCGTTTGGAAACTTTGAAAGCACTGGTAGAACTGGATGT |
| | Cas12a_ANTXRL-63-AS | Cas12a | AGCCUAAGGGAAAGAAGGCAU | GGCCCCCAGTTTCCGAGCCTTTTGAGCCTAAGGGAAAGAAGAATTCGGCATTGCATATTGGTCATCAAAATACATGGAGCAAAATGCTGGT |
| **AP1S3** | Hs.Cas9.AP1S3.1 | Cas9 | AGAGAUUGUGCAUCGUUACG | TGATCTAAACGATGAAGAATGTACTGCTGAACATCCTTGAGAATTCCGATATATGCCTTCCGAAGGTCATCTAAACTTATTTTTCG |
| | Cas12a_AP1S3-70-S | Cas12a | CUAUUGCACAGCAAAAAUAUA | TCAAGAGCTCATTGTCCTGATTTTCTATTGCACAGCAAAAGAATTCATATAAACTAGCATACCTTGAAATGAAAAATAACAAAAGCAAAAT |
| **AP3B1** | Hs.Cas9.AP3B1.1 | Cas9 | AAGCGCUUGCACGAAUUAGU | ACCGGGCCACCAAGTGTGCTGAGGCTCTGGCACCCCTGTTGAATTCGAGCAGCCTCAACGTGGCTGACAGGAGCGGGCGCAGTGCT |
| | Cas12a_AP3B1-57-AS | Cas12a | GGUGAAUAUUUUUGCACUAUU | AAAATATTGTAAAGTTGAAATTTTGGTGAATATTTTTGCAGAATTCCTATTTTTAGGACCCAAACCAACTAATTCGTGCAAGCGCTTTGAG |
| **APAF1** | Hs.Cas9.APAF1.1 | Cas9 | UCUUUGCACACGGUUGGAUC | GCTGAGATAATGCACATCAAATTGCCTCTGAAACCCAATGGAATTCATCTGAAAAACCGGTCCTCAGCCTTTGGTACACTCAACTG |
| | Cas12a_APAF1-46-AS | Cas12a | AUCAGAAGCCCAGAUUUGUCU | CGTGTGCAAAGATTCTGCAGTTTCATCAGAAGCCCAGATTGAATTCTGTCTTGTTTCCCAACTGAAACCCAATGCACTCCCCCTGGGAAAC |
| **APOPT1** | Hs.Cas9.APOPT1.1 | Cas9 | UGUAAAAGUGAACAGGUCGA | CGGATCCAGAACCTGGCACCGAGCGATGAGGAGTCCAGCTGAATTCGGACAACGTTGTCCCAAGACAGTGCCTCACCCAGCTCCCC |
| | Cas12a_APOPT1-54-AS | Cas12a | AGGUAUGUAAAAGUGAACAGG | TTGTTCCAATGGAGATTCATTTTCAGGTATGTAAAAGTGAGAATTCACAGGTCGAAGGTTTGAATATTTATCTGGGGGTCCTATCCAATCA |
| **ARHGAP36** | Hs.Cas9.ARHGAP36.1 | Cas9 | AAGGUGUUCGGACGGUCAAU | GGCGGCCACGGCGGCTCCAGGCTCTGGGACGCAACCTCTCGAATTCTCGGGGTGGCACTCCAGGGCCGACTGCGGCTGTGAAGGTT |
| | Cas12a_ARHGAP36-17-S | Cas12a | UUUUUUCUCUUUCUGUAGCUC | TCCTATTTGTAAGTGTATCTTTTCTTTTTTCTCTTTCTGTGAATTCAGCTCTGCCTATTGACCGTCCGAACACCTTGGATAAGTGGTTTCT |
| **ARL1** | Hs.Cas9.ARL1.1 | Cas9 | CAUGGCAACUAACUCUGAUU | CGAGCCGGGCATACCTTCAGGCTGCAGTGCACGGCCGACTGAATTCCGGGTGGGTACACGATGAAGTGGCGCAGAGTGAAGCCAAA |

| | | | | |
|---|---|---|---|---|
| | Cas12a_ARL1-22-S | Cas12a | GAAAUGCCAAUUCGGUCUCGG | AACATGGCAACTAACTCTGATTTGGAAATGCCAATTCGGTGAATTCCTCGGTCACAACTGTCTACTACATAAATGACTGCATCTGTGTTTG |
| **ARL16** | Hs.Cas9.ARL16.1 | Cas9 | UGCCACGAUGUCAGUAAGAU | TCAAAATCAGAAACCACTTATCCAAGGTGTTCGGACGGTCGAATTCAATAGGCAGAGCTACAGAAAGAGAAAAAAGAAAAGATACA |
| | Cas12a_ARL16-98-AS | Cas12a | UCCUAUAGGUGGGCACCAAUC | TTGTTCTTTTTGCATCGTGGTTTTTCCTATAGGTGGGCACGAATTCCAATCTTACTGACATCGTGGCACAGAGAAAGATCACCATCCGGGA |
| **ARL6** | Hs.Cas9.ARL6.1 | Cas9 | UGCCACUAUUAUCUAGCCCA | GCAATTCTTATTTTTTAGGAAGAGCTGAGCAATGCCATGTGAATTCCGATGATCCAAGCCATCACTTCCAAACAAGAAGAAATGCA |
| | Cas12a_ARL6-26-S | Cas12a | AAUCACAUUAUGGGAUUGCUA | TTGCAGCTGGTTTGTAAATATTTGAATCACATTATGGGATGAATTCTGCTAGACAGACTTTCAGTCTTGCTTGGCCTGAAGAAGAAGGAGG |
| **ARMCX1** | Hs.Cas9.ARMCX1.1 | Cas9 | UUGAACUUGCCUCUCCGGAC | TCCCGGATGGTGATCTTTCTCTGTGCCACGATGTCAGTAAGAATTCGATTGGTGCCCACCTATAGGAAAAACCACGATGCAAAAAG |
| | Cas12a_ARMCX1-47-S | Cas12a | CUUAUAAAAUUGAUGAUAUUC | TCCGGAGAGGCAAGTTCAACTTTCCTTATAAAATTGATGAGAATTCTATTCTGAGTGCTCCCGACCTCCAAAAGGTCCTCAACATCCTGGA |
| **ARR3** | Hs.Cas9.ARR3.1 | Cas9 | AGUACAAUUUGCACCACCGG | TTGGCCTGAAGAAGAAGGAGGTTCATGTTTTGTGCCTTGGGAATTCGCTAGATAATAGTGGCAAAACGACGATCATTAACAAACTT |
| | Cas12a_ARR3-18-S | Cas12a | CACCACCGGAGGCAGGCCCUG | TGGTTGTCCGGAAAGTACAATTTGCACCACCGGAGGCAGGGAATTCCCCTGGCCCCTCAGCCCAGACCATCCGCCGCTTCCTTCTGTCAGC |
| **ATP1B4** | Hs.Cas9.ATP1B4.1 | Cas9 | GACGGUGGUGCCCAAAUCGG | TTGCTTGGTGACATTTGAAAAGGAACGCAAGGCTCCTGTGGAATTCAGACGAGGTAAAGCCATCTATTATTCAATCAGTGATCCAT |
| | Cas12a_ATP1B4-79-AS | Cas12a | UCCUCCUCUUCUUCCUCCUCC | TCCTCCTCTTCCTCCTCTTCTTTCTCCTCCTCTTCTTCCTGAATTCCCTCCTCCGATTTGGGCACCACCGTCACCCGAGCCTCTTCTTCTG |
| **BANF1** | Hs.Cas9.BANF1.1 | Cas9 | CCAAAAGCACCGAGACUUCG | CGTGACATCACTCCCCATCTCCAGGCATTGGGATCCCGTGGAATTCGCAGTTGCAGCACTGAAGTGGAGAAGGAGACCCAGGAGAA |
| | Cas12a_BANF1-58-AS | Cas12a | GGAGGUUGUCAUCUUGAUCAG | TGCCACGAAGTCTCGGTGCTTTTGGGAGGTTGTCATCTTGGAATTCATCAGGCTTAATCTAGGAATCCCAAAAAAAAGGCAGATTAGGGCT |
| **BBOX1** | Hs.Cas9.BBOX1.1 | Cas9 | ACCGGAGCAUCUGACAAACC | GCTGCGCACCGCGCACAGGCCCCGGTAGAGACCGGCCACCGAATTCTGCAGGACCGCCGGTCCCGCGCCACCCGCAGCCGCCACCA |
| | Cas12a_BBOX1-80-AS | Cas12a | AAACUUCUCCUGGUUUGUCAG | AACCCATCCTTTTCCCAAGTTTTGAAACTTCTCCTGGTTTGAATTCGTCAGATGCTCCGGTGAGTCTTACTATGCCTACTTTCTTGAGGGT |
| **BLOC1S1** | Hs.Cas9.BLOC1S1.1 | Cas9 | GAUCCACCAAAGCUUCUGUC | GAAACCCATCCTTTTCCCAAGTTTTGAAACTTCTCCTGGTGAATTCTTGTCAGATGCTCCGGTGAGTCTTACTATGCCTACTTTCT |
| | Cas12a_BLOC1S1-32-S | Cas12a | CCCCUCUCCCCAGAAAAGAGG | AAGTCTCCCCTCCAATTCCTTTTCCCCCTCTCCCCAGAAAGAATTCAGAGGAGGCGAGAGGCTATCACTGCAGCGACCTGCCTGACAGAAG |
| **BLVRA** | Hs.Cas9.BLVRA.1 | Cas9 | ACACGAAGCCAAUCAGGUUC | GCAAGCCTTCCCAGAAACATGCCGGTGAATCACCAGTTCCGAATTCCCCTGGCCTCATCCATGGACCTTCTGAGCAGCAGGTCCCC |
| | Cas12a_BLVRA-11-S | Cas12a | GCGUGGUGGUGGUUGGUGUUG | TGTTTCAGCCCGAGAGGAAGTTTGGCGTGGTGGTGGTTGGGAATTCTGTTGGCCGAGCCGGCTCCGTGCGGATGAGGGACTTGCGGAATCC |
| **BOLL** | Hs.Cas9.BOLL.1 | Cas9 | GGUGGGACCGAAGUUACCUC | CCCTTTGGATGCTGGTGAATACTGTGTGCCTAGAGGAAATGAATTCCGTAGGCGGTTCCGCGTTAGGCAGCCCATCCTGCAGTATA |
| | Cas12a_BOLL-44-AS | Cas12a | AAAAAGGAUUUCCAUCUAUUA | TTGGCCTGTAAGTAATTTCATTTAAAAAAGGATTTCCATCGAATTCTATTACTATCAATTTTTTAATGTTTGTTATATTTCTGAAATATCT |
| **BRAF** | Hs.Cas9.BRAF.1 | Cas9 | GAACUUCUGUACUACAACGC | GGCTCCACCTGCCCCGGGTTTTGGAGAGGATGTGCCCAATGAATTCAGGCTTGTCGATAACATTGATATGATAGATGGAGATGGAG |
| | Cas12a_BRAF-28-S | Cas12a | GGAUUACUUACUCAAGUUGGU | GGTAGGTAGAAAAGAGATATTTTTGGATTACTTACTCAAGGAATTCTTGGTCATAATTAACACACATCAGTGGAACTTCTGTACTACAACG |
| **BRD8** | Hs.Cas9.BRD8.1 | Cas9 | CCGGAGGUUACCCACUGUGA | CTGGCGGCCGGGGAGCGGCCGTCCGACCCCGGGGAACACCGAATTCCTCAGGCCGAGCCTGGGTCTCTGGCCGAGGGGGCCGGACC |
| | Cas12a_BRD8-66-S | Cas12a | CUGCUUGACGAGCUAGAACAU | GTAACCTCCGGGGGGGTGTTTTTACTGCTTGACGAGCTAGGAATTCAACATAAAAGAGAACAATGTCCAAATCTTCAAGATTAAATAAATG |

| | | | | |
|---|---|---|---|---|
| **BRDT** | Hs.Cas9.BRDT.1 | Cas9 | UUCUCCCUUGAACGUGGUAC | TCCGAGCTGGAGGAGGAGTCACGCCTCCAGGACGCCGACGGAATTCTGTCGGCTGCCTCGTTGCAGTCAGAACTGGCCCACAGCCT |
| | Cas12a_BRDT-89-AS | Cas12a | AAAACUACAACAAACUUACUU | CCAGTGTTAAAAAGCAATGATTTAAAAACTACAACAAACTGAATTCTACTTGGGCCGCAGTTTGTGAACTGGAGTTGACTGAAGCTCCCTG |
| **BRIX1** | Hs.Cas9.BRIX1.1 | Cas9 | GCAACCAAGAGGAAACGGCG | AGGTCCATACCCCACATTGAGGTGATCCACCAAAGCTTCTGAATTCGTCAGGCAGGTCGCTGCAGTGATAGCCTCTCGCCTCCTCT |
| | Cas12a_BRIX1-57-AS | Cas12a | CUCUUGGUUGCCGCCAUCUUG | ACTGCAAAGCCTCCACGCCGTTTCCTCTTGGTTGCCGCCAGAATTCTCTTGCCTCGCCGCCCGCGCTCTTGGCCTCCTTTCCGGCGCTGCC |
| **BUD23** | Hs.Cas9.BUD23.1 | Cas9 | GUAGUCUACCACCAUGCCAC | AGATAAGGGGTAACCCCCACCTGCTGGTCAAGTACCATAGGAATTCTGGGTTCTTCGTGGACGGGAAGTTCCTGTGTTGCCAGCAG |
| | Cas12a_BUD23-54-AS | Cas12a | CUUUGGCACUGUUAGGGUAGU | CGGCCCCCAGCGCTCACTTCTTTGCTTTGGCACTGTTAGGGAATTCGTAGTCTACCACCATGCCACCGGAGAAGCCTGCCTTTGTGGCCTG |
| **C10orf111** | Hs.Cas9.C10orf111.1 | Cas9 | UCGGAGUCGCUGCAAAGUCG | TACTTACCATAATGGTGTTGCTTATTTTCATACTCCAGAGGAATTCGTAACTTCGGTCCCACCGCCTTGGCCTGTAAGTAATTTCA |
| | Cas12a_C10orf111-8-S | Cas12a | AAAUGUUAUUCGGCCGCUUCA | ACAGATCCAGATTCCTGAGCTTTAAAATGTTATTCGGCCGGAATTCCTTCATCACTTGTCCCCGACTTTGCAGCGACTCCGATGGTAGCCT |
| **C10orf67** | Hs.Cas9.C10orf67.1 | Cas9 | GGGGGACCUUUGGCACACGC | GTGGGCAACGTGCCCTTGGAGTGGTACGATGACTTCCCCCGAATTCACGTGGGCTACGACCTGGATGGCAGGCGCATCTACAAGCC |
| | Cas12a_C10orf67-62-AS | Cas12a | CUCCUCCUUGAGGGGGACCUU | TATTAGATGGGTTCACTGCTTTTCCTCCTCCTTGAGGGGGGAATTCACCTTTGGCACACGCTGGGAGGCCATGAAAGCCAAGGCCACCGAG |
| **C16orf82** | Hs.Cas9.C16orf82.1 | Cas9 | AUCCUCUGUCCAGAAUGAGC | CTGCACTCCAGATGGATCCCTCCATCCTCATAACCCTTCAGAATTCCTTCGACGTGAAGATTAGAACCCAGTGCTGTGGAAGGATA |
| | Cas12a_C16orf82-28-S | Cas12a | CUCGAGGGAGAAAAAGGGGAA | CCACTTCAGCTGCCCCCCATTTTTCTCGAGGGAGAAAAAGGAATTCGGGAATCCTCTGTCCAGAATGAGCAGGAAGGAGAGCCAAGCCTAC |
| **C1orf61** | Hs.Cas9.C1orf61.1 | Cas9 | UCUAUCGUCCACACGGAGGA | TCTAAGCTGGGTGACTGTAGGCTTGGCTCTCCTTCCTGCTGAATTCCATTCTGGACAGAGGATTCCCCTTTTTCTCCCTCGAGAAA |
| | Cas12a_C1orf61-69-S | Cas12a | ACUCACCGCCCGGGAGCUGUC | AAAGGGGGTCAGCACGGCCATTTCACTCACCGCCCGGGAGGAATTCCTGTCCGTGGTGCTGGATGTGGCTCTATCGTCCACACGGAGGAAG |
| **HPRT** | Cas12a_38087-AS | Cas12a | AAUUAUGGGGAUUACUAGGA | AAAGACTATGAAATGGAGAGCTAAATTATGGGGATTACTAGAATTCGGAAGGGGCAGCAATGAGTTGACACTACAGACAAGGCACT |
| | Cas12a_ HPRT 38330-AS | Cas12a | GGUUAAAGAUGGUUAAAUGAUUG | CATTTCACATAAAACTCTTTTAGGTTAAAGATGGTTAAgaattcATGATTGACAAAAAAAGTAATTCACTTACAGTCTGGCTTATA |

**Supplementary Table 7 Genomic targets used to compare the HDR efficiency mediated by SpCas9 and AsCas12a Ultra in Jurkat cells.**

| gRNA Target | Sequence Name | Sequence |
|---|---|---|
| B2M | B2M AsCpf1-1 | AGTGGGGGTGAATTCAGTGT |
| B2M | B2M AsCpf1-2 | CCGATATTCCTCAGGTACTC |
| B2M | B2M AsCpf1-3 | GAGTACCTGAGGAATATCGG |
| B2M | B2M AsCpf1-4 | CTCACGTCATCCAGCAGAGA |
| B2M | B2M AsCpf1-5 | CATTCTCTGCTGGATGACGT |
| B2M | B2M AsCpf1-6 | ACTTTCCATTCTCTGCTGGA |
| B2M | B2M AsCpf1-7 | CTGAATTGCTATGTGTCTGG |
| B2M | B2M AsCpf1-8 | ATCCATCCGACATTGAAGTT |
| B2M | B2M AsCpf1-9 | AATTCTCTCTCCATTCTTCA |
| B2M | B2M AsCpf1-10 | AGCAAGGACTGGTCTTTCTA |
| B2M | B2M AsCpf1-11 | TATCTCTTGTACTACACTGA |
| B2M | B2M AsCpf1-12 | TCACAGCCCAAGATAGTTAA |
| BCL11A | BCL11A AsCpf1-1 | CCCAGGGGGGCCTCTTTCGG |
| BCL11A | BCL11A AsCpf1-2 | TCACAGGCTCCAGGAAGGGT |
| BCL11A | BCL11A AsCpf1-3 | AAGCTAGTCTAGTGCAAGCT |
| BCL11A | BCL11A AsCpf1-4 | CACTGGAATCAGCTATCTGC |
| BCL11A | BCL11A AsCpf1-5 | AGCCATCTCACTACAGATAA |
| BCL11A | BCL11A AsCpf1-6 | CCCATGGGGCACAGTCAGGC |
| TGFBR2 | TGFBR2 AsCpf1-1 | TGATGTGAGATTTTCCACCT |
| PD1 | PD1 AsCpf1-1 | GCACGAAGCTCTCCGATGTG |
| AAVS1 | AAVS1 AsCpf1-1 | GACCCCTAAGGGAAGAATGA |
| CIITA | CIITA AsCpf1-1 | CCGGCCTTTTTACCTTGGGG |
| TRAC | TRAC AsCpf1-1 | CAGATACGAACCTAAACTTT |
| TRAC | TRAC AsCpf1-2 | GAGTCTCTCAGCTGGTACAC |
| CISH | CISH AsCpf1-1 | ACTGACAGCGTGAACAGGTA |
| HBB | HBB AsCpf1-1 | TATTGGTCTCCTTAAACCTGT |
| HBB | HBB AsCpf1-2 | AGGAGACCAATAGAAACTGGG |
| HBB | HBB AsCpf1-3 | AGGTTGCTAGTGAACACAGTT |
| HBB | HBB AsCpf1-4 | CTTCTGACACAACTGTGTTCA |

**Supplementary Table 8 List of crRNA targets used in Primary cells.**

| Assay | gBlock fragment |
|---|---|
| DNMT1 | GAGGGCCTATTTCCCATGATTCCTTCATATTTGCATATACGATACAAGGCTGTTAGAGAGATAATTGGAATTAATTTGACTGTAAACACAAAGATATTAGTACAAAATACGTGACGTAGAAAGTAAT<br>AATTTCTTGGGTAGTTTGCAGTTTTAAAATTATGTTTTAAAATGGACTATCATATGCTTACCGTAACTTGAAAGTATTTCGATTTCTTGGCTTTATATATCTTGTGGAAAGGACGAAACACCGTAAT<br>TTCTACTCTTGTAGATCTGATGGTCCATGTCTGTTACTC |
| STAT3 | GAGGGCCTATTTCCCATGATTCCTTCATATTTGCATATACGATACAAGGCTGTTAGAGAGATAATTGGAATTAATTTGACTGTAAACACAAAGATATTAGTACAAAATACGTGACGTAGAAAGTAAT<br>AATTTCTTGGGTAGTTTGCAGTTTTAAAATTATGTTTTAAAATGGACTATCATATGCTTACCGTAACTTGAAAGTATTTCGATTTCTTGGCTTTATATATCTTGTGGAAAGGACGAAACACCGTAAT<br>TTCTACTCTTGTAGATCAAAAACAAGCAGCAGGCTGGAT |
| GCK | GAGGGCCTATTTCCCATGATTCCTTCATATTTGCATATACGATACAAGGCTGTTAGAGAGATAATTGGAATTAATTTGACTGTAAACACAAAGATATTAGTACAAAATACGTGACGTAGAAAGTAAT<br>AATTTCTTGGGTAGTTTGCAGTTTTAAAATTATGTTTTAAAATGGACTATCATATGCTTACCGTAACTTGAAAGTATTTCGATTTCTTGGCTTTATATATCTTGTGGAAAGGACGAAACACCGTAAT<br>TTCTACTCTTGTAGATGACTCTGGTCCTGCGACCCGGAA |
| RAG2 | GAGGGCCTATTTCCCATGATTCCTTCATATTTGCATATACGATACAAGGCTGTTAGAGAGATAATTGGAATTAATTTGACTGTAAACACAAAGATATTAGTACAAAATACGTGACGTAGAAAGTAAT<br>AATTTCTTGGGTAGTTTGCAGTTTTAAAATTATGTTTTAAAATGGACTATCATATGCTTACCGTAACTTGAAAGTATTTCGATTTCTTGGCTTTATATATCTTGTGGAAAGGACGAAACACCGTAAT<br>TTCTACTCTTGTAGATTTGTTCTTGCAAACAATAGACAT |
| HPRT | GAGGGCCTATTTCCCATGATTCCTTCATATTTGCATATACGATACAAGGCTGTTAGAGAGATAATTGGAATTAATTTGACTGTAAACACAAAGATATTAGTACAAAATACGTGACGTAGAAAGTAAT<br>AATTTCTTGGGTAGTTTGCAGTTTTAAAATTATGTTTTAAAATGGACTATCATATGCTTACCGTAACTTGAAAGTATTTCGATTTCTTGGCTTTATATATCTTGTGGAAAGGACGAAACACCGTAAT<br>TTCTACTCTTGTAGATGGAAAGAGAATTGTTTTCTCCTT |
| GYS2 | GAGGGCCTATTTCCCATGATTCCTTCATATTTGCATATACGATACAAGGCTGTTAGAGAGATAATTGGAATTAATTTGACTGTAAACACAAAGATATTAGTACAAAATACGTGACGTAGAAAGTAAT<br>AATTTCTTGGGTAGTTTGCAGTTTTAAAATTATGTTTTAAAATGGACTATCATATGCTTACCGTAACTTGAAAGTATTTCGATTTCTTGGCTTTATATATCTTGTGGAAAGGACGAAACACCGTAAT<br>TTCTACTCTTGTAGATTCGTCTTTCTGGGCAGGTATTGT |

**Supplementary Table 9 gBlock dsDNA fragments used to express Cas12a crRNA for GUIDE-seq in HEK293 cells.**

| Name | Sequence |
| --- | --- |
| **pAAV_MND_E<br>GFRCAR_RSQ<br>7375_500HA** | cctgcaggcagctgcgcgctcgctcgctcactgaggccgcccgggcaaagcccgggcgtcgggcgacctttggtcgcccggcctcagtgagcgagcgagcgcgcagagagggagtggcc<br>aactccatcactaggggttcctgcggccgcggttcctcagatctgtaatgccaacataccataaacctcccattctgctaatgcccagcctaagttggggagaccactccagattccaa<br>gatgtacagtttgctttgctgggcctttttcccatgcctgcctttactctgccagagttatattgctggggttttgaagaagatcctattaaataaaagaataagcagtattattaagt<br>agccctgcatttcaggtttccttgagtggcaggccaggcctggccgtgaacgttcactgaaatcatggcctcttggccaagattgatagcttgtgcctgtccctgagtcccagtccatc<br>acgagcagctggtttctaagatgctatttcccgtataaagcatgagaccgtgacttgccagccccacagagccccgcccttgtccatcactggcatctggactccagcctgggttgggg<br>caaagagggaaatgagatcatgtcctaaccctgatcctcttgtcccacagatatccagaaccctgacccaatgaaagaccccacctgtaggtttggcaagctaggatcaaggttaggaa<br>cagagagacagcagaatatgggccaaacaggatatctgtggtaagcagttcctgccccggctcagggccaagaacagttggaacagcagaatatgggccaaacaggatatctgtggtaa<br>gcagttcctgccccggctcagggccaagaacagatggtccccagatgcggtcccgccctcagcagtttctagagaaccatcagatgtttccagggtgccccaaggacctgaaatgaccc<br>tgtgccttatttgaactaaccaatcagttcgcttctcgcttctgttcgcgcgcttctgctccccgagctcaataaaagagcccacaacccctcactcggcccggtcgccaccatggcac<br>tccccgtcaccgcccttctcttgcccctcgccctgctgctgcatgctgccaggcccatggacgaagtgcagctcgtggagtccggtggaggactcgtccaaccgggcggatcccttcgc<br>ttgtcctgcgccgcatcaggcttcagcttcaccaactatggcgtccactgggtcagacaggcccccggaaagggactggaatgggtgtccgtgatctggagcggcgggaacaccgacta<br>caacacctccgtgaagggccggttcactattagccgcgacaactccaagaacactctgtacctccaaatgaactccctgagggccgaagatactgctgtgtactattgcgcgagagccc<br>tgacctactacgactacgagttcgcgtactggggccaggggactctcgtgaccgtgtccagcggtggtggaggttccggaggcggaggttctggtggcgggggatcagaaatcgtgctg<br>actcagtcccctgcgaccttgtccctgagccctggagaacgggccaccctgagctgtagagccagccagagcatcgggacaaatattcactggtaccagcagaaacccggacaagcacc<br>acggctgctgatctactacgcctccgagtcgatttccggaatcccggctcgcttttcggggtctggatcgggaacggacttcactctgaccatctcgtcgctggaacccgaggatttcg<br>ccgtgtactactgccaacagaacaacaattggccgaccacgttcggccagggcaccaagctcgagattaagggatcactggaagcggccgcaaccacaacacctgctccaaggcccccc<br>acacccgctccaactatagccagccaaccattgagcctcagacctgaagcttgcaggcccgcagcaggaggcgccgtccatacgcgaggcctggacttcgcgtgtgatatttatatttg<br>ggcccctttggccggaacatgtggggtgttgcttctctcccttgtgatcactctgtattgtaagcgcgggagaaagaagctcctgtacatcttcaagcagccttttatgcgacctgtgc<br>aaaccactcaggaagaagatgggtgttcatgccgcttccccgaggaggaagaaggagggtgtgaactgagggtgaaattttctagaagcgccgatgctcccgcatatcagcagggtcag<br>aatcagctctacaatgaattgaatctcggcaggcgagaagagtacgatgttctggacaagagacggggcagggatcccgagatgggggaaagccccggagaaaaaatcctcaggaggg<br>gttgtacaatgagctgcagaaggacaagatggctgaagcctatagcgagatcggaatgaaaggcgaaagacgcagaggcaaggggcatgacggtctgtaccagggtctctctacagcca<br>ccaaggacacttatgatgcgttgcatatgcaagccttgccaccccgctaaagcggccgcgtcgagtctagagggcccgtttaaacccgctgatcagcctcgactgtgccttctagttgc<br>cagccatctgttgtttgcccctcccccgtgccttccttgaccctggaaggtgccactcccactgtcctttcctaataaaatgaggaaattgcatcgcattgtctgagtaggtgtcattc<br>tattctggggggtggggtggggcaggacagcaagggggaggattgggaagacaatagcaggcatgctggggatgcggtgggctctatggtgccgtgtaccagctgagagactcttaatc<br>cagtgacaagtctgtctgcctattcaccgattttgattctcaaacaaatgtgtcacaaagtaaggattctgatgtgtatatcacagacaaaactgtgctagacatgaggtctatggact<br>tcaagagcaacagtgctgtggcctggagcaacaaatctgactttgcatgtgcaaacgccttcaacaacagcattattccagaagacaccttcttccccagcccaggtaagggcagcttt<br>ggtgccttcgcaggctgtttccttgcttcaggaatggccaggttctgcccagagctctggtcaatgatgtctaaaactcctctgattggtggtctcggccttatccattgccaccaaaa<br>ccctctttttactaagaaacagtgagccttgttctggcagtccagagaatgacacgggaaaaaagcagatgaagagaaggtggcaggagagggcacgtggcccagcctcagtcctgggg<br>agagatctgcggccgcaggaacccctagtgatggagttggccactccctctctgcgcgctcgctcgctcactgaggccgggcgaccaaaggtcgcccgacgcccgggctttgcccgggc<br>ggcctcagtgagcgagcgagcgcgcagctgcctgcaggggcgcctgatgcggtattttctccttacgcatctgtgcggtatttcacaccgcatacgtcaaagcaaccatagtacgcgcc<br>ctgtagcggcgcattaagcgcggcgggtgtggtggttacgcgcagcgtgaccgctacacttgccagcgccctagcgcccgctcctttcgctttcttcccttcctttctcgccacgttcg<br>ccggctttccccgtcaagctctaaatcgggggctccctttagggttccgatttagtgctttacggcacctcgaccccaaaaaacttgatttgggtgatggttcacgtagtgggccatcg<br>ccctgatagacggtttttcgccctttgacgttggagtccacgttctttaatagtggactcttgttccaaactggaacaacactcaaccctatctcgggctattcttttgatttataagg<br>gattttgccgatttcggcctattggttaaaaaatgagctgatttaacaaaaatttaacgcgaattttaacaaaatattaacgtttacaattttatggtgcactctcagtacaatctgct<br>ctgatgccgcatagttaagccagccccgacacccgccaacacccgctgacgcgccctgacgggcttgtctgctcccggcatccgcttacagacaagctgtgaccgtctccgggagctgc<br>atgtgtcagaggttttcaccgtcatcaccgaaacgcgcgagacgaaagggcctcgtgatacgcctatttttataggttaatgtcatgataataatggtttcttagacgtcaggtggcac<br>ttttcggggaaatgtgcgcggaacccctatttgtttatttttctaaatacattcaaatatgtatccgctcatgagacaataaccctgataaatgcttcaataatattgaaaaaggaaga<br>gtatgagccatattcaacgggaaacgtcttgctctaggccgcgattaaattccaacatggatgctgatttatatgggtataaatgggctcgcgataatgtcgggcaatcaggtgcgaca<br>atctatcgattgtatgggaagcccgatgcgccagagttgtttctgaaacatggcaaaggtagcgttgccaatgatgttacagatgagatggtcagactaaactggctgacggaatttat<br>gcctcttccgaccatcaagcattttatccgtactcctgatgatgcatggttactcaccactgcgatccctgggaaaacagcattccaggtattagaagaatatcctgattcaggtgaaa<br>atattgttgatgcgctggcagtgttcctgcgccggttgcattcgattcctgtttgtaattgtccttttaacagcgatcgcgtatttcgtctcgctcaggcgcaatcacgaatgaataac<br>ggtttggttgatgcgagtgattttgatgacgagcgtaatggctggcctgttgaacaagtctggaaagaaatgcataaacttttgccattctcaccggattcagtcgtcactcatggtga<br>tttctcacttgataaccttatttttgacgaggggaaattaataggttgtattgatgttggacgagtcggaatcgcagaccgataccaggatcttgccatcctatggaactgcctcggtg<br>agttttctccttcattacagaaacggctttttcaaaaatatggtattgataatcctgatatgaataaattgcagtttcatttgatgctcgatgagtttttctaactgtcagaccaagtt<br>tactcatatatactttagattgatttaaaacttcatttttaatttaaaaggatctaggtgaagatcctttttgataatctcatgaccaaaatcccttaacgtgagttttcgttccactg<br>agcgtcagaccccgtagaaaagatcaaaggatcttcttgagatcctttttttctgcgcgtaatctgctgcttgcaaacaaaaaaaccaccgctaccagcggtggtttgtttgccggatc<br>aagagctaccaactctttttccgaaggtaactggcttcagcagagcgcagataccaaatactgtccttctagtgtagccgtagttaggccaccacttcaagaactctgtagcaccgcct<br>acatacctcgctctgctaatcctgttaccagtggctgctgccagtggcgataagtcgtgtcttaccgggttggactcaagacgatagttaccggataaggcgcagcggtcgggctgaac<br>ggggggttcgtgcacacagcccagcttggagcgaacgacctacaccgaactgagatacctacagcgtgagctatgagaaagcgccacgcttcccgaagggagaaaggcggacaggtatc |

| | |
|---|---|
| | cggtaagcggcagggtcggaacaggagagcgcacgagggagcttccagggggaaacgcctggtatctttatagtcctgtcgggtttcgccacctctgacttgagcgtcgatttttgtga<br>tgctcgtcaggggggcggagcctatggaaaaacgccagcaacgcggcctttttacggttcctggccttttgctggccttttgctcacatgt |
| **pAAV_PGK_GFP_RSQ7375_500HA** | gagagactcttaatccagtgacaagtctgtctgcctattcaccgattttgattctcaaacaaatgtgtcacaaagtaaggattctgatgtgtatatcacagacaaaactgtgctagaca<br>tgaggtctatggacttcaagagcaacagtgctgtggcctggagcaacaaatctgactttgcatgtgcaaacgccttcaacaacagcattattccagaagacaccttcttccccagccca<br>ggtaagggcagctttggtgccttcgcaggctgtttccttgcttcaggaatggccaggttctgcccagagctctggtcaatgatgtctaaaactcctctgattggtggtctcggccttat<br>ccattgccaccaaaaccctctttttactaagaaacagtgagccttgttctggcagtccagagaatgacacgggaaaaaagcagatgaagagaaggtggcaggagagggcacgtggccca<br>gcctcagtctctccaactgagttcctgcctgcctgcctttgctcagactgtttgccccttactgctcttctaggcctcattctaagccccttctccaagttgcctctccttatttctcc<br>ctgtctgccaaaaaatctttcccagctcactaagtcagtctcacgcagtcactcattaacccacctggggagagatctgcggccgcaggaacccctagtgatggagttggccactccct<br>ctctgcgcgctcgctcgctcactgaggccgggcgaccaaaggtcgcccgacgcccgggctttgcccgggcggcctcagtgagcgagcgagcgcgcagctgcctgcaggggcgcctgatg<br>cggtattttctccttacgcatctgtgcggtatttcacaccgcatacgtcaaagcaaccatagtacgcgccctgtagcggcgcattaagcgcggcgggtgtggtggttacgcgcagcgtg<br>accgctacacttgccagcgccctagcgcccgctcctttcgctttcttcccttcctttctcgccacgttcgccggctttccccgtcaagctctaaatcgggggctccctttagggttccg<br>atttagtgctttacggcacctcgaccccaaaaaacttgatttgggtgatggttcacgtagtgggccatcgccctgatagacggtttttcgccctttgacgttggagtccacgttcttta<br>atagtggactcttgttccaaactggaacaacactcaaccctatctcgggctattcttttgatttataagggattttgccgatttcggcctattggttaaaaaatgagctgatttaacaa<br>aaatttaacgcgaattttaacaaaatattaacgtttacaattttatggtgcactctcagtacaatctgctctgatgccgcatagttaagccagccccgacacccgccaacacccgctga<br>cgcgccctgacgggcttgtctgctcccggcatccgcttacagacaagctgtgaccgtctccgggagctgcatgtgtcagaggttttcaccgtcatcaccgaaacgcgcgagacgaaagg<br>gcctcgtgatacgcctatttttataggttaatgtcatgataataatggtttcttagacgtcaggtggcacttttcggggaaatgtgcgcggaacccctatttgtttatttttctaaata<br>cattcaaatatgtatccgctcatgagacaataaccctgataaatgcttcaataatattgaaaaaggaagagtatgagccatattcaacgggaaacgtcttgctctaggccgcgattaaa<br>ttccaacatggatgctgatttatatgggtataaatgggctcgcgataatgtcgggcaatcaggtgcgacaatctatcgattgtatgggaagcccgatgcgccagagttgtttctgaaac<br>atggcaaaggtagcgttgccaatgatgttacagatgagatggtcagactaaactggctgacggaatttatgcctcttccgaccatcaagcattttatccgtactcctgatgatgcatgg<br>ttactcaccactgcgatccctgggaaaacagcattccaggtattagaagaatatcctgattcaggtgaaaatattgttgatgcgctggcagtgttcctgcgccggttgcattcgattcc<br>tgtttgtaattgtccttttaacagcgatcgcgtatttcgtctcgctcaggcgcaatcacgaatgaataacggtttggttgatgcgagtgattttgatgacgagcgtaatggctggcctg<br>ttgaacaagtctggaaagaaatgcataaacttttgccattctcaccggattcagtcgtcactcatggtgatttctcacttgataaccttatttttgacgaggggaaattaataggttgt<br>attgatgttggacgagtcggaatcgcagaccgataccaggatcttgccatcctatggaactgcctcggtgagttttctccttcattacagaaacggctttttcaaaaatatggtattga<br>taatcctgatatgaataaattgcagtttcatttgatgctcgatgagtttttctaactgtcagaccaagtttactcatatatactttagattgatttaaaacttcatttttaatttaaaa<br>ggatctaggtgaagatcctttttgataatctcatgaccaaaatcccttaacgtgagttttcgttccactgagcgtcagaccccgtagaaaagatcaaaggatcttcttgagatcctttt<br>tttctgcgcgtaatctgctgcttgcaaacaaaaaaaccaccgctaccagcggtggtttgtttgccggatcaagagctaccaactctttttccgaaggtaactggcttcagcagagcgca<br>gataccaaatactgtccttctagtgtagccgtagttaggccaccacttcaagaactctgtagcaccgcctacatacctcgctctgctaatcctgttaccagtggctgctgccagtggcg<br>ataagtcgtgtcttaccgggttggactcaagacgatagttaccggataaggcgcagcggtcgggctgaacggggggttcgtgcacacagcccagcttggagcgaacgacctacaccgaa<br>ctgagatacctacagcgtgagctatgagaaagcgccacgcttcccgaagggagaaaggcggacaggtatccggtaagcggcagggtcggaacaggagagcgcacgagggagcttccagg<br>gggaaacgcctggtatctttatagtcctgtcgggtttcgccacctctgacttgagcgtcgatttttgtgatgctcgtcaggggggcggagcctatggaaaaacgccagcaacgcggcct<br>ttttacggttcctggccttttgctggccttttgctcacatgtcctgcaggcagctgcgcgctcgctcgctcactgaggccgcccgggcaaagcccgggcgtcgggcgacctttggtcgc<br>ccggcctcagtgagcgagcgagcgcgcagagagggagtggccaactccatcactaggggttcctgcggccgcggttcctcagatctggatgtaaggagctgctgtgacttgctcaaggc<br>cttatatcgagtaaacggtagcgctggggcttagacgcaggtgttctgatttatagttcaaaacctctatcaatgagagagcaatctcctggtaatgtgatagatttcccaacttaatg<br>ccaacataccataaacctcccattctgctaatgcccagcctaagttggggagaccactccagattccaagatgtacagtttgctttgctgggcctttttcccatgcctgcctttactct<br>gccagagttatattgctggggttttgaagaagatcctattaaataaaagaataagcagtattattaagtagccctgcatttcaggtttccttgagtggcaggccaggcctggccgtgaa<br>cgttcactgaaatcatggcctcttggccaagattgatagcttgtgcctgtccctgagtcccagtccatcacgagcagctggtttctaagatgctatttcccgtataaagcatgagaccg<br>tgacttgccagccccacagagccccgcccttgtccatcactggcatctggactccagcctgggttggggcaaagagggaaatgagatcatgtcctaaccctgatcctcttgtcccacag<br>atatccagaaccctgaccccacggggttggggttgcgccttttccaaggcagccctgggtttgcgcagggacgcggctgctctgggcgtggttccgggaaacgcagcggcgccgaccct<br>gggtctcgcacattcttcacgtccgttcgcagcgtcacccggatcttcgccgctacccttgtgggccccccggcgacgcttcctgctccgcccctaagtcgggaaggttccttgcggtt<br>cgcggcgtgccggacgtgacaaacggaagccgcacgtctcactagtaccctcgcagacggacagcgccagggagcaatggcagcgcgccgaccgcgatgggctgtggccaatagcggct<br>gctcagcagggcgcgccgagagcagcggccgggaaggggcggtgcgggaggcggggtgtggggcggtagtgtgggccctgttcctgcccgcgcggtgttccgcattctgcaagcctccg<br>gagcgcacgtcggcagtcggctccctcgttgaccgaatcaccgacctctctccccaccggtcgccaccatggtgagcaagggcgaggagctgttcaccggggtggtgcccatcctggtc<br>gagctggacggcgacgtaaacggccacaagttcagcgtgtccggcgagggcgagggcgatgccacctacggcaagctgaccctgaagttcatctgcaccaccggcaagctgcccgtgcc<br>ctggcccaccctcgtgaccaccctgacctacggcgtgcagtgcttcagccgctaccccgaccacatgaagcagcacgacttcttcaagtccgccatgcccgaaggctacgtccaggagc<br>gcaccatcttcttcaaggacgacggcaactacaagacccgcgccgaggtgaagttcgagggcgacaccctggtgaaccgcatcgagctgaagggcatcgacttcaaggaggacggcaac<br>atcctggggcacaagctggagtacaactacaacagccacaacgtctatatcatggccgacaagcagaagaacggcatcaaggtgaacttcaagatccgccacaacatcgaggacggcag<br>cgtgcagctcgccgaccactaccagcagaacacccccatcggcgacggccccgtgctgctgcccgacaaccactacctgagcacccagtccgccctgagcaaagaccccaacgagaagc<br>gcgatcacatggtcctgctggagttcgtgaccgccgccgggatcactctcggcatggacgagctgtacaagtaaagcggccgcgtcgagtctagagggcccgtttaaacccgctgatca<br>gcctcgactgtgccttctagttgccagccatctgttgtttgcccctcccccgtgccttccttgaccctggaaggtgccactcccactgtcctttcctaataaaatgaggaaattgcatc<br>gcattgtctgagtaggtgtcattctattctggggggtggggtggggcaggacagcaagggggaggattgggaagacaatagcaggcatgctggggatgcggtggctctatgg |

| | |
|---|---|
| **pAAV_PGK_GFP_RSQ7618_500HA** | ctggggagagatctgcggccgcaggaacccctagtgatggagttggccactccctctctgcgcgctcgctcgctcactgaggccgggcgaccaaaggtcgcccgacgcccgggctttgc<br>ccgggcggcctcagtgagcgagcgagcgcgcagctgcctgcaggggcgcctgatgcggtattttctccttacgcatctgtgcggtatttcacaccgcatacgtcaaagcaaccatagta<br>cgcgccctgtagcggcgcattaagcgcggcgggtgtggtggttacgcgcagcgtgaccgctacacttgccagcgccctagcgcccgctcctttcgctttcttcccttcctttctcgcca<br>cgttcgccggctttccccgtcaagctctaaatcgggggctccctttagggttccgatttagtgctttacggcacctcgaccccaaaaaacttgatttgggtgatggttcacgtagtggg<br>ccatcgccctgatagacggtttttcgccctttgacgttggagtccacgttctttaatagtggactcttgttccaaactggaacaacactcaaccctatctcgggctattcttttgattt<br>ataagggattttgccgatttcggcctattggttaaaaaatgagctgatttaacaaaaatttaacgcgaattttaacaaaatattaacgtttacaattttatggtgcactctcagtacaa<br>tctgctctgatgccgcatagttaagccagccccgacacccgccaacacccgctgacgcgccctgacgggcttgtctgctcccggcatccgcttacagacaagctgtgaccgtctccggg<br>agctgcatgtgtcagaggttttcaccgtcatcaccgaaacgcgcgagacgaaagggcctcgtgatacgcctatttttataggttaatgtcatgataataatggtttcttagacgtcagg<br>tggcacttttcggggaaatgtgcgcggaacccctatttgtttatttttctaaatacattcaaatatgtatccgctcatgagacaataaccctgataaatgcttcaataatattgaaaaa<br>ggaagagtatgagccatattcaacgggaaacgtcttgctctaggccgcgattaaattccaacatggatgctgatttatatgggtataaatgggctcgcgataatgtcgggcaatcaggt<br>gcgacaatctatcgattgtatgggaagcccgatgcgccagagttgtttctgaaacatggcaaaggtagcgttgccaatgatgttacagatgagatggtcagactaaactggctgacgga<br>atttatgcctcttccgaccatcaagcattttatccgtactcctgatgatgcatggttactcaccactgcgatccctgggaaaacagcattccaggtattagaagaatatcctgattcag<br>gtgaaaatattgttgatgcgctggcagtgttcctgcgccggttgcattcgattcctgtttgtaattgtccttttaacagcgatcgcgtatttcgtctcgctcaggcgcaatcacgaatg<br>aataacggtttggttgatgcgagtgattttgatgacgagcgtaatggctggcctgttgaacaagtctggaaagaaatgcataaacttttgccattctcaccggattcagtcgtcactca<br>tggtgatttctcacttgataaccttatttttgacgaggggaaattaataggttgtattgatgttggacgagtcggaatcgcagaccgataccaggatcttgccatcctatggaactgcc<br>tcggtgagttttctccttcattacagaaacggctttttcaaaaatatggtattgataatcctgatatgaataaattgcagtttcatttgatgctcgatgagtttttctaactgtcagac<br>caagtttactcatatatactttagattgatttaaaacttcatttttaatttaaaaggatctaggtgaagatcctttttgataatctcatgaccaaaatcccttaacgtgagttttcgtt<br>ccactgagcgtcagaccccgtagaaaagatcaaaggatcttcttgagatcctttttttctgcgcgtaatctgctgcttgcaaacaaaaaaaccaccgctaccagcggtggtttgtttgc<br>cggatcaagagctaccaactctttttccgaaggtaactggcttcagcagagcgcagataccaaatactgtccttctagtgtagccgtagttaggccaccacttcaagaactctgtagca<br>ccgcctacatacctcgctctgctaatcctgttaccagtggctgctgccagtggcgataagtcgtgtcttaccgggttggactcaagacgatagttaccggataaggcgcagcggtcggg<br>ctgaacggggggttcgtgcacacagcccagcttggagcgaacgacctacaccgaactgagatacctacagcgtgagctatgagaaagcgccacgcttcccgaagggagaaaggcggaca<br>ggtatccggtaagcggcagggtcggaacaggagagcgcacgagggagcttccagggggaaacgcctggtatctttatagtcctgtcgggtttcgccacctctgacttgagcgtcgattt<br>ttgtgatgctcgtcaggggggcggagcctatggaaaaacgccagcaacgcggcctttttacggttcctggccttttgctggccttttgctcacatgtcctgcaggcagctgcgcgctcg<br>ctcgctcactgaggccgcccgggcaaagcccgggcgtcgggcgacctttggtcgcccggcctcagtgagcgagcgagcgcgcagagagggagtggccaactccatcactaggggttcct<br>gcggccgcggttcctcagatctgttctttcaagacaagcaacagtactcacataggctgtgggcaatggtcctgtctctcaagaatcccctgccactcctcacacccaccctgggccca<br>tattcatttccatttgagttgttcttattgagtcatccttcctgtggcagcggaactcactaagggggcccatctggacccgaggtattgtgaagataaattctgagcacctaccccatc<br>cccagaagggctcagaaataaaataagagccaagtctagtcggtgtttcctgtcttgaaacacaatactgttggccctggaagaatgcacagaatctgtttgtaaggggatatgcacag<br>aagctgcaagggacaggaggtgcaggagctgcaggcctcccccacccagcctgctctgccttggggaaaaccgtgggtgtgtcctgcaggccatgcaggcctgggacatgcaagcccat<br>aaccgctgtggcctcttggtttaacagatacgaacctaaactttcaaccacggggttggggttgcgccttttccaaggcagccctgggtttgcgcagggacgcggctgctctgggcgtg<br>gttccgggaaacgcagcggcgccgaccctgggtctcgcacattcttcacgtccgttcgcagcgtcacccggatcttcgccgctacccttgtgggccccccggcgacgcttcctgctccg<br>cccctaagtcgggaaggttccttgcggttcgcggcgtgccggacgtgacaaacggaagccgcacgtctcactagtaccctcgcagacggacagcgccagggagcaatggcagcgcgccg<br>accgcgatgggctgtggccaatagcggctgctcagcagggcgcgccgagagcagcggccgggaaggggcggtgcgggaggcggggtgtggggcggtagtgtgggccctgttcctgcccg<br>cgcggtgttccgcattctgcaagcctccggagcgcacgtcggcagtcggctccctcgttgaccgaatcaccgacctctctccccaccggtcgccaccatggtgagcaagggcgaggagc<br>tgttcaccggggtggtgcccatcctggtcgagctggacggcgacgtaaacggccacaagttcagcgtgtccggcgagggcgagggcgatgccacctacggcaagctgaccctgaagttc<br>atctgcaccaccggcaagctgcccgtgccctggcccaccctcgtgaccaccctgacctacggcgtgcagtgcttcagccgctaccccgaccacatgaagcagcacgacttcttcaagtc<br>cgccatgcccgaaggctacgtccaggagcgcaccatcttcttcaaggacgacggcaactacaagacccgcgccgaggtgaagttcgagggcgacaccctggtgaaccgcatcgagctga<br>agggcatcgacttcaaggaggacggcaacatcctggggcacaagctggagtacaactacaacagccacaacgtctatatcatggccgacaagcagaagaacggcatcaaggtgaacttc<br>aagatccgccacaacatcgaggacggcagcgtgcagctcgccgaccactaccagcagaacacccccatcggcgacggccccgtgctgctgcccgacaaccactacctgagcacccagtc<br>cgccctgagcaaagaccccaacgagaagcgcgatcacatggtcctgctggagttcgtgaccgccgccgggatcactctcggcatggacgagctgtacaagtaaagcggccgcgtcgagt<br>ctagagggcccgtttaaacccgctgatcagcctcgactgtgccttctagttgccagccatctgttgtttgcccctcccccgtgccttccttgaccctggaaggtgccactcccactgtc<br>ctttcctaataaaatgaggaaattgcatcgcattgtctgagtaggtgtcattctattctggggggtggggtggggcaggacagcaagggggaggattgggaagacaatagcaggcatgc<br>tggggatgcggtgggctctatggtcaaaacctgtcagtgattgggttccgaatcctcctcctgaaagtggccgggtttaatctgctcatgacgctgcggctgtggtccagctgaggtga<br>ggggccttgaagctgggagtggggtttagggacgcgggtctctgcgtgcatcctaagctctgagagcaaacctccctgcagggtcttgcttttaagtccaaagcctgagcccaccaaac<br>tctcctacttcttcctgttacaaattcctcttgtgcaataataatggcctgaaacgctgtaaaatatcctcatttcagccgcctcagttgaacttctcccctatgaggtaggaagaaca<br>gttgtttagaaacgaagaaactgaggccacacagctaatgagtggaggaagagagacacttgtgtacaccacatgccttgtgttgtacttctctcaccgtgtaacctcctcatgtcctc<br>tctccccagtacggctctcttagctcagtagaaagaagacattacac |
| **pAAV_PGK_mCherry_RSQ8443_500HA** | aaatgcttcaataatattgaaaaaggaagagtatgagccatattcaacgggaaacgtcttgctctaggccgcgattaaattccaacatggatgctgatttatatgggtataaatgggct<br>cgcgataatgtcgggcaatcaggtgcgacaatctatcgattgtatgggaagcccgatgcgccagagttgtttctgaaacatggcaaaggtagcgttgccaatgatgttacagatgagat<br>ggtcagactaaactggctgacggaatttatgcctcttccgaccatcaagcattttatccgtactcctgatgatgcatggttactcaccactgcgatccctgggaaaacagcattccagg<br>tattagaagaatatcctgattcaggtgaaaatattgttgatgcgctggcagtgttcctgcgccggttgcattcgattcctgtttgtaattgtccttttaacagcgatcgcgtatttcgt<br>ctcgctcaggcgcaatcacgaatgaataacggtttggttgatgcgagtgattttgatgacgagcgtaatggctggcctgttgaacaagtctggaaagaaatgcataaacttttgccatt |

ctcaccggattcagtcgtcactcatggtgatttctcacttgataaccttatttttgacgaggggaaattaataggttgtattgatgttggacgagtcggaatcgcagaccgataccagg
atcttgccatcctatggaactgcctcggtgagttttctccttcattacagaaacggctttttcaaaaatatggtattgataatcctgatatgaataaattgcagtttcatttgatgctc
gatgagtttttctaactgtcagaccaagtttactcatatatactttagattgatttaaaacttcatttttaatttaaaaggatctaggtgaagatcctttttgataatctcatgaccaa
aatcccttaacgtgagttttcgttccactgagcgtcagaccccgtagaaaagatcaaaggatcttcttgagatcctttttttctgcgcgtaatctgctgcttgcaaacaaaaaaaccac
cgctaccagcggtggtttgtttgccggatcaagagctaccaactctttttccgaaggtaactggcttcagcagagcgcagataccaaatactgtccttctagtgtagccgtagttaggc
caccacttcaagaactctgtagcaccgcctacatacctcgctctgctaatcctgttaccagtggctgctgccagtggcgataagtcgtgtcttaccgggttggactcaagacgatagtt
accggataaggcgcagcggtcgggctgaacggggggttcgtgcacacagcccagcttggagcgaacgacctacaccgaactgagatacctacagcgtgagctatgagaaagcgccacgc
ttcccgaagggagaaaggcggacaggtatccggtaagcggcagggtcggaacaggagagcgcacgagggagcttccagggggaaacgcctggtatctttatagtcctgtcgggtttcgc
cacctctgacttgagcgtcgatttttgtgatgctcgtcaggggggcggagcctatggaaaaacgccagcaacgcggcctttttacggttcctggccttttgctggccttttgctcacat
gtcctgcaggcagctgcgcgctcgctcgctcactgaggccgcccgggcaaagcccgggcgtcgggcgacctttggtcgcccggcctcagtgagcgagcgagcgcgcagagagggagtgg
ccaactccatcactaggggttcctgcggccgcggttcctcagatctggaaaggctcttaaaaatgcagcgcaatctccagtgacagaagatactgctagaaatctgctagaaaaaaaac
aaaaaaggcatgtatagaggaattatgagggaaagataccaagtcacggtttattcttcaaaatggaggtggcttgttgggaaggtggaagctcatttggccagagtggaaatggaatt
gggagaaatcgatgaccaaatgtaaacacttggtgcctgatatagcttgacaccaagttagccccaagtgaaataccctggcaatattaatgtgtcttttcccgatattcctcaggtac
tccaaagattcaggtttactcacgtcatccagcagagaatggaaagtcaaatttcctgaattgctatgtgtctgggtttcatccatccgacattgaagttgacttactgaagaatggag
agagaattgaaaaagtggagcattcagacttgtctttcagcaaggactggtctttctatctcttgtactacccacggggttggggttgcgccttttccaaggcagccctgggtttgcgc
agggacgcggctgctctgggcgtggttccgggaaacgcagcggcgccgaccctgggtctcgcacattcttcacgtccgttcgcagcgtcacccggatcttcgccgctacccttgtgggc
cccccggcgacgcttcctgctccgcccctaagtcgggaaggttccttgcggttcgcggcgtgccggacgtgacaaacggaagccgcacgtctcactagtaccctcgcagacggacagcg
ccagggagcaatggcagcgcgccgaccgcgatgggctgtggccaatagcggctgctcagcagggcgcgccgagagcagcggccgggaaggggcggtgcgggaggcggggtgtggggcgg
tagtgtgggccctgttcctgcccgcgcggtgttccgcattctgcaagcctccggagcgcacgtcggcagtcggctccctcgttgaccgaatcaccgacctctctccccactccccaccg
gtcgccaccatggtgagcaagggcgaggaggataacatggccatcatcaaggagttcatgcgcttcaaggtgcacatggagggctccgtgaacggccacgagttcgagatcgagggcga
gggcgagggccgcccctacgagggcacccagaccgccaagctgaaggtgaccaagggtggcccccctgcccttcgcctgggacatcctgtcccctcagttcatgtacggctccaaggcct
acgtgaagcaccccgccgacatccccgactacttgaagctgtccttccccgagggcttcaagtgggagcgcgtgatgaacttcgaggacggcggcgtggtgaccgtgacccaggactcc
tccctgcaggacggcgagttcatctacaaggtgaagctgcgcggcaccaacttcccctccgacggccccgtaatgcagaagaagacaatgggctgggaggcctcctccgagcggatgta
ccccgaggacggcgccctgaagggcgagatcaagcagaggctgaagctgaaggacggcggccactacgacgctgaggtcaagaccacctacaaggccaagaagcccgtgcagctgcccg
gcgcctacaacgtcaacatcaagttggacatcacctcccacaacgaggactacaccatcgtggaacagtacgaacgcgccgagggccgccactccaccggcggcatggacgagctgtac
aagtaagcggccgcgtcgagtctagagggcccgtttaaacccgctgatcagcctcgactgtgccttctagttgccagccatctgttgtttgcccctcccccgtgccttccttgaccctg
gaaggtgccactcccactgtcctttcctaataaaatgaggaaattgcatcgcattgtctgagtaggtgtcattctattctggggggtggggtggggcaggacagcaagggggaggattg
ggaagacaatagcaggcatgctggggatgcggtgggctctatggccccactgtaaaagatgagtatgcctgccgtgtgaaccatgtgactttgtcacagcccaagatagttaagtgggg
taagtcttacattcttttgtaagctgctgaaagttgtgtatgagtagtcatatcataaagctgctttgatataaaaaaggtctatggccatactaccctgaatgagtcccatcccatct
gatataaacaatctgcatattgggattgtcagggaatgttcttaaagatcagattagtggcacctgctgagatactgatgcacagcatggtttctgaaccagtagtttccctgcagttg
agcagggagcagcagcagcacttgcacaaatacatatacactcttaacacttcttacctactggcttcctctagcttttgtggcagcttcaggtatatttagcactgaacgaacatctc
aagaaggtataggcctttgtttgtaagtcctgctgtcctagcatcctataatcctggacttctccagtctggggagagatctgcggccgcaggaacccctagtgatggagttggccact
ccctctctgcgcgctcgctcgctcactgaggccgggcgaccaaaggtcgcccgacgcccgggctttgcccgggcggcctcagtgagcgagcgagcgcgcagctgcctgcaggggcgcct
gatgcggtattttctccttacgcatctgtgcggtatttcacaccgcatacgtcaaagcaaccatagtacgcgccctgtagcggcgcattaagcgcggcgggtgtggtggttacgcgcag
cgtgaccgctacacttgccagcgccctagcgcccgctcctttcgctttcttcccttcctttctcgccacgttcgccggctttccccgtcaagctctaaatcgggggctccctttagggt
tccgatttagtgctttacggcacctcgaccccaaaaaacttgatttgggtgatggttcacgtagtgggccatcgccctgatagacggtttttcgccctttgacgttggagtccacgttc
tttaatagtggactcttgttccaaactggaacaacactcaaccctatctcgggctattcttttgatttataagggattttgccgatttcggcctattggttaaaaaatgagctgattta
acaaaaatttaacgcgaattttaacaaaatattaacgtttacaattttatggtgcactctcagtacaatctgctctgatgccgcatagttaagccagccccgacacccgccaacacccg
ctgacgcgccctgacgggcttgtctgctcccggcatccgcttacagacaagctgtgaccgtctccgggagctgcatgtgtcagaggttttcaccgtcatcaccgaaacgcgcgagacga
aagggcctcgtgatacgcctatttttataggttaatgtcatgataataatggtttcttagacgtcaggtggcacttttcggggaaatgtgcgcggaacccctatttgtttatttttcta
aatacattcaaatatgtatccgctcatgagacaataaccctgat

**Supplementary Table 10 Plasmid sequences used to generate AAV6 as donor template.**

| Name | Sequence |
|---|---|
| **WT AsCas12a** | MTQFEGFTNLYQVSKTLRFELIPQGKTLKHIQEQGFIEEDKARNDHYKELKPIIDRIYKTYADQCLQLVQLDWENLSAAIDSYRKEKTEETRNALIEEQATYRNAIHDYFIGRTDNLTDAINKRH<br>AEIYKGLFKAELFNGKVLKQLGTVTTTEHENALLRSFDKFTTYFSGFYENRKNVFSAEDISTAIPHRIVQDNFPKFKENCHIFTRLITAVPSLREHFENVKKAIGIFVSTSIEEVFSFPFYNQLL<br>TQTQIDLYNQLLGGISREAGTEKIKGLNEVLNLAIQKNDETAHIIASLPHRFIPLFKQILSDRNTLSFILEEFKSDEEVIQSFCKYKTLLRNENVLETAEALFNELNSIDLTHIFISHKKLETIS<br>SALCDHWDTLRNALYERRISELTGKITKSAKEKVQRSLKHEDINLQEIISAAGKELSEAFKQKTSEILSHAHAALDQPLPTTLKKQEEKEILKSQLDSLLGLYHLLDWFAVDESNEVDPEFSARL<br>TGIKLEMEPSLSFYNKARNYATKKPYSVEKFKLNFQMPTLASGWDVNKEKNNGAILFVKNGLYYLGIMPKQKGRYKALSFEPTEKTSEGFDKMYYDYFPDAAKMIPKCSTQLKAVTAHFQTHTTP<br>ILLSNNFIEPLEITKEIYDLNNPEKEPKKFQTAYAKKTGDQKGYREALCKWIDFTRDFLSKYTKTTSIDLSSLRPSSQYKDLGEYYAELNPLLYHISFQRIAEKEIMDAVETGKLYLFQIYNKDF<br>AKGHHGKPNLHTLYWTGLFSPENLAKTSIKLNGQAELFYRPKSRMKRMAHRLGEKMLNKKLKDQKTPIPDTLYQELYDYVNHRLSHDLSDEARALLPNVITKEVSHEIIKDRRFTSDKFFFHVPI<br>TLNYQAANSPSKFNQRVNAYLKEHPETPIIGIDRGERNLIYITVIDSTGKILEQRSLNTIQQFDYQKKLDNREKERVAARQAWSVVGTIKDLKQGYLSQVIHEIVDLMIHYQAVVVLENLNFGFK<br>SKRTGIAEKAVYQQFEKMLIDKLNCLVLKDYPAEKVGGVLNPYQLTDQFTSFAKMGTQSGFLFYVPAPYTSKIDPLTGFVDPFVWKTIKNHESRKHFLEGFDFLHYDVKTGDFILHFKMNRNLSF<br>QRGLPGFMPAWDIVFEKNETQFDAKGTPFIAGKRIVPVIENHRFTGRYRDLYPANELIALLEEKGIVFRDGSNILPKLLENDDSHAIDTMVALIRSVLQMRNSNAATGEDYINSPVRDLNGVCFD<br>SRFQNPEWPMDADANGAYHIALKGQLLLNHLKESKDLKLQNGISNQDWLAYIQELRNGRSSDDEATADSQHAAPPKKKRKVGGSGGSGGSGGSGGSGGSGGSGGSLEHHHHHH |
| **AsCas 12 Ultra** | MTQFEGFTNLYQVSKTLRFELIPQGKTLKHIQEQGFIEEDKARNDHYKELKPIIDRIYKTYADQCLQLVQLDWENLSAAIDSYRKEKTEETRNALIEEQATYRNAIHDYFIGRTDNLTDAINKRH<br>AEIYKGLFKAELFNGKVLKQLGTVTTTEHENALLRSFDKFTTYFSGFYENRKNVFSAEDISTAIPHRIVQDNFPKFKENCHIFTRLITAVPSLREHFENVKKAIGIFVSTSIEEVFSFPFYNQLL<br>TQTQIDLYNQLLGGISREAGTEKIKGLNEVLNLAIQKNDETAHIIASLPHRFIPLFKQILSDRNTLSFILEEFKSDEEVIQSFCKYKTLLRNENVLETAEALFNELNSIDLTHIFISHKKLETIS<br>SALCDHWDTLRNALYERRISELTGKITKSAKEKVQRSLKHEDINLQEIISAAGKELSEAFKQKTSEILSHAHAALDQPLPTTLKKQEEKEILKSQLDSLLGLYHLLDWFAVDESNEVDPEFSARL<br>TGIKLEMEPSLSFYNKARNYATKKPYSVEKFKLNFQRPTLASGWDVNKEKNNGAILFVKNGLYYLGIMPKQKGRYKALSFEPTEKTSEGFDKMYYDYFPDAAKMIPKCSTQLKAVTAHFQTHTTP<br>ILLSNNFIEPLEITKEIYDLNNPEKEPKKFQTAYAKKTGDQKGYREALCKWIDFTRDFLSKYTKTTSIDLSSLRPSSQYKDLGEYYAELNPLLYHISFQRIAEKEIMDAVETGKLYLFQIYNKDF<br>AKGHHGKPNLHTLYWTGLFSPENLAKTSIKLNGQAELFYRPKSRMKRMAHRLGEKMLNKKLKDQKTPIPDTLYQELYDYVNHRLSHDLSDEARALLPNVITKEVSHEIIKDRRFTSDKFLFHVPI<br>TLNYQAANSPSKFNQRVNAYLKEHPETPIIGIDRGERNLIYITVIDSTGKILEQRSLNTIQQFDYQKKLDNREKERVAARQAWSVVGTIKDLKQGYLSQVIHEIVDLMIHYQAVVVLENLNFGFK<br>SKRTGIAEKAVYQQFEKMLIDKLNCLVLKDYPAEKVGGVLNPYQLTDQFTSFAKMGTQSGFLFYVPAPYTSKIDPLTGFVDPFVWKTIKNHESRKHFLEGFDFLHYDVKTGDFILHFKMNRNLSF<br>QRGLPGFMPAWDIVFEKNETQFDAKGTPFIAGKRIVPVIENHRFTGRYRDLYPANELIALLEEKGIVFRDGSNILPKLLENDDSHAIDTMVALIRSVLQMRNSNAATGEDYINSPVRDLNGVCFD<br>SRFQNPEWPMDADANGAYHIALKGQLLLNHLKESKDLKLQNGISNQDWLAYIQELRNGRSSDDEATADSQHAAPPKKKRKVGGSGGSGGSGGSGGSGGSGGSGGSLEHHHHHH |
| **WT LbCas 12a** | MSKLEKFTNCYSLSKTLRFKAIPVGKTQENIDNKRLLVEDEKRAEDYKGVKKLLDRYYLSFINDVLHSIKLKNLNNYISLFRKKTRTEKENKELENLEINLRKEIAKAFKGNEGYKSLFKKDIIE<br>TILPEFLDDKDEIALVNSFNGFTTAFTGFFDNRENMFSEEAKSTSIAFRCINENLTRYISNMDIFEKVDAIFDKHEVQEIKEKILNSDYDVEDFFEGEFFNFVLTQEGIDVYNAIIGGFVTESGE<br>KIKGLNEYINLYNQKTKQKLPKFKPLYKQVLSDRESLSFYGEGYTSDEEVLEVFRNTLNKNSEIFSSIKKLEKLFKNFDEYSSAGIFVKNGPAISTISKDIFGEWNVIRDKWNAEYDDIHLKKKA<br>VVTEKYEDDRRKSFKKIGSFSLEQLQEYADADLSVVEKLKEIIIQKVDEIYKVYGSSEKLFDADFVLEKSLKKNDAVVAIMKDLLDSVKSFENYIKAFFGEGKETNRDESFYGDFVLAYDILLKV<br>DHIYDAIRNYVTQKPYSKDKFKLYFQNPQFMGGWDKDKETDYRATILRYGSKYYLAIMDKKYAKCLQKIDKDDVNGNYEKINYKLLPGPNKMLPKVFFSKKWMAYYNPSEDIQKIYKNGTFKKGD<br>MFNLNDCHKLIDFFKDSISRYPKWSNAYDFNFSETEKYKDIAGFYREVEEQGYKVSFESASKKEVDKLVEEGKLYMFQIYNKDFSDKSHGTPNLHTMYFKLLFDENNHGQIRLSGGAELFMRRAS<br>LKKEELVVHPANSPIANKNPDNPKKTTTLSYDVYKDKRFSEDQYELHIPIAINKCPKNIFKINTEVRVLLKHDDNPYVIGIDRGERNLLYIVVVDGKGNIVEQYSLNEIINNFNGIRIKTDYHSL<br>LDKKEKERFEARQNWTSIENIKELKAGYISQVVHKICELVEKYDAVIALEDLNSGFKNSRVKVEKQVYQKFEKMLIDKLNYMVDKKSNPCATGGALKGYQITNKFESFKSMSTQNGFIFYIPAWL<br>TSKIDPSTGFVNLLKTKYTSIADSKKFISSFDRIMYVPEEDLFEFALDYKNFSRTDADYIKKWKLYSYGNRIRIFRNPKKNNVFDWEEVCLTSAYKELFNKYGINYQQGDIRALLCEQSDKAFYS<br>SFMALMSLMLQMRNSITGRTDVDFLISPVKNSDGIFYDSRNYEAQENAILPKNADANGAYNIARKVLWAIGQFKKAEDEKLDKVKIAISNKEWLEYAQTSVKHGRSSDDEATADSQHAAPPKKKR<br>KVGGSGGSGGSGGSGGSGGSGGSGGSLEHHHHHH |
| **LbCas 12a Ultra** | MSKLEKFTNCYSLSKTLRFKAIPVGKTQENIDNKRLLVEDEKRAEDYKGVKKLLDRYYLSFINDVLHSIKLKNLNNYISLFRKKTRTEKENKELENLEINLRKEIAKAFKGNEGYKSLFKKDIIE<br>TILPEFLDDKDEIALVNSFNGFTTAFTGFFDNRENMFSEEAKSTSIAFRCINENLTRYISNMDIFEKVDAIFDKHEVQEIKEKILNSDYDVEDFFEGEFFNFVLTQEGIDVYNAIIGGFVTESGE<br>KIKGLNEYINLYNQKTKQKLPKFKPLYKQVLSDRESLSFYGEGYTSDEEVLEVFRNTLNKNSEIFSSIKKLEKLFKNFDEYSSAGIFVKNGPAISTISKDIFGEWNVIRDKWNAEYDDIHLKKKA<br>VVTEKYEDDRRKSFKKIGSFSLEQLQEYADADLSVVEKLKEIIIQKVDEIYKVYGSSEKLFDADFVLEKSLKKNDAVVAIMKDLLDSVKSFENYIKAFFGEGKETNRDESFYGDFVLAYDILLKV<br>DHIYDAIRNYVTQKPYSKDKFKLYFQNPQFMGGWDKDKETDYRATILRYGSKYYLAIMDKKYAKCLQKIDKDDVNGNYEKINYKLLPGPNKMLPKVFFSKKWMAYYNPSEDIQKIYKNGTFKKGD<br>MFNLNDCHKLIDFFKDSISRYPKWSNAYDFNFSETEKYKDIAGFYREVEEQGYKVSFESASKKEVDKLVEEGKLYMFQIYNKDFSDKSHGTPNLHTMYFKLLFDENNHGQIRLSGGAELFMRRAS<br>LKKEELVVHPANSPIANKNPDNPKKTTTLSYDVYKDKRFSEDQYLLHIPIAINKCPKNIFKINTEVRVLLKHDDNPYVIGIDRGERNLLYIVVVDGKGNIVEQYSLNEIINNFNGIRIKTDYHSL<br>LDKKEKERFEARQNWTSIENIKELKAGYISQVVHKICELVEKYDAVIALEDLNSGFKNSRVKVEKQVYQKFEKMLIDKLNYMVDKKSNPCATGGALKGYQITNKFESFKSMSTQNGFIFYIPAWL<br>TSKIDPSTGFVNLLKTKYTSIADSKKFISSFDRIMYVPEEDLFEFALDYKNFSRTDADYIKKWKLYSYGNRIRIFRNPKKNNVFDWEEVCLTSAYKELFNKYGINYQQGDIRALLCEQSDKAFYS<br>SFMALMSLMLQMRNSITGRTDVDFLISPVKNSDGIFYDSRNYEAQENAILPKNADANGAYNIARKVLWAIGQFKKAEDEKLDKVKIAISNKEWLEYAQTSVKHGRSSDDEATADSQHAAPPKKKR<br>KVGGSGGSGGSGGSGGSGGSGGSGGSLEHHHHHH |
| **enCas 12a** | MTQFEGFTNLYQVSKTLRFELIPQGKTLKHIQEQGFIEEDKARNDHYKELKPIIDRIYKTYADQCLQLVQLDWENLSAAIDSYRKEKTEETRNALIEEQATYRNAIHDYFIGRTDNLTDAINKRH<br>AEIYKGLFKAELFNGKVLKQLGTVTTTEHENALLRSFDKFTTYFSGFYRNRKNVFSAEDISTAIPHRIVQDNFPKFKENCHIFTRLITAVPSLREHFENVKKAIGIFVSTSIEEVFSFPFYNQLL<br>TQTQIDLYNQLLGGISREAGTEKIKGLNEVLNLAIQKNDETAHIIASLPHRFIPLFKQILSDRNTLSFILEEFKSDEEVIQSFCKYKTLLRNENVLETAEALFNELNSIDLTHIFISHKKLETIS<br>SALCDHWDTLRNALYERRISELTGKITKSAKEKVQRSLKHEDINLQEIISAAGKELSEAFKQKTSEILSHAHAALDQPLPTTLKKQEEKEILKSQLDSLLGLYHLLDWFAVDESNEVDPEFSARL<br>TGIKLEMEPSLSFYNKARNYATKKPYSVEKFKLNFQMPTLARGWDVNREKNNGAILFVKNGLYYLGIMPKQKGRYKALSFEPTEKTSEGFDKMYYDYFPDAAKMIPKCSTQLKAVTAHFQTHTTP |

| | |
|---|---|
| | ILLSNNFIEPLEITKEIYDLNNPEKEPKKFQTAYAKKTGDQKGYREALCKWIDFTRDFLSKYTKTTSIDLSSLRPSSQYKDLGEYYAELNPLLYHISFQRIAEKEIMDAVETGKLYLFQIYNKDF<br>AKGHHGKPNLHTLYWTGLFSPENLAKTSIKLNGQAELFYRPKSRMKRMAHRLGEKMLNKKLKDQKTPIPDTLYQELYDYVNHRLSHDLSDEARALLPNVITKEVSHEIIKDRRFTSDKFFFHVPI<br>TLNYQAANSPSKFNQRVNAYLKEHPETPIIGIDRGERNLIYITVIDSTGKILEQRSLNTIQQFDYQKKLDNREKERVAARQAWSVVGTIKDLKQGYLSQVIHEIVDLMIHYQAVVVLENLNFGFK<br>SKRTGIAEKAVYQQFEKMLIDKLNCLVLKDYPAEKVGGVLNPYQLTDQFTSFAKMGTQSGFLFYVPAPYTSKIDPLTGFVDPFVWKTIKNHESRKHFLEGFDFLHYDVKTGDFILHFKMNRNLSF<br>QRGLPGFMPAWDIVFEKNETQFDAKGTPFIAGKRIVPVIENHRFTGRYRDLYPANELIALLEEKGIVFRDGSNILPKLLENDDSHAIDTMVALIRSVLQMRNSNAATGEDYINSPVRDLNGVCFD<br>SRFQNPEWPMDADANGAYHIALKGQLLLNHLKESKDLKLQNGISNQDWLAYIQELRNGRSSDDEATADSQHAAPPKKKRKVGGSGGSGGSGGSGGSGGSGGSGGSLEHHHHHH |
| **enCas 12a Ultra** | MTQFEGFTNLYQVSKTLRFELIPQGKTLKHIQEQGFIEEDKARNDHYKELKPIIDRIYKTYADQCLQLVQLDWENLSAAIDSYRKEKTEETRNALIEEQATYRNAIHDYFIGRTDNLTDAINKRH<br>AEIYKGLFKAELFNGKVLKQLGTVTTTEHENALLRSFDKFTTYFSGFYRNRKNVFSAEDISTAIPHRIVQDNFPKFKENCHIFTRLITAVPSLREHFENVKKAIGIFVSTSIEEVFSFPFYNQLL<br>TQTQIDLYNQLLGGISREAGTEKIKGLNEVLNLAIQKNDETAHIIASLPHRFIPLFKQILSDRNTLSFILEEFKSDEEVIQSFCKYKTLLRNENVLETAEALFNELNSIDLTHIFISHKKLETIS<br>SALCDHWDTLRNALYERRISELTGKITKSAKEKVQRSLKHEDINLQEIISAAGKELSEAFKQKTSEILSHAHAALDQPLPTTLKKQEEKEILKSQLDSLLGLYHLLDWFAVDESNEVDPEFSARL<br>TGIKLEMEPSLSFYNKARNYATKKPYSVEKFKLNFQRPTLARGWDVNREKNNGAILFVKNGLYYLGIMPKQKGRYKALSFEPTEKTSEGFDKMYYDYFPDAAKMIPKCSTQLKAVTAHFQTHTTP<br>ILLSNNFIEPLEITKEIYDLNNPEKEPKKFQTAYAKKTGDQKGYREALCKWIDFTRDFLSKYTKTTSIDLSSLRPSSQYKDLGEYYAELNPLLYHISFQRIAEKEIMDAVETGKLYLFQIYNKDF<br>AKGHHGKPNLHTLYWTGLFSPENLAKTSIKLNGQAELFYRPKSRMKRMAHRLGEKMLNKKLKDQKTPIPDTLYQELYDYVNHRLSHDLSDEARALLPNVITKEVSHEIIKDRRFTSDKFLFHVPI<br>TLNYQAANSPSKFNQRVNAYLKEHPETPIIGIDRGERNLIYITVIDSTGKILEQRSLNTIQQFDYQKKLDNREKERVAARQAWSVVGTIKDLKQGYLSQVIHEIVDLMIHYQAVVVLENLNFGFK<br>SKRTGIAEKAVYQQFEKMLIDKLNCLVLKDYPAEKVGGVLNPYQLTDQFTSFAKMGTQSGFLFYVPAPYTSKIDPLTGFVDPFVWKTIKNHESRKHFLEGFDFLHYDVKTGDFILHFKMNRNLSF<br>QRGLPGFMPAWDIVFEKNETQFDAKGTPFIAGKRIVPVIENHRFTGRYRDLYPANELIALLEEKGIVFRDGSNILPKLLENDDSHAIDTMVALIRSVLQMRNSNAATGEDYINSPVRDLNGVCFD<br>SRFQNPEWPMDADANGAYHIALKGQLLLNHLKESKDLKLQNGISNQDWLAYIQELRNGRSSDDEATADSQHAAPPKKKRKVGGSGGSGGSGGSGGSGGSGGSGGSLEHHHHHH |
| **enCas 12a*** | MTQFEGFTNLYQVSKTLRFELIPQGKTLKHIQEQGFIEEDKARNDHYKELKPIIDRIYKTYADQCLQLVQLDWENLSAAIDSYRKEKTEETRNALIEEQATYRNAIHDYFIGRTDNLTDAINKRH<br>AEIYKGLFKAELFNGKVLKQLGTVTTTEHENALLRSFDKFTTYFSGFYRNRKNVFSAEDISTAIPHRIVQDNFPKFKENCHIFTRLITAVPSLREHFENVKKAIGIFVSTSIEEVFSFPFYNQLL<br>TQTQIDLYNQLLGGISREAGTEKIKGLNEVLNLAIQKNDETAHIIASLPHRFIPLFKQILSDRNTLSFILEEFKSDEEVIQSFCKYKTLLRNENVLETAEALFNELNSIDLTHIFISHKKLETIS<br>SALCDHWDTLRNALYERRISELTGKITKSAKEKVQRSLKHEDINLQEIISAAGKELSEAFKQKTSEILSHAHAALDQPLPTTLKKQEEKEILKSQLDSLLGLYHLLDWFAVDESNEVDPEFSARL<br>TGIKLEMEPSLSFYNKARNYATKKPYSVEKFKLNFQMPTLARGWDVNREKNNGAILFVKNGLYYLGIMPKQKGRYKALSFEPTEKTSEGFDKMYYDYFPDAAKMIPKCSTQLKAVTAHFQTHTTP<br>ILLSNNFIEPLEITKEIYDLNNPEKEPKKFQTAYAKKTGDQKGYREALCKWIDFTRDFLSKYTKTTSIDLSSLRPSSQYKDLGEYYAELNPLLYHISFQRIAEKEIMDAVETGKLYLFQIYNKDF<br>AKGHHGKPNLHTLYWTGLFSPENLAKTSIKLNGQAELFYRPKSRMKRMAHRLGEKMLNKKLKDQKTPIPDTLYQELYDYVNHRLSHDLSDEARALLPNVITKEVSHEIIKDRRFTSDKFFFHVPI<br>TLNYQAANSPSKFNQRVNAYLKEHPETPIIGIDRGERNLIYITVIDSTGKILEQRSLNTIQQFDYQKKLDNREKERVAARQAWSVVGTIKDLKQGYLSQVIHEIVDLMIHYQAVVVLENLNFGFK<br>SKRTGIAEKAVYQQFEKMLIDKLNCLVLKDYPAEKVGGVLNPYQLTDQFTSFAKMGTQSGFLFYVPAPYTSKIDPLTGFVDPFVWKTIKNHESRKHFLEGFDFLHYDVKTGDFILHFKMNRNLSF<br>QRGLPGFMPAWDIVFEKNETQFDAKGTPFIAGKRIVPVIENHRFTGRYRDLYPANELIALLEEKGIVFRDGSNILPKLLENDDSHAIDTMVALIRSVLQMRNSNAATGEDYINSPVRDLNGVCFD<br>SRFQNPEWPMDADANGAYHIALKGQLLLNHLKESKDLKLQNGISNQDWLAYIQELRNKRPAATKKAGQAKKKKGAAALEHHHHHH |
| **enCas 12a- Hifi*** | MTQFEGFTNLYQVSKTLRFELIPQGKTLKHIQEQGFIEEDKARNDHYKELKPIIDRIYKTYADQCLQLVQLDWENLSAAIDSYRKEKTEETRNALIEEQATYRNAIHDYFIGRTDNLTDAINKRH<br>AEIYKGLFKAELFNGKVLKQLGTVTTTEHENALLRSFDKFTTYFSGFYRNRKNVFSAEDISTAIPHRIVQDNFPKFKENCHIFTRLITAVPSLREHFENVKKAIGIFVSTSIEEVFSFPFYNQLL<br>TQTQIDLYNQLLGGISREAGTEKIKGLNEVLALAIQKNDETAHIIASLPHRFIPLFKQILSDRNTLSFILEEFKSDEEVIQSFCKYKTLLRNENVLETAEALFNELNSIDLTHIFISHKKLETIS<br>SALCDHWDTLRNALYERRISELTGKITKSAKEKVQRSLKHEDINLQEIISAAGKELSEAFKQKTSEILSHAHAALDQPLPTTLKKQEEKEILKSQLDSLLGLYHLLDWFAVDESNEVDPEFSARL<br>TGIKLEMEPSLSFYNKARNYATKKPYSVEKFKLNFQMPTLARGWDVNREKNNGAILFVKNGLYYLGIMPKQKGRYKALSFEPTEKTSEGFDKMYYDYFPDAAKMIPKCSTQLKAVTAHFQTHTTP<br>ILLSNNFIEPLEITKEIYDLNNPEKEPKKFQTAYAKKTGDQKGYREALCKWIDFTRDFLSKYTKTTSIDLSSLRPSSQYKDLGEYYAELNPLLYHISFQRIAEKEIMDAVETGKLYLFQIYNKDF<br>AKGHHGKPNLHTLYWTGLFSPENLAKTSIKLNGQAELFYRPKSRMKRMAHRLGEKMLNKKLKDQKTPIPDTLYQELYDYVNHRLSHDLSDEARALLPNVITKEVSHEIIKDRRFTSDKFFFHVPI<br>TLNYQAANSPSKFNQRVNAYLKEHPETPIIGIDRGERNLIYITVIDSTGKILEQRSLNTIQQFDYQKKLDNREKERVAARQAWSVVGTIKDLKQGYLSQVIHEIVDLMIHYQAVVVLENLNFGFK<br>SKRTGIAEKAVYQQFEKMLIDKLNCLVLKDYPAEKVGGVLNPYQLTDQFTSFAKMGTQSGFLFYVPAPYTSKIDPLTGFVDPFVWKTIKNHESRKHFLEGFDFLHYDVKTGDFILHFKMNRNLSF<br>QRGLPGFMPAWDIVFEKNETQFDAKGTPFIAGKRIVPVIENHRFTGRYRDLYPANELIALLEEKGIVFRDGSNILPKLLENDDSHAIDTMVALIRSVLQMRNSNAATGEDYINSPVRDLNGVCFD<br>SRFQNPEWPMDADANGAYHIALKGQLLLNHLKESKDLKLQNGISNQDWLAYIQELRNKRPAATKKAGQAKKKKGAAALEHHHHHH |

***: enCas12a and enCas12a-Hifi with published NLS sequence (Kleinstiver BP., et al., 2019)**

**Supplementary Table 11 AsCas12a protein sequences used in this study.**
