# Supplementary material for: AsCas12a ultra nuclease facilitates the rapid generation of therapeutic cell medicines
Source: Nat Commun. 2021 Jun 23;12:3908. doi: 10.1038/s41467-021-24017-8 (PMC8222333; doi:10.1038/s41467-021-24017-8)
Supplement: Supplementary file 2 — Reporting Summary [file 41467_2021_24017_MOESM2_ESM.pdf]

nature research

Corresponding author(s): Christopher A. Vakulskas

Last updated by author(s): May 19, 2021

Reporting Summary

Nature Research wishes to improve the reproducibility of the work that we publish. This form provides structure for consistency and transparency in reporting. For further information on Nature Research policies, see our Editorial Policies and the Editorial Policy Checklist.

Statistics

For all statistical analyses, confirm that the following items are present in the figure legend, table legend, main text, or Methods section.

n/a	Confirmed	
☐	☒	The exact sample size (*n*) for each experimental group/condition, given as a discrete number and unit of measurement
☐	☒	A statement on whether measurements were taken from distinct samples or whether the same sample was measured repeatedly
☐	☒	The statistical test(s) used AND whether they are one- or two-sided<br>*Only common tests should be described solely by name; describe more complex techniques in the Methods section.*
☒	☐	A description of all covariates tested
☒	☐	A description of any assumptions or corrections, such as tests of normality and adjustment for multiple comparisons
☐	☒	A full description of the statistical parameters including central tendency (e.g. means) or other basic estimates (e.g. regression coefficient) AND variation (e.g. standard deviation) or associated estimates of uncertainty (e.g. confidence intervals)
☐	☒	For null hypothesis testing, the test statistic (e.g. *F*, *t*, *r*) with confidence intervals, effect sizes, degrees of freedom and *P* value noted<br>*Give P values as exact values whenever suitable.*
☒	☐	For Bayesian analysis, information on the choice of priors and Markov chain Monte Carlo settings
☒	☐	For hierarchical and complex designs, identification of the appropriate level for tests and full reporting of outcomes
☒	☐	Estimates of effect sizes (e.g. Cohen's *d*, Pearson's *r*), indicating how they were calculated

Our web collection on statistics for biologists contains articles on many of the points above.

Software and code

Policy information about availability of computer code

Data collection	No specific software was used for data collection
Data analysis	CRISPAltRations software (IDT) was applied to analyze amplicon sequencing data to determine editing efficiency. See Kurgan G, Turk R, Li H, et al. CRISPAltRations: a validated cloud-based approach for interrogation of double-strand break repair mediated by CRISPR genome editing. Mol Ther Methods Clin Dev. 2021;21:478-491. for detailed method description. The software is available through https://www.idtdna.com/pages/products/crispr-genome-editing/rhampseq-crispr-analysis-system<br><br>Custom R code was used to process Spec-seq/SEAM-seq data, as described in Zhang, L. et al. (2020). Nucleic Acids Research, 48(9): 5037–5053.<br><br>Published GUIDE-seq analysis tool V1.0.2 was applied to process GUIDE-seq data, which is available through https://github.com/aryeelab/guideseq. See Tsai SQ, Topkar VV, Joung JK, Aryee MJ. Open-source guideseq software for analysis of GUIDE-seq data. Nat Biotechnol. 2016;34(5):483 for more information

For manuscripts utilizing custom algorithms or software that are central to the research but not yet described in published literature, software must be made available to editors and reviewers. We strongly encourage code deposition in a community repository (e.g. GitHub). See the Nature Research guidelines for submitting code & software for further information.

Data

Policy information about availability of data

All manuscripts must include a data availability statement. This statement should provide the following information, where applicable:

- Accession codes, unique identifiers, or web links for publicly available datasets
- A list of figures that have associated raw data
- A description of any restrictions on data availability

Raw sequencing data has been submitted to SRA and released to public under BioProject: PRJNA730915.

Field-specific reporting

Please select the one below that is the best fit for your research. If you are not sure, read the appropriate sections before making your selection.

☒ Life sciences ☐ Behavioural & social sciences ☐ Ecological, evolutionary & environmental sciences

For a reference copy of the document with all sections, see nature.com/documents/nr-reporting-summary-flat.pdf

Life sciences study design

All studies must disclose on these points even when the disclosure is negative.

Sample size	The sample size was not predefined.
Data exclusions	No data was excluded from any analysis
Replication	Spec-seq/SEAM-seq were performed as independent biological replicates, i.e. the entire process from the very beginning was repeated twice. Key experiments involved in this study were performed with biological replicates or triplicates. Replications were successful without data exclusion. Bacterial selection and GUIDE-seq experiments were performed without replicates.
Randomization	No clinical research study with group randomization and blinding is involved in this study
Blinding	No clinical research study with group randomization and blinding is involved in this study

Reporting for specific materials, systems and methods

We require information from authors about some types of materials, experimental systems and methods used in many studies. Here, indicate whether each material, system or method listed is relevant to your study. If you are not sure if a list item applies to your research, read the appropriate section before selecting a response.

Materials & experimental systems

n/a	Involved in the study
☐	☒ Antibodies
☐	☒ Eukaryotic cell lines
☒	☐ Palaeontology and archaeology
☒	☐ Animals and other organisms
☒	☐ Human research participants
☒	☐ Clinical data
☒	☐ Dual use research of concern

Methods

n/a	Involved in the study
☒	☐ ChIP-seq
☐	☒ Flow cytometry
☒	☐ MRI-based neuroimaging

Antibodies

Antibodies used	APC anti-human HLA-A/B/C (BioLegend Clone W6/3, 1:100 dilution, cat #: 311409)<br>BV421 anti-human TCR-α/β (BioLegend Clone IP26, 1:100 dilution, cat #: 306721)<br>For EGFR CAR staining, cells were initially labeled with biotinylated human EGFR (Sino Biological, 1:100 dilution, cat #: 10001-H08H-B), then stained with streptavidin conjugated FITC (ThermoFisher, 1:200 dilution, cat #: SA1001).
Validation	Validation is provided on manufacturer's websites

Eukaryotic cell lines

Policy information about cell lines

Cell line source(s)	HEK293 / K562 / Jurkate cells were obtained from ATCC
Authentication	Cell lines were authenticated by the provider by STR profiling and Karyotyping
Mycoplasma contamination	Cell lines were free of Mycoplasma contamination, as determined by MycoAlert™ Mycoplasma Detection Kit (Lonza)
Commonly misidentified lines (See ICLAC register)	No commonly misidentified cell lines were used in this study

Flow Cytometry

Plots

Confirm that:

- [x] The axis labels state the marker and fluorochrome used (e.g. CD4-FITC).
- [x] The axis scales are clearly visible. Include numbers along axes only for bottom left plot of group (a 'group' is an analysis of identical markers).
- [x] All plots are contour plots with outliers or pseudocolor plots.
- [x] A numerical value for number of cells or percentage (with statistics) is provided.

Methodology

Sample preparation	All cells were grown in humidified 37 °C, 5% CO incubators. Human cell lines HEK293 and Jurkat were used for the development work of AsCas12a Ultra. HEK293 cells were cultured in Dulbecco's Modified Eagle Medium supplemented with 10% FBS and 100 U/mL penicillin -streptomycin solution (Thermo Fisher Scientific). Jurkat cells were maintained in RPMI-1640 medium with 10% FBS and 100 U/mL penicillin-streptomycin solution. CD4+ or CD8+ T cells were isolated from peripheral blood mononuclear cells as previously described. Cells were frozen in cryopreservation media at a density of 20 x 106 cells/ mL. Upon thawing, T cells were activated with anti-D3/anti-CD28 Dynabeads (ThermoFisher Scientific) for 48 hours. Cells were maintained at 1.3 x 106 cells/mL in proprietary base media supplemented with IL-2, IL-7, and IL-15. After removal of Dynabeads, T cells were expanded for an additional 72 hours before electroporation. NK cells were isolated from CD3-depeleted peripheral blood mononuclear cells as previously described, then frozen in cryopreservation media at 50 x 106 cells/mL. Upon thawing, NK cells were activated with G-Rex plates (Wilson Wolf). Cells were maintained in proprietary base media supplemented with IL-15. Media was refreshed every eight days, with cells harvested on day fourteen of culture for electroporation. HSCs were isolated from, and then frozen in cryopreservation media at a density of 10 x 106 cells/mL. Upon thawing, HSCs were maintained at 1.5 x 106 cells/mL in proprietary base media supplemented with SFC, TPO, and FLT3. iPSCs were cultured on Vitronectin-coated 96-well plates in E8 media supplemented with Clone R and prepared for electroporation as described previously [42].Cells were centrifuged at 526 x g for five minutes, resuspended in staining buffer, incubated at 4 °C for 30 minutes, and resuspended in FACS buffer in preparation for flow cytometry. Staining buffer consisted of the following antibodies diluted in FACS Buffer: APC anti-human HLA-A/B/C (BioLegend Clone W6/3, 1:100 dilution), BV421 anti-human TCR-α/β (BioLegend Clone IP26, 1:100 dilution) For EGFR CAR staining, cells were initially labeled with biotinylated human EGFR (Sino Biological, 1:100 dilution), then stained with streptavidin conjugated FITC (ThermoFisher, 1:200 dilution).
Instrument	Data was acquired using the LSRFortessa (Becton Dickinson)
Software	Data was analyzed using FlowJo V10
Cell population abundance	10,000 cells gathered under FSC/SSC live gate for all measurements which was then followed by subgating for relevant markers depending on experiment
Gating strategy	Gated FSC/SSC and then subgated for relevant markers depending on those specific markers being assessed in each experiment. mCherry channel is PE, GFP channel is FITC, HLA-staining is APC channel, BV421 channel is TCR, and FITC channel is used for EGFR CAR detection. Labels will be added to axes during revision.

- [x] Tick this box to confirm that a figure exemplifying the gating strategy is provided in the Supplementary Information.
